# Immunogenicity Is Not Improved by Increased Antigen Dose or Booster Dosing of Seasonal Influenza Vaccine in a Randomized Trial of HIV Infected Adults

**DOI:** 10.1371/journal.pone.0017758

**Published:** 2011-03-25

**Authors:** Curtis Cooper, Anona Thorne, Marina Klein, Brian Conway, Guy Boivin, David Haase, Stephen Shafran, Wendy Zubyk, Joel Singer, Scott Halperin, Sharon Walmsley, on behalf of the CIHR Canadian HIV Trials Network Influenza Vaccine Research Group

**Affiliations:** 1 University of Ottawa Division of Infectious Diseases, Ottawa, Canada; 2 CIHR Canadian HIV Trials Network, Vancouver, Canada; 3 McGill University, Montreal, Canada; 4 University of British Columbia, Vancouver, Canada; 5 Université Laval, Quebec City, Canada; 6 Dalhousie University, Halifax, Canada; 7 University of Alberta, Edmonton, Canada; 8 University of Toronto, Toronto, Canada; Statens Serum Institute, Denmark

## Abstract

**Introduction:**

The risk of poor vaccine immunogenicity and more severe influenza disease in HIV necessitate strategies to improve vaccine efficacy.

**Methods:**

A randomized, multi-centered, controlled, vaccine trial with three parallel groups was conducted at 12 CIHR Canadian HIV Trials Network sites. Three dosing strategies were used in HIV infected adults (18 to 60 years): two standard doses over 28 days, two double doses over 28 days and a single standard dose of influenza vaccine, administered prior to the 2008 influenza season. A trivalent killed split non-adjuvanted influenza vaccine (Fluviral™) was used. Serum hemagglutinin inhibition (HAI) activity for the three influenza strains in the vaccine was measured to assess immunogenicity.

**Results:**

297 of 298 participants received at least one injection. Baseline CD4 (median 470 cells/µL) and HIV RNA (76% of patients with viral load <50 copies/mL) were similar between groups. 89% were on HAART. The overall immunogenicity of influenza vaccine across time points and the three influenza strains assessed was poor (Range HAI ≥40 = 31–58%). Double dose plus double dose booster slightly increased the proportion achieving HAI titre doubling from baseline for A/Brisbane and B/Florida at weeks 4, 8 and 20 compared to standard vaccine dose. Increased immunogenicity with increased antigen dose and booster dosing was most apparent in participants with unsuppressed HIV RNA at baseline. None of 8 serious adverse events were thought to be immunization-related.

**Conclusion:**

Even with increased antigen dose and booster dosing, non-adjuvanted influenza vaccine immunogenicity is poor in HIV infected individuals. Alternative influenza vaccines are required in this hyporesponsive population.

**Trial Registration:**

ClinicalTrials.gov NCT00764998

## Introduction

HIV infection is associated with deficiencies in both humoral and cell-mediated immunity, which can alter the course of common infections and influence vaccine immunogenicity.[1],[2], [3], [4], [5] While highly active antiretroviral therapy (HAART) partially restores these deficiencies, HIV-infected persons remain at increased risk for morbidity from infectious diseases, especially if the ability to generate antigen-specific responses remains impaired.[6]

HIV infection predisposes individuals to increased susceptibility to influenza, prolonged viral replication and shedding, longer duration of influenza symptoms and higher influenza-related mortality.[3], [7], [8], [9] The risk for influenza-related death is estimated to be 9.4–14.6 per 10,000 in persons with AIDS, compared with 0.09–0.10 per 10,000 among healthy adults aged 25 to 54 years and 6.4–7.0 per 10,000 among the elderly.[10] In another study, the risk for cardiopulmonary hospitalizations among women with HIV infection was higher during influenza seasons.[11]

Controlled trials of single dose inactivated influenza vaccine in HIV-infected adults conducted both in the pre- and post-HAART eras have demonstrated safety but suboptimal antibody response.[2], [3] [12], [13] The likelihood of achieving seroprotective antibodies is particularly poor in those with advanced HIV disease.[7], [14], [15] Vaccine immunogenicity is better in HIV seropositive persons with minimal or no AIDS-related symptoms and high CD4 counts.[14], [15], [16], [17],[18] However, even in antiretroviral treated HIV patients with high influenza vaccination rates, protection from influenza disease is deficient.[19] Although the use of booster dosing and increased vaccine antigen dose have been assessed in the past, the results are conflicting, based on pre-HAART populations and limited by small sample size.[14], [20]

Definitive studies of alternative influenza vaccination strategies in this population are required. To this end, we evaluated the efficacy of increased vaccine antigen dose and the administration of a vaccine booster dose in a representative HIV study population.

## Methods

The protocol for this trial and supporting CONSORT checklist are available as supporting information; see Checklist S1, Flowchart S1, and Protocol S1.

### Population and Setting

A randomized, multi-centered, controlled, vaccine study with three parallel groups was conducted. HIV-infected volunteers, in otherwise stable health, aged 18 to 60 years, were recruited at twelve Canadian Institutes of Health Research Canadian HIV Clinical Trials Network sites located across Canada (see Acknowledgements for list of contributing sites). Enrolment began following research ethics approval obtained at each individual site. Informed, written consent was obtained from each participant. Exclusion criteria included: receipt or anticipated requirement of blood products, vaccine, or immunoglobulin preparation within one month of study vaccine administration until completion of study, use of immunosuppressive therapy or immune modulators, dialysis, autoimmune disease, alcohol consumption ≥4 drinks per day, history of cancer with the exception of cutaneous cancers including Kaposi Sarcoma, basal cell carcinoma and non-invasive HPV-related malignancy, known or suspected hypersensitivity to any component of the study vaccines, including chicken eggs or egg products and Thimerosol, history of immediate hypersensitivity reaction and/or reaction resulting in neurological symptoms to a previous dose of any influenza vaccine, or presentation with or any recent history (within 24 hours) of any febrile illness (>38°C) or symptoms of significant local or systemic infection. There were no exclusion criteria for antiretroviral use, HIV viral load or CD4 T lymphocyte count.

### Vaccine, Dosing and Immunogenicity Testing

The vaccine used was the 2008 seasonal trivalent killed split non-adjuvanted influenza vaccine (Fluviral™, GSK, Laval, Canada) containing A/Brisbane/59/2007 (H1N1), A/Uruguay/716/2007 (H3N2), and B/Florida/4/2006. Subjects recruited at each site were centrally randomized by the Canadian HIV Trials Network to one of three groups. Participants and all study staff were blinded to allocation, except for the individual who prepared the vaccine who had no direct contact with study participants. Group 1 received one adult dose of influenza vaccine (0.5 mL or 15 µg HA) between October 1st and November 15th 2008, followed by a booster influenza vaccine administered 28 days later. Group 2 received one double dose of influenza vaccine (1.0 mL or 30 µg HA) during the same interval, followed by a booster double dose of vaccine administered 28 days later. Group 3 received a single adult dose (0.5 mL or 15 µg HA) of influenza vaccine. Placebo injections were not utilized in this study. Randomization was stratified by CD4 T lymphocyte count (<200 cells/µL versus ≥200 cells/µL).

Blood samples were centrifuged and the sera from each were aliquoted into vials (minimum 2.0 ml/vial) for frozen storage at −80°C. Once all study specimens were collected, three sets of aliquots of each serum sample were transported frozen to the laboratory (GB) for hemagglutination inhibition (HAI) titre evaluation. HAI titres were measured according to WHO standard protocol.[21] Briefly, non-specific inhibitors were removed from serum by overnight treatment with receptor destroying enzyme (Denka Seiken, Tokyo, Japan). Physiologic saline solution was then added to achieve a 1∶10 dilution, followed by incubation with packed guinea pig red blood cells (GRBC) (Lampire Biological Laboratories Inc., Pipersville, PA) at 4°C for 60 min to remove non-specific agglutinins. Treated serum was serially diluted in 25 µl of PBS and then mixed with an equal volume of PBS containing 4 hemagglutinin units of A/Brisbane/59/2007 (H1N1), A/Uruguay/716/2007 (H3N2) or B/Florida/4/2006 viruses. After 30 min of incubation at room temperature, 50 µl of 1% GRBC solution was added to the mixture and incubated for 45–60 min before evaluation of hemagglutination. The HAI titer was recorded as the reciprocal of the last dilution that inhibited hemagglutination.

### Flu-like Illness

All subjects developing febrile respiratory syndromes during the 20-week period following initial influenza vaccination were asked to report to clinic for assessment. A respiratory illness symptom diary was also provided to capture events. Respiratory infections were defined as a temperature >38.0°C associated with any one or more of the following clinical symptoms: feverishness/chills; cough; tachypnea/dyspnea; wheezing/stridor; rhinorrhea; sore throat; myalgias. An in-house real-time multiplex reverse-transcriptase PCR assay was utilized to identify influenza in those who presented while symptomatic.[22]

### Adverse Events

All subjects were observed at the site clinic for 15 minutes following each study vaccination to monitor for anaphylactic reactions, as well as for any other local and/or systemic reactions, to the vaccine. Subjects were then provided with, and instructed how to use, a thermometer, a transparent ruler and a diary to continue to monitor for any local and/or systemic reactions to the vaccine for 7 days following the study vaccination. Subjects were asked to record their temperature (°C), any redness or swelling at or near the injection site (mm), the severity of symptoms: pain (at or near the injection site), malaise, headache, fatigue (none, mild, moderate, severe), and any other adverse events. They were also asked to contact the clinic if they were experiencing a fever. A new diary was provided at each study visit to record any events that occurred during the time before the next visit.

### Statistical Analysis

The primary objective was to compare the immunogenicity of each of the two novel vaccination strategies with the traditional strategy of a single standard dose for each of the three influenza strains. The proportion of subjects achieving doubling of HAI titre from baseline at week 8 was selected as the primary outcome given the anticipated potential for diminished immunogenicity in this vaccine hyporesponsive population. Sample size calculations for this study were based on the comparison of two independent proportions using a two-tailed α of 0.05 and a (1-β) of 0.90. The control rate of doubling of titres was estimated to be 50%, and it was hypothesized that the modified doses of vaccine would improve the proportion of those doubling titre levels to 75%, an improvement of 25%.

As recommended by the Committee for Proprietary Medicinal Products (CPMP) [23], the proportion achieving seroconversion (quadrupling of HAI titre from baseline) and seroprotection (HAI titre ≥40 and ≥80 in those with baseline HAI titres ≤10) were assessed and compared by randomized group at weeks 4, 8, and 20. These benchmarks are associated with high level protection from clinical illness resulting from influenza infection. Seroconversion proportions over 40% and seroprotection titres ≥40 in 70% of recipients are standard targets required for approval of seasonal influenza vaccines. Geometric mean titres (GMT) at these time points and geometric mean ratios (GMR) with baseline were calculated and compared between groups. As per protocol, two pair wise comparisons were conducted for each outcome: 1) single dose plus booster versus single dose only, and 2) double dose plus booster versus single dose only. Proportions were compared using chi-square tests and GMT by t-tests. Missing values were imputed for week 8 outcomes only as follows: if an outcome, e.g. doubling of titres from baseline, was positive at weeks 4 and 20, it was considered to be positive at week 8 as well. Otherwise, missing responses were considered to be negative outcomes.

Multivariable logistic regression was used to explore the effects of key potential predictors of immunogenicity outcomes. For each outcome, all variables with p-values <0.15 in individual models controlling for treatment group were entered into multivariable regression models.

Secondary outcomes included self-reported influenza-like illness and PCR-confirmed influenza A and B identified from nasopharyngeal swab. The original plan was to compare proportions between groups as for the titre outcomes, but since the number of events was unexpectedly small, simple descriptions were used instead. All analyses were done using SAS (Statistical Analysis Software), Version 9.1.3.

## Results

### Study Population and Disposition

Baseline characteristics were well balanced between groups (Table 1). The mean age was 47 (SD 8.5) years. The majority were male and on HAART with HIV RNA levels below detection (<50 copies/mL). The baseline median CD4 T lymphocyte count was 470 cells/µL. Despite a high proportion having been vaccinated the previous year (84%), most participants (A/Brisbane: 67%, A/Uruguay: 72%, B/Florida: 56%) had HAI titres ≤ 10 at baseline.

**Table 1:** Baseline characteristics.

	Standard Dose plus Booster	Double Dose plus Booster	Single Standard Dose	Overall
	(n = 100)	(n = 104)	(n = 94)	
**Male**	88%	92%	90%	90%
**White**	79%	81%	83%	81%
**Antiretroviral Therapy at Time of Vaccination**	92%	86%	88%	89%
**HIV RNA** **<50 copies/mL**	79%	72%	77%	76%
**CD4 Count** **<200 cells/**µ**L**	10%	11%	7%	9%
**Influenza Vaccine in the Previous Year**	80%	85%	88%	84%
**HCV Co-Infection**	15%	12%	6%	11%
**HBV Co-Infection**	8%	4%	2%	5%
**Current Smokers**	47%	37%	44%	42%

Two hundred and ninety-eight participants were randomized, 297 received the first vaccination at baseline, and 281 returned for the follow-up visit 28 days (+/− 8 days) later. HAI titre measurements were unavailable for 6% of patients at week 4 and 9% at weeks 8 and 20. The distribution of missing values was balanced across treatment groups. For those missing week 8 titre values, a positive primary outcome was imputed for 4 of 25 patients missing A/Brisbane strain data, 2 of 16 patients missing A/Uruguay strain results, and 6 of 29 patients without B/Florida strain titres.

### Vaccine Immunogenicity

#### Overall Immunogenicity

**Figure 1:**
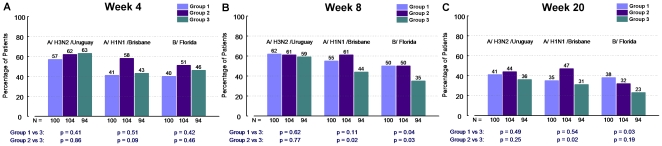
Proportion of patients with doubling of HAI titres. The proportion of vaccine recipients with doubling of HAI titres are described at week 4 (i.e. 4 weeks following the initial vaccination), week 8 (i.e. 8 weeks following the initial vaccination and 4 weeks following the booster dose in groups 1 and 2), and week 20. The HAI titre response is described for each of the three antigens included in the administered vaccine (A/H3N2/Uruguay, A/H1N1/Brisbane, B/Florida). Group 1 (single dose followed by single dose booster at week 4), Group 2 (double dose followed by double dose booster at week 4) and Group 3 (single dose without booster at week 4) are depicted.

**Figure 2:**
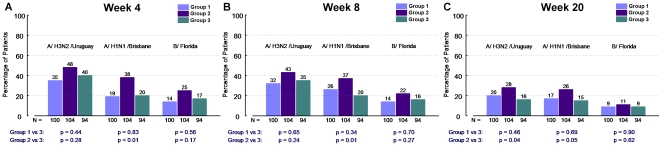
Proportion of patients achieving seroconversion (quadrupling of HAI titres). The proportion of vaccine recipients with a quadrupling of HAI titres are described at week 4 (i.e. 4 weeks following the initial vaccination), week 8 (i.e. 8 weeks following the initial vaccination and 4 weeks following the booster dose in groups 1 and 2), and week 20. The HAI titre response is described for each of the three antigens included in the administered vaccine (A/H3N2/Uruguay, A/H1N1/Brisbane, B/Florida). Group 1 (single dose followed by single dose booster at week 4), Group 2 (double dose followed by double dose booster at week 4) and Group 3 (single dose without booster at week 4) are depicted.

**Figure 3:**
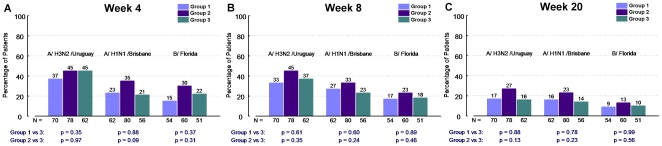
Patients with baseline HAI titres ≤ 10: proportion achieving seroprotection (titres ≥40). The proportion of vaccine recipients with baseline HAI titres ≤10 achieving seroprotection (titres ≥40) are described at week 4 (i.e. 4 weeks following the initial vaccination), week 8 (i.e. 8 weeks following the initial vaccination and 4 weeks following the booster dose in groups 1 and 2), and week 20. The HAI titre response is described for each of the three antigens included in the administered vaccine (A/H3N2/Uruguay, A/H1N1/Brisbane, B/Florida). Group 1 (single dose followed by single dose booster at week 4), Group 2 (double dose followed by double dose booster at week 4) and Group 3 (single dose without booster at week 4) are depicted.

Overall vaccine immunogenicity was poor, even by less stringent doubling of titre criteria (Figure 1, panel A, B, C). CPMP seroconversion criteria (i.e. quadrupling of titres in >40% of recipients) was met only in double dose and double dose booster recipients for A/Uruguay (Figure 2, panel A, B). Seroprotection (i.e. HAI titres ≥40 in >70% of recipients) was not achieved with any of the three strategies evaluated (Figure 3, panel A, B). GMT criteria (i.e. ≥2.5-fold increase in GMT from baseline) was only met for A/Uruguay at week 8 (standard dose plus booster: 2.6, double dose plus booster: 2.9, standard dose: 2.4).

#### Booster Dosing

The effect of booster dosing was evaluated at weeks 8 (4 weeks post booster) and 20 (16 weeks post booster). The overall HAI titres achieved were disappointing. However, some evidence of benefit with booster dosing was detected. The administration of a double dose plus double dose booster increased the proportion of those achieving a doubling of HAI titres from baseline at week 8 for A/Brisbane (61% vs 44%, p = 0.02) and B/Florida (50% vs 35%, p = 0.03) (Figure 1, panel B) and at week 20 (47% vs 31%, p = 0.02) for A/Brisbane (Figure 1, panel C) compared to recipients of a single standard vaccine dose. Administration of a standard dose plus booster dose increased the proportion of those achieving a doubling in HAI titres from baseline for B/Florida at week 8 (50% vs 35%, p = 0.04) (Figure 1, panel B) and week 20 (38% vs 23%, p = 0.03) (Figure 1, panel C) compared to recipients of a standard dose of vaccine. The direction of effect for A/Brisbane was similar but not statistically significant at weeks 8 and 20 (Figure 1, panels B, C). Booster dosing did not improve HAI titre doubling for A/Uruguay.

Administration of a double dose plus double dose booster increased the proportion of those achieving seroconversion (4-fold increase in HAI from baseline) for A/Brisbane at weeks 8 (37% vs 20%, p = 0.01) (Figure 2, panel B) and 20 (26% vs 15%, p = 0.05) (Figure 2, panel C) compared to recipients of a standard vaccine dose. Similar trends were noted for the other two antigens. A standard dose booster did not increase the proportion of those achieving seroconversion at weeks 8 or 20 compared to a single standard dose of vaccine without booster (Figure 2, panels B, C).

Seroprotection was assessed in those with baseline HAI titres ≤10 (Figure 3). Although still low overall, the double dose plus booster strategy consistently demonstrated trends toward improved seroprotection at weeks 8 (Figure 3, panel B) and 20 (Figure 3, panel C) for all three antigens compared to a single standard dose. This was also observed for high seroprotective HAI titres (≥80) at week 8 for A/Uruguay (27% vs 11%, p = 0.02). A standard dose followed by a standard dose booster did not consistently improve these endpoint measures.

GMT and GMR were compared at weeks 8 and 20 to evaluate the effect of booster dosing (data not shown). Although the direction of effect consistently favored booster versus non-booster dosing strategies for the A/Brisbane and A/Uruguay strains, this was not statistically or clinically significant. The same was true for double dose versus standard dose booster recipients. GMT and GMR declined significantly irrespective of dosing strategy by week 20.

#### Increased Antigen Dose

Given the study design, the effect of an increased dose of vaccine (30 µg of each antigen) could be assessed and compared to standard dose (15 µg of each antigen) at week 4. Although not statistically significant, a trend toward increased HAI titre doubling was noted with A/Brisbane and B/Florida (Figure 1, panels A, B, C). Seroconversion rates for A/Brisbane at week 4 (Figure 2, panel A) were increased significantly and similar trends were noted for the other antigens. Week 4 seroprotection (HAI titre ≥40) was assessed in those with baseline HAI titres ≤10 (Figure 3, panel A). A trend favoring increased antigen dose was noted for A/Brisbane and B/Florida but not A/Uruguay (Figure 3, panel A). GMT titres were higher, although not statistically significant, at week 4 in double dose recipients (A/Brisbane: 32.9; A/Uruguay: 47.3; B/Florida: 32.0) compared to single (combined data from Groups 1 and 3 for A/Brisbane: 26.5; A/Uruguay: 38.8; B/Florida: 29.9) (p = 0.12, 0.22, 0.61, respectively).

#### Sub-group Analysis of HIV RNA Non-Suppressed Patients

**Figure 4:**
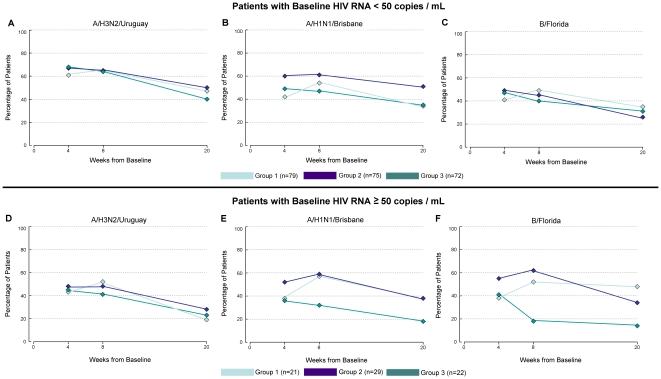
HAI titre doubling over time as a function of baseline HIV RNA suppression. The proportion of vaccine recipients achieving HAI doubling at week 4, 8 and 20 is described as a function of whether HIV RNA was below the lower limit of detection at baseline (<50 copies/mL) (n = 226) or above this level (n = 72). Each of the three antigens included in the administered vaccine (A/H3N2/Uruguay, A/H1N1/Brisbane, B/Florida) is considered. Group 1 (single dose followed by single dose booster at week 4), Group 2 (double dose followed by double dose booster at week 4) and Group 3 (single dose without booster at week 4) are depicted.

As planned *a priori*, the possible differential treatment effect for patients without HIV viral suppression was explored by means of a sub-group analysis, examining the differences in HAI titre doubling for the 72 patients with non-suppressed HIV viral load in comparison to the 226 with viral load suppression (Figure 4). Among HIV RNA non-suppressed patients, double dose vaccine appeared to improve HAI titre doubling at week 4 and booster dosing improved this measure at weeks 8 and 20 for each antigen, although the differences were not statistically significant. Similar trends were noted for seroprotection and seroconversion (data not shown). This trend was not apparent in those with HIV RNA suppression.

#### Predictors of Immunogenicity

Exploratory analyses were conducted to evaluate for factors predictive of vaccine immunogenicity. Multivariable logistical regression was controlled for baseline variables related to HIV therapy, HIV viral load, CD4 count, age, sex, weight, tobacco use, viral hepatitis co-infection, history of prior influenza vaccination, and lack of baseline influenza seroprotection (HAI titres ≤10). Note that adjustment for important prognostic factors had a minimal effect on estimates of treatment magnitude. At week 8, double dose plus booster recipients (in comparison with single standard dose recipients) were more likely to achieve HAI doubling for A/Brisbane [OR = 2.4 (1.3–4.4), p<0.01] and B/Florida [1.9 (1.0–3.5), p = 0.04] and seroconversion for A/Brisbane [2.2 (1.1–4.3), p = 0.03] as well as week 20 seroconversion for A/Uruguay [2.2 (1.1–4.7), p = 0.03] and A/Brisbane [2.1 (1.0–4.4), p = 0.05]. Baseline HAI titre >1∶10 was highly predictive of seroprotection (both HAI titres ≥40 and ≥80) at weeks 8 and 20 (all antigens), doubling of titres at week 8 (all antigens), and doubling of titres at week 20 (A/Uruguay and B/Florida).

Although several other isolated trends were noted with individual antigens or at specific time points, no other consistent immunogenicity predictors were identified. CD4 count was not found to predict immunogenicity when controlled for by baseline HIV RNA level and the other above-mentioned variables.

### Influenza-Like Illness

Only 28 subjects reported flu-like symptoms during the period of evaluation; these were evenly distributed across the three groups. Six PCR-confirmed cases of influenza were documented (A/Brisbane = 2; A/not subtyped = 2, B/not subtyped = 2). All recovered without complication.

### Adverse Events

Vaccinations were well tolerated without increased local reactogenicity as a consequence of increased antigen dose or booster dosing. None of the 8 serious adverse events reported were immunization-related. No HIV-related serious adverse events or HIV-related opportunistic infections were reported.

## Discussion

This randomized clinical trial evaluated two potential means of achieving improved immunogenicity in HIV seropositive individuals: the administration a booster vaccine dose and the use of increased antigen dose.[20] Current Centers for Disease Control and Prevention guidelines do not recommend either practice.[24] However, the studies on which these recommendations are based were conducted in the pre-HAART era, evaluated small sample sizes, were not randomized and did not assess clinical outcomes.[18], [25], [26] We evaluated HIV patients representative of most clinical settings in the developed world. Unfortunately, no clear, uniform and clinically significant benefit was identified with either immunization strategy.

The use of a booster dose in our analysis, either with standard dose or double dose, slightly improved immunogenicity with two of the three antigens evaluated compared to a single, standard dose of vaccine. This was most clearly evident in those without HIV RNA suppression at baseline (Figure 4). However, immunogenicity was suboptimal, irrespective of dosing strategy. Our work suggests that booster dosing with conventional influenza vaccine will not address the issue of poor immunogenicity in this vaccine hyporesponsive population. Although compelling, we do not believe that our results are robust enough to recommend booster dosing in those without HIV RNA suppression.

There is little literature evaluating the efficacy of increased influenza vaccine antigen dose in HIV infected patients. In a sentinel work, Kroon *et al* evaluated the effect of double dose immunization in a cohort of HIV infected patients and concluded that this strategy was ineffective in augmenting antibody response.[14] However, the comparison arm was not randomized, the sample size was small, and the study was conducted in the pre-HAART period. As such, the majority of participants were profoundly immune compromised. Therefore, the results may not be applicable to current HIV populations in the developed world. The majority of our study population was on antiretroviral therapy with virological suppression and CD4 counts well over 200 cells/µL. Despite a small increase in immunogenicity with administration of a double dose, our analysis is consistent with the findings of Kroon *et al.* Although higher antigen doses could be assessed, widespread use of an increased antigen dose would create vaccine supply issues. Therefore, the feasibility of this strategy is questionable, even if demonstrated to be effective.

Overall, the rates of HAI protection achieved by these strategies, assessed by various CPMP benchmarks of success [23], were disappointingly low in proportion and relatively short-lived. Even with a lower benchmark of immunogenicity (i.e. two fold increase in HAI titres), clear benefit was not detected. This speaks to the overall poor immunogenicity of influenza vaccine in those with HIV infection. Our work suggests that although increased antigen dosing may slightly increase immunogenicity four weeks after immunization when utilizing conventional vaccines, this increase is minimal. This finding is consistent with a recently published pandemic HIN1 study of adult immune competent individuals in which the use of increased vaccine dose did not improve measures of vaccine efficacy.[27] Other strategies, including the use of vaccine adjuvants, should be evaluated in an effort to achieve more substantive and long-lived success without the need for increased antigen dose.[28], [29]

Several limitations are acknowledged. The small sample size likely influenced our ability to fully evaluate the influence of several key variables on the primary outcome measure. However, this was the largest randomized controlled trial of influenza vaccine immunogenicity in HIV patients ever conducted. Because of the relatively low incidence of influenza in Canada during the 2008–2009 season, insufficient cases were detected to allow for evaluation of the influences of booster dosing or increased vaccine dose on burden of influenza infection. We did not collect data on HIV RNA levels or CD4 T lymphocyte counts during the course of the study. However, it was our judgment that the safety of trivalent split non-adjuvanted influenza vaccine in the HIV population was already well-established.[2], [3], [12], [13] CPMP criteria for immunogenicity have not been validated in those living with HIV. However, it seemed reasonable to consider these well accepted criteria for evaluating immunogenicity in addition to the utilization of a lower HAI criteria (i.e. doubling of HAI titres).

Our work has demonstrated the safety of two alternate influenza vaccine strategies in a HIV population including increased antigen dose and the use of booster dosing. Although this study demonstrated a slight benefit with increased antigen dose followed by booster dosing in achieving and maintaining seroprotective HAI titres in this immune compromised population, the gain was minimal, inconsistent, and the overall immunogenicity was poor. Other vaccine strategies, including the use of adjuvants, are currently under evaluation.

## Supporting Information

Checklist S1 **CONSORT Checklist.**
(DOC)

Flowchart S1 **CONSORT Flowchart.**
(DOC)

Protocol S1 **Trial Protocol.**
(PDF)
