# Supplementary material for: Immunogenicity Is Not Improved by Increased Antigen Dose or Booster Dosing of Seasonal Influenza Vaccine in a Randomized Trial of HIV Infected Adults
Source: PLoS One. 2011 Mar 25;6(3):e17758. doi: 10.1371/journal.pone.0017758 (PMC3064575; doi:10.1371/journal.pone.0017758)
Supplement: Flowchart S1 — CONSORT Flowchart. (DOC) [file pone.0017758.s002.doc]

**CONSORT 2010 Flow Diagram**

**Allocation**

**Analysis**

**Follow-Up**

**Enrollment**

Assessed for eligibility (n=NA)

Excluded (n=NA)

♦  Not meeting inclusion criteria (n=NA)

♦  Declined to participate (n=NA)

♦  Other reasons (n=NA)

Analysed (n= 100)
♦ Excluded from analysis (n= 0)

Lost to follow-up (reasons unknown) (n= 6)

Discontinued intervention (n= 2)

(these 2 patients withdrew)

Allocated to intervention 1 (n= 100)

♦ Received allocated intervention (n= 95)

♦ Did not receive allocated intervention (n= 5)

(2 patients withdrew and 3 were lost to follow-up, so these 5 did not receive the second vaccination)

Lost to follow-up (reasons unknown) (n= 7)

Discontinued intervention (n= 3)

(Vaxigrip was inadvertently administered to 1 subject at week 4 visit; the other 2 withdrew)

Allocated to intervention 2 (n= 104)

♦ Received allocated intervention (n= 101)

♦ Did not receive allocated intervention (n= 3)

(all 3 were lost to follow-up and did not receive the second vaccination)

Analysed (n= 104)
♦ Excluded from analysis (n= 0 )

Randomized (n=298)

Lost to follow-up (reasons unknown) (n= 3)

Discontinued intervention (n= 4)

(3 patients withdrew; 1 patient was non-compliant)

Allocated to intervention 3 (n= 94)

♦ Received allocated intervention (n= 93)

♦ Did not receive allocated intervention (n= 1)

(lost to follow-up; patient did not return to clinic for first vaccination)

Analysed (n= 93)
♦ Excluded from analysis (n= 1)

(no data for this patient, since no vaccination was given and no HI titre measurements were taken)
