# Supplementary material for: Immunogenicity Is Not Improved by Increased Antigen Dose or Booster Dosing of Seasonal Influenza Vaccine in a Randomized Trial of HIV Infected Adults
Source: PLoS One. 2011 Mar 25;6(3):e17758. doi: 10.1371/journal.pone.0017758 (PMC3064575; doi:10.1371/journal.pone.0017758)
Supplement: Protocol S1 — Trial Protocol. (PDF) [file pone.0017758.s003.pdf]

Title:	A Controlled Trial to Assess the Immunogenicity and Efficacy of Three Influenza Vaccine Dosing Strategies in HIV Infected Adults
Protocol Number:	CTN 237
Date:	June 04, 2008
Version:	2.0
Principal Investigator:	Dr. Curtis Cooper<br>The Ottawa Hospital<br>501 Smyth Road, Room G-12<br>Ottawa, Ontario, K1H 8L6
Investigators:	Dr. Marina Klein, The Montreal Chest<br>Dr. Anita Rachlis, Sunnybrook Hospital<br>Dr. Marek Smieja, Hamilton St. Joseph's Healthcare/McMaster University<br>Dr. Don Kilby, U Ottawa Health Services<br>Dr. Colin Kovacs, The Maple Leaf Clinic<br>Dr. Sharon Walmsley, Toronto General<br>Dr. David Haase, Victoria General Hospital, QEII HSC,<br>Dr. John Gill, Southern Alberta HIV Clinic, Sheldon Chumir Health Centre<br>Dr. Stephen Shafran, University of Alberta, Royal Alexander Hospital<br>Dr. Brian Conway, Pender Clinic, Vancouver<br>Dr. Julio Montaner, John Ruedy Immunodeficiency Clinic
	Collaborators: Dr. Dean Fergusson, Dr. Ed Mills, Dr. Aslam Anis, Dr. Scott Halperin, Dr. Guy Boivin
Sponsor:	Ottawa Health Research Institute
Study Vaccine:	Fluviral, a licensed vaccine obtained from GlaxoSmithKline.
Central Laboratory:	Dr. Boivin, Laval University
Study Management Group:	Canadian HIV Trials Network (CTN), Vancouver BC
Medical Monitor:	CTN Chief Scientific Officer

The document contains information which is of a confidential, trade-secret and/or proprietary nature. It is not to be disclosed to any other person or party without the prior written approval of: Dr. Curtis Cooper.

APPROVED
IND: [illegible]
S & IL: [signature] June 04.08
CTN

Table of Contents

PROTOCOL SUMMARY **5**

LIST OF ABBREVIATIONS **6**

1. INTRODUCTION **7**

1.1. BACKGROUND 7
1.1.1 Current Efficacy of Influenza Vaccination *7*
1.1.2 Burden of Influenza Disease in HIV *7*
1.1.3 Efficacy of Influenza Vaccination is HIV *8*
1.1.4 Efficacy of Booster Influenza Vaccination in HIV *9*
1.1.5 A paucity of published evidence regarding efficacy of double dose influenza vaccination in immune compromised patients *10*
1.1.6 Systematic Reviews *11*
1.2 RATIONALE 11
1.2.1 Why is a trial needed now? *12*
1.3 POTENTIAL RISKS AND BENEFITS 12
1.3.1 Potential Risks and Discomforts: *12*
1.3.2 Potential Benefits *13*

2. STUDY OBJECTIVES **13**

2.1 PRIMARY OBJECTIVE 13
2.2 SECONDARY OBJECTIVES 13

3. ELIGIBILITY CRITERIA **13**

3.1 INCLUSION CRITERIA 13
3.2 EXCLUSION CRITERIA 13

4. STUDY DESIGN **14**

4.1 DESCRIPTION 14
4.2 TREATMENT GROUPS 14
4.3 RANDOMIZATION 17
4.4 STUDY ENDPOINTS 17
4.4.1 Primary Endpoint *17*
4.4.2 Secondary Endpoints *17*

5. EXPECTED DURATION OF SUBJECT PARTICIPATION **18**

6. EARLY TERMINATION OR WITHDRAWAL **18**

7. PARTICIPATION OF WOMEN AND CHILDREN: **18**

7.1 WOMEN 18
7.2 CHILDREN 18

8. STUDY MEDICATION **18**

8.1 STUDY MEDICATION DESCRIPTION 18
8.2 POTENTIAL SIDE EFFECTS 19
8.3 FORMULATION, PACKAGING, AND LABELING 19
8.4 STUDY VACCINE AND ACCOUNTABILITY PROCEDURES 19

9. CONCOMITANT MEDICATIONS/NATURAL REMEDIES/FOODS **19**

10. CONCOMITANT ALCOHOL (PLEASE SEE EXCLUSION CRITERIA) **19**

11. PROHIBITED MEDICATIONS AND PROCEDURES **20**

12. STUDY EVALUATIONS/PROCEDURES **20**

12.1 CLINICAL EVALUATIONS ... 20
12.1.1 General Clinical Evaluation ... 20
12.1.2 Clinical Evaluation for Influenza Infection ... 20
12.2 LABORATORY EVALUATIONS AND SPECIMEN COLLECTION ... 21
12.2.1 Immunogenicity Laboratory Measurements ... 21
12.2.2 Whole Blood Samples for Evaluation of Cell Mediated Immunity (CMI, Immunogenicity) ... 21
12.2.3 Nasopharyngeal Swab for Laboratory Assessment of Respiratory Illness ... 22
12.3 STORED RESEARCH SPECIMENS ... 22
12.3.1 Use of Stored Specimens ... 22
12.3.2 Disposition of Stored Specimens ... 23
12.4 QUESTIONNAIRES / DIARIES ... 23

13. STUDY VISITS ... 23

13.1 SCREENING (VISIT 1) ... 23
13.2 RANDOMIZATION AND VACCINATION (VISIT 2, WEEK 0) ... 24
13.3 BOOSTER VACCINATION DAY (VISIT 3, WEEK 4) ... 24
13.4 POST VACCINATION VISITS: ... 25
13.5 UNSCHEDULED VISIT DUE TO RESPIRATORY SYMPTOMS ... 25
13.6 EARLY TERMINATION VISIT ... 26

14. ASSESSMENT OF SAFETY ... 26

14.1 SAFETY MONITORING ... 26
14.2 ADVERSE EVENTS (AE'S) ... 26
14.3 LOCAL/SYSTEMIC REACTOGENICITY ... 27
14.4 RECORDING/DOCUMENTATION OF AE'S ... 27
14.5 SERIOUS ADVERSE EVENTS *(SAE'S)* ... 27
14.6 AE/SAE GRADING AND RELATIONSHIP ASSIGNMENT ... 28
14.6.1 AE/SAE Grading ... 28
14.6.2 Relationship Assessment ... 28
14.7 SAE REPORTING PROCEDURES ... 29
14.8 MONITORING OF AE'S/SAE'S ... 29

15. TRIAL MANAGEMENT AND ANALYSIS ... 29

16. STATISTICAL CONSIDERATIONS ... 30

16.1 RANDOMIZATION AND BLINDING PROCEDURES ... 30
16.2 SAMPLE SIZE CONSIDERATIONS/JUSTIFICATION ... 30
16.3 ROLE OF THE DATA AND SAFETY MONITORING BOARD ... 30

17. DETAILS OF STATISTICAL ANALYSIS ... 31

17.1 STATISTICAL ANALYSIS ... 31
17.2 PRIMARY ENDPOINT ... 31
17.3 SECONDARY ENDPOINTS ... 31
17.4 OTHER VARIABLES OF INTEREST ... 32
17.5 PLANNED SUBGROUP ANALYSES ... 32
17.6 OTHER ANALYTICAL ISSUES ... 32

18. QUALITY CONTROL AND QUALITY ASSURANCE ... 33

19. ETHICS/PROTECTION OF HUMAN SUBJECTS ... 33

19.1 ICH GUIDELINES/DECLARATION OF HELSINKI ... 33
19.2 RESEARCH ETHICS BOARD/INSTITUTIONAL REVIEW BOARD ... 33
19.3 INFORMED CONSENT PROCESS ... 33

20. PARTICIPANT CONFIDENTIALITY ... 34

21. EARLY TERMINATION OF THE PROTOCOL ... 34

22. TREATMENT DISCONTINUATION 34
23. PREMATURE STUDY DISCONTINUATION FOR AN INDIVIDUAL SUBJECT 35
24. PROTOCOL VIOLATIONS/DEVIATIONS 35
25. STUDY CONDUCT AND MONITORING 36
26. DATA MANAGEMENT RESPONSIBILITIES 36
27. SOURCE DOCUMENTS AND ACCESS TO SOURCE DATA/DOCUMENTS 36
28. DISCLOSURE AND PUBLICATION POLICY 37
29. REFERENCES 38
30. APPENDICES 43
APPENDIX A: DECLARATION OF HELSINKI 44
APPENDIX B: SUBJECT DIARY 50
APPENDIX C: STUDY PERIOD FLU SYMPTOM QUESTIONNAIRE 55
APPENDIX D: EMPLOYMENT, HEALTH CARE RESOURCE UTILIZATION, AND HEALTH UTILITIES INDEX QUESTIONNAIRE 58
APPENDIX E: MODEL INFORMED CONSENT 65
APPENDIX F: PRODUCT MONOGRAPH 75
APPENDIX G: CTN TOXICITY TABLE FOR GRADING ADVERSE EXPERIENCES 95

Protocol Summary

**Full Title**	A Controlled Trial to Assess the Immunogenicity and Efficacy of Three Influenza Vaccine Dosing Strategies in HIV Infected Adults	
**Short Title**	Influenza Vaccine in HIV	
**Clinical Phase**	Phase III	
**Sponsor**	Ottawa Health Research Institute	
**Conducted By**	Dr. Curtis Cooper, The Ottawa Hospital	
**Principal Investigator**	Dr. Curtis Cooper, The Ottawa Hospital	
**Primary Objective**	To assess the immunogenicity of three dosing strategies in HIV infected adults; two standard doses over 28 days, two double doses over 28 days and a single standard dose of influenza vaccine, administered prior to the 2008 influenza season.	
**Secondary Objectives**	(1) To assess the clinical efficacy in preventing flu-like illness, and (2) assess the clinical efficacy in preventing laboratory documented influenza.	
**Study Population**	Adults between the ages of 18 and $\leq$ 60 years with the diagnosis of HIV, and who are able to provide written informed consent.	
**Study Design**	Phase III, randomized, multi-centered, controlled, vaccine trial with three parallel groups. The study will be conducted at ~ 10 Canadian HIV Clinical Trial Network sites.	
**Sample Size**	N = 285 subjects total, divided into 3 treatment groups of 95 people each, with approximately 9 people per site.	
**Accrual Period**	July 1st 2008 to November 15 2008	
**Study Duration**	Start Date: October 1 2008	End Date: Data collection ends April 2009
**Endpoints**	The primary endpoint is Immunogenicity measured by haemagglutination inhibition (HI).<br>The secondary endpoints are (1) clinical signs and symptoms and (2) PCR assays and clinical evaluation for subjects who report with >38 C temperature.	
**Study Medication**	The vaccine Fluviral is a trivalent killed split non-adjuvanted influenza vaccine. Strains are selected yearly on recommendation of the World Health Organization.	

List of Abbreviations

ADR	Adverse Drug Reaction
AE	Adverse Event/Adverse Experience
AIDS	Acquired Immune Deficiency Syndrome
ARC	Aids Related Complex
CDC	Center for Disease Control
CIB	Clinical Investigator's Brochure
CIOMS	Council for International Organizations of Medical Sciences
CMI	Cell Mediated Immunity
CRF	Case Report Form
CRO	Contract Research Organization
DSMB	Data and Safety Monitoring Board
GCP	Good Clinical Practice
GMT	Geometric Mean Titres
H1N1	Hemagglutinin 1 Neuraminides 1, Subtype of influenza A
H3N2	Hemagglutinin 3 Neuraminides 2, Subtype of influenza A
HA	Hemagglutination Antibody
HAART	Highly Active Antiretroviral Therapy
HI	Hemagglutinin Inhibition
HIV	Human Immunodeficiency Virus
HPV	Human Papillomavirus
IB	Investigator's Brochure
ICF	Informed Consent Form
ICH	International Conference on Harmonization
IDE	Investigational Device Exemption
IEC	Independent or Institutional Ethics Committee
IM	Intramuscular
IND	Investigational New Drug
IRB/REB	Institutional Review Board/Research Ethics Board
N	Number (typically refers to participants)
NDA	New Drug Application
OTC	Over The Counter (medications)
PBMC	Peripheral Blood Mononuclear Cells
PI	Principal Investigator
PK	Pharmacokinetics
QA	Quality Assurance
QC	Quality Control
RCT	Randomized Clinical Trial
REB	Research Ethics Board
SAE	Serious Adverse Event/Serious Adverse Experience
SMC	Safety Monitoring Committee
SOP	Standard Operating Procedure
WHO	World Health Organization

1. INTRODUCTION

1.1. Background

1.1.1 Current Efficacy of Influenza Vaccination

Influenza vaccines are widely used each year in efforts to reduce the morbidity and mortality associated with seasonal influenza outbreaks. Although 70 to 90% of healthy, young adults develop post-vaccination hemagglutination antibody (HA) titres that are associated with increased protection against infection by the same or closely similar strains of influenza virus [8-12], the level of protection is reduced for more distantly related strains [13-16]. In the elderly [16-18] and other relatively immune compromised populations [19, 20] at risk for severe influenza disease, seroprotection against infection is also diminished. Furthermore, antibody titres wane rapidly following vaccination [21].

Anti-HA protects against both infection and disease with the homologous virus [22]. Resistance to infection is mediated in part by systemic hemagglutinin-specific immunoglobulin induced by parenteral immunization with inactivated virus [23, 24]. Previous investigation has demonstrated that hemagglutinin-specific immunoglobulin is predominately of the IgG1 and IgG3 subgroups [25-27]. In humans, complement activation and antibody-dependent cell-mediated cytotoxicity responses are most efficiently produced by IgG1 subclass antibodies [28, 29]. Both of these play important roles in viral neutralization. Hyporesponsiveness to influenza vaccination in the elderly may result, in part, from altered subclass hemagglutinin-specific IgG production [25].

Serum hemagglutinin inhibition (HI) activity is a test used to measure protection from influenza infection. Although there is no exact correlation between the titre of an individual antibody response and protection, groups of individuals with serum HI activity titres of ≥1:40 have higher levels of protection against infection than observed in groups with titres below this level. In the elderly and other immune compromised populations, serum HI titres above 1:80 may be required for improved protection [30].

1.1.2 Burden of Influenza Disease in HIV

HIV infection is associated with deficiencies in both humoral and cell-mediated immunity, which can potentially alter the course of common infections [31]. While highly active antiretroviral therapy (HAART) partially restore these deficiencies, HIV-infected persons may remain at increased risk for morbidity from viral illness, especially if the ability to generate antigen-specific responses remains impaired. Additional factors including high prevalence of smoking and chronic lung diseases may further predispose HIV-infected patients to respiratory tract infection [32].

There is limited published data related to the frequency and severity of influenza illness in HIV infected individuals. The risk for influenza-related death was estimated to be 9.4-14.6/10,000 in persons with AIDS, compared with 0.09-0.10/10,000 among all persons aged 25 to 54 years and 6.4 to 7.0/10,000 among persons aged ≥65 years [33]. In another study, the risk for cardiopulmonary hospitalizations among women with HIV

infection was higher during influenza seasons than during the peri-influenza periods [34]. In this report, the risk for hospitalization was higher for HIV-infected women than for women with other well-recognized high-risk conditions, including chronic heart and lung diseases.

Other reports indicate that influenza symptoms might be prolonged and the risk for complications from influenza increased [2, 3, 35]. HIV infection may predispose individuals to increased susceptibility to influenza, prolonged viral replication and shedding, longer duration of influenza symptomatology and higher influenza-related mortality [4]. Klein et al noted that influenza was responsible for at least 40% of all febrile respiratory illnesses among an outpatient population of HIV infected patients [36]. The typical presentation of illness consisted of rhinorrhea, pharyngitis and an absence of dyspnea. None of the patients diagnosed with influenza presented with pneumonia by radiograph. Ninety percent of these patients were on HAART with a median CD4 count of 325 and HIV RNA level of below 50 copies/mL.

1.1.3 Efficacy of Influenza Vaccination is HIV

Controlled trials of single dose inactivated influenza vaccine in HIV-infected adults demonstrated safety but suboptimal antibody response [37, 38]. It is key to note that these patients were not on HAART and as such, the results are not fully applicable to current HIV populations followed in Canada. Immunogenicity studies in post-HAART era patients have confirmed the production of protective antibodies in HIV-infected persons even though the antibody levels in HIV-infected persons appear to be lower than in those without HIV [4, 5]. In contrast, influenza vaccination has been demonstrated to produce substantial antibody titers in HIV seropositive persons with minimal or no AIDS-related symptoms and high CD4 counts [39-42]. A randomized, placebo-controlled trial determined that inactivated influenza vaccine was highly effective in preventing symptomatic, laboratory-confirmed influenza virus infection among HIV-infected persons with a mean of 400 CD4+ T-lymphocyte cells/mm^3 [43]. A limited number of persons with CD4+ T-lymphocyte cell counts of <200 were included in that study precluding conclusions related to clinical outcomes in the profoundly immune compromised HIV population. Clearly, the likelihood of achieving seroprotective antibodies is diminished with advanced HIV disease [41, 42]. A nonrandomized study among HIV-infected persons determined that influenza vaccination was less effective in preventing disease among persons with CD4 counts below 100 cells/µL and among those with HIV viral loads above 30,000 copies /mL [35].

A recent meta-analysis attempted to tabulate the clinical effectiveness of influenza vaccine to prevent disease in HIV-infected individuals [44]. Studies comparing the incidence of culture- or serologically-confirmed influenza or clinical influenza-like illness between vaccinated and unvaccinated HIV infected individuals were included. Four studies involving 646 HIV-infected subjects were identified. In each study, the incidence of influenza was lower in vaccinated subjects with a risk difference ranging from -0.48 (95% CI: -0.63, -0.34) to -0.15 (95% CI: -0.25, 0.05). The summary risk difference was -

0.27 (95%CI: -0.42, -0.11). Vaccine effectiveness ranged from 27% to 78%. By their calculations, one case of influenza would be prevented by vaccination of three to seven HIV-infected patients. This analysis is relevant to the typical HIV populations followed in Canadian clinics as 56% to 96% of those evaluated were on HAART. The median CD4 count was greater than 400 cells/µL in 3 of 4 of the selected evaluations. Unfortunately, this sample was too small to assess the influence of antiretroviral therapy, CD4 count or HIV viral load on vaccine effectiveness.

Not all studies have demonstrated such good influenza vaccination efficacy in HAART-treated populations with high CD4 counts [36]. Klein et al noted that influenza was responsible for at least 40% of all febrile respiratory illnesses among an outpatient population of HIV infected patients [36]. Of particular concern was that fact that the pre-respiratory illness influenza vaccination rate was 76%. As a consequence of conflicting data, reduced antibody response to influenza vaccine and uncertainty related to the efficacy in preventing influenza disease in HIV patients, alternative strategies to influenza vaccination in this population is required.

1.1.4 Efficacy of Booster Influenza Vaccination in HIV

One potential means of achieving higher seroprotective rates with vaccination may be to administer a booster dose of influenza vaccine [45]. The efficacy of a dose-booster strategy administered over a one month period was evaluated in a pre-HAART era population [43]. One hundred and nine patients were enrolled: HIV seronegative heterosexual (n=11), HIV seronegative homosexual men (n=20), asymptomatic HIV seropositive male (n=32), HIV seropositive men with AIDS-related complex (n=9) and HIV seropositive men with AIDS (n=37). CD4 counts were available in 67 HIV patients (asymptomatic: 527 ±252, ARC: 295±172, AIDS: 128±116). Baseline HIV RNA levels were not available. Thirteen AIDS patients were on zidovudine monotherapy at the time of vaccination. The median age in the groups ranged from 33 to 41 years. Recipients received 15µg of trivalent influenza virus subvirion vaccine from the same lot [A/Taiwan/1/86(H1N1), A/Leningrad/360/86(H3/N2), B/Ann Arbor/1/86]. The study was conducted between November 1987 and February 1988. The frequency of seroconversion for each influenza A vaccine antigen ranged from 55% to 75% after the first dose in HIV-infected patients. This increased marginally following the second dose (73%-80%). The ability of HIV-infected subjects to respond serologically to one or both antigens was inversely related to the severity of their HIV disease. Asymptomatic HIV patients with pre-vaccination antibody titres ≤ 1:16 achieved seroconversion as frequently as non-HIV infected participants (A/Taiwan: 80-84% versus 71%-93%, A/Leningrad: 92-100% versus 84%. B/Ann Arbor: 65-70% versus 37%). Patients with more advanced HIV disease had low seroconversion rates (A/Taiwan: 38-67%, A/Leningrad: 0-67%, B/Ann Arbor: 13-22%). The two dose schedule produced 4-fold or greater increase in HI antibodies to each of the vaccine antigens more often in HIV negative participants than those with AIDS/ARC (A/Taiwan: 90% versus 55%, p=0.07 ; A/Leningrad:84% versus 30%, p<0.01, B/Ann Arbor: 56% versus 20%, p=0.03). The booster had little or no effect in increasing the proportion with protective antibody levels in asymptomatic HIV patients or with those with ARC/AIDS. In conclusion, there was a

significantly lower antibody response to all antigens in AIDS/ARC patients compared to HIV seronegative controls. Post booster antibody titre was correlated with CD4 count ($p<0.03$). Of note, there was a trend that AIDS patients on zidovudine may have benefited from the booster. This final observation raises the issue of if, and to what degree, HAART perturbs the effect of booster influenza vaccination.
Iorio et al also concluded that booster dosing of influenza vaccine was ineffective at achieving higher antibody titres in a group of former injection drug using HIV seropositive individuals [46]. This work is again limited by small sample size and the fact that it was conducted on a pre-HAART population.

Although the CDC references the above data in support of the statement that administration of a booster dose is not beneficial in achieving greater seroprotective titres [47], there are multiple concerns which call into question whether this conclusion is justified. These studies were conducted in a pre-HAART era. The influence of current HIV treatment on influenza vaccine response is not well described. There is no literature available on the efficacy of a booster strategy in the post –HAART era. The sample sizes are small and the studies were not randomized. Furthermore, clinical outcomes were not assessed. As a consequence of these multiple shortcomings, we believe that evaluation of influenza booster dose strategy in HIV patients in the post-HAART era is necessary.

1.1.5 A paucity of published evidence regarding efficacy of double dose influenza vaccination in immune compromised patients

There is next to no literature evaluating the efficacy of increased influenza vaccine antigen dose in HIV infected patients. Kroon *et al* did evaluate the effect of double dose immunization in a cohort of HIV infected patients and concluded that this strategy was ineffective in augmenting antibody response [42]. However, there was no randomized comparison arm, the sample size was small, and the study was conducted in the pre-HAART period. As such, the majority of participants were profoundly immune compromised. No correlation between antibody production and clinical illness was reported. As well, the cohort was composed almost entirely of males. Therefore, these results may not be applicable to the growing proportion of HIV positive patients who are female.

Use of an increased antigen dose to produce higher neutralizing antibody titres has been effective in other populations. A study of escalating doses of hemaglutinin antigen (4 to 61µg) in immune competent recipients demonstrated a correlation between antigen dose and higher HI antibody titre [48]. This strategy may also benefit immune compromised populations. A randomize, controlled study of double dose vaccination was conducted in a population of frail elderly subjects [49]. The first vaccine dose (15µg versus 30µg) was followed 84 days later by a second injection (placebo versus 15µg). Twenty-five days after the initial injection, the geometric mean titres in double dose recipients was 15% greater (95%CI, 6% to 24%, $p=0.001$) which supports the strategy of higher antigen dosing. The GMT in booster recipients was 14% (95%CI, 9% to 19%,

p=0.001) compared to placebo which supports the strategy of booster dosing. A short coming of this work was that clinical outcomes were not described.

High antigen dose was evaluated in a group of healthy volunteers vaccinated with an experimental H5N1 stain influenza vaccine [50]. Although the overall results were disappointing, there was a clear dose-response relationship between hemaglutinin antigen dosing and antibody titres (defined as ≥ 1:40) when assessed by HI assay and microneuralization testing (p<0.001). The use of 90µg of H5N1 influenza virus strain hemaglutinin antigen followed by a booster dose 28 days later produced higher titres in a greater proportion of randomized study participants after both the first and booster dose of vaccine. Twenty-eight percent, 23%, 10%, 5% and 0% of subjects developed HI titres ≥1:40 twenty-eight days after the first dose of antigen (90µg, 45µg, 15µg, 7.5µg, 0µg). Similar results were observed for microneutralization titres. After injection of a booster dose of vaccine, desired HI titres were achieved in 57%, 41%, 24%, 13% and 0% of recipients of 90µg, 45µg, 15µg, 7.5µg and 0µg antigen doses (p<0.001). This study further demonstrates the principal that higher antibody titres can be produced by use of higher antigen dosing and by booster dosing of vaccine.

1.1.6 Systematic Reviews

We conducted our own meta-analysis [51]. We searched 10 electronic databases independently, in duplicate (from inception to June 2007). We extracted data on study design, population characteristics and on outcomes related to influenza symptoms and HI titres. We pooled data using a random effects model and conducted sensitivity analyses to evaluate heterogeneity. Four studies meet inclusion criteria. Three studies were evaluable for meta-analysis and yielded a pooled Relative Risk Reduction [RRR] of 66% (95% Confidence Interval [CI] 36 to 82%, I^2=73%). One case-control study yielded an Odds Ratio of 1.98 (95% CI, 0.75 to 5.20. When we assessed heterogeneity according to study designs, studies of highest quality, only 1 RCT, yielded the most conservative estimate (RRR 41%, 95% CI, 2 to 64%). In conclusion, evidence supporting influenza vaccination within the HIV population is sparse and is characterized by major methodological shortcomings. A reasonable estimate of influenza vaccination effectiveness in HIV+ patients is lacking. As such, we believe that there is an urgent need for randomized trials to guide policy and clinical practice.

1.2 Rationale

Immune compromised individuals are at risk for infection with influenza and more likely to manifest more severe symptoms of influenza disease. Furthermore, they are influenza vaccine hyporesponsive in comparison to healthy, adult immune competent individuals. One population of immune compromised Canadians at risk for severe influenza disease is those living with HIV infection. At least 56,000 Canadians are HIV infected [1]. This population is at risk for more severe influenza illness. Influenza viral replication and shedding is prolonged and the duration of influenza symptomatology is longer in those with HIV [2, 3]. Furthermore, influenza-related mortality rates in HIV infected individuals are increased [4]. The HIV population is known to be

hyporesponsive to vaccinations, including influenza. The efficacy of influenza vaccines is compromised, in part, by reduced antibody responses observed in HIV infected individuals [5]. Nevertheless, influenza vaccination is recommended for HIV-infected individuals [6, 7]. The Centers of Disease Control guidelines state: “Influenza can result in serious illness and because vaccination with inactivated influenza vaccine might result in the production of protective antibody titers, vaccination might benefit HIV-infected persons. Therefore, influenza vaccination is recommended”. As influenza vaccination is the cornerstone of public health interventions intended to protect the population against influenza, vaccine hyporesponsiveness in immune compromised populations represents a significant concern. Given the risk of influenza exposure in general as well as concerns related to poor vaccine efficacy and more severe influenza disease in immune compromised populations such as those living with HIV, strategies to improve vaccine efficacy are required.

1.2.1 Why is a trial needed now?

A total of 5 conditions provide justification for a trial to be conducted at this time: (1) current standard treatment with influenza vaccine is less efficacious when used in particular subgroups of immune compromised individuals, such as those diagnosed with HIV; (2) there exists a significant burden of influenza infection in HIV patients that must be addressed in terms of identifying an effective treatment strategy; (3) past randomized trials of influenza vaccination in HIV patients are of limited comparability to today’s relevant base of patients, and alternative vaccination strategies require assessment; (4) efficacy of booster doses of influenza vaccine in HIV patients remains in question as a consequence of methodologic shortcomings in terms of both design aspects and outcomes measured of past studies; and (5) there is a paucity of published evidence assessing the efficacy of an increased, double-dose of influenza vaccine in this patient population.

1.3 Potential Risks and Benefits

1.3.1 Potential Risks and Discomforts:

The most common reactions to any vaccine include redness and swelling at the injection site pain, minor bruising, and hardness at the injection site are also possible. Another, but less common reaction to the influenza vaccine is to feel generally unwell with symptoms including muscle aches, fatigue, headache, and/or mild fever, shivering, and sweating for a day or two after getting the vaccine. There may be other side effects that are not anticipated at this time. As with any drug, there is a very small possibility that an allergic reaction to the vaccine may occur.

Some people experience discomfort, bruising and tenderness at the site where blood is taken. Fainting while blood is being taken or local infection at the puncture site may also occur, although these are rare.

The safety of Fluviral vaccine is established in pregnancy and breast feeding. As such, no special measures are required in this study.

In any research study there is always the possibility that harmful side effects which are not known at this time may occur.

1.3.2 Potential Benefits

The potential benefit from participating in this study is to provide immunity to one or more of the viruses contained in the 2008 Fluviral vaccine.

2. Study Objectives

2.1 Primary Objective

The primary goal is to assess the immunogenicity of three vaccination strategies using influenza vaccine in HIV infected adults.

2.2 Secondary Objectives

Secondary goals are to (1) assess the clinical efficacy in preventing flu-like illness, (2) assess the clinical efficacy in preventing laboratory-documented influenza.

3. Eligibility Criteria

3.1 Inclusion Criteria

Adults will be eligible for recruitment and participation in this study if they meet all of the following inclusion criteria:

- Age 18 - ≤ 60 years
- HIV positive
- Able to provide signed, informed consent.

3.2 Exclusion Criteria

Adults will be excluded from study participation if they meet any of the following criteria:

- Receipt or anticipated requirement of any blood product, vaccine, or immunoglobulin preparation within one month of study vaccine administration until completion of study.

- Immunosuppressive therapy including prednisone, immune modulators, subjects undergoing dialysis, autoimmune dysfunction (including rheumatoid arthritis, lupus erythematosus, multiple sclerosis)
- Alcohol consumption ≥ 4 drinks per day (1 drink is equal to a 12-ounce can of beer, or a 5-ounce glass of wine or one cocktail with 1 1/2-ounces alcohol)
- History of cancer, with the exception of cutaneous cancers including Kaposi Sarcoma, basal cell carcinoma and non-invasive HPV-related malignancy
- Known or suspected hypersensitivity to any component of the study vaccines, including chicken eggs or egg products and thimerosol
- History of immediate hypersensitivity reaction and/or reaction resulting in neurological symptoms to a previous dose of any influenza vaccine
- Presentation with or any recent history (within 24 hours) of any febrile illness (>38 C) or symptoms of significant local or systemic infection - such subjects will be deferred from enrollment at least until one week after the illness has resolved
- Any other condition which in the opinion of the Investigator might interfere with evaluation of the study objectives.

There will be no exclusion criteria for antiretroviral use.

4. Study Design

4.1 Description

This is a Phase III, randomized, multi-centered, controlled, vaccine study with three parallel groups. It will be conducted at Canadian HIV Clinical Trials Network sites. Approximately two hundred and eighty five (285) HIV infected volunteers, in otherwise good health, aged 18 - ≤ 60 years, will be recruited from the clinic patients at approximately 10 different Canadian HIV Clinical Trial Network sites.

4.2 Treatment Groups

Subjects will be randomized to one of three groups with 95 subjects per group.

Group A will receive one full dose of influenza vaccine (0.5 ml) between October 1st and November 15th 2008, followed by a booster influenza vaccine administered 28 days after the first, between November 1 and December 15 2008.

Group B will receive one double dose of influenza vaccine (1.0 ml) between October 1st and November 15th 2008, followed by a booster double dose of vaccine administered 28 days after the first between November 1 and December 15 2008.

Group C will receive an adult dose (0.5 ml) of influenza between October 1st and November 15th 2008, followed by a normal saline placebo injection (0.5 ml) administered 28 days after the first, between November 1 and December 15 2008.

Table 1 Vaccine Dosing Schedule

Study Groups: Cohorts and Vaccine Formulations

Group	Vaccine composition	
	Influenza Vaccine Dose[1]	
	Day 0	Day 28
A (n=95)	15 μg	15 μg
B (n=95)	30 μg	30 μg
C (n=95)	15 μg	0 μg

[1] The influenza vaccine dose pertains to the recommended adult dose. Therefore, a 1-times dose contains 0.5 ml of the influenza vaccine, comprising 15 μg of each of the 3 HA antigens.

Table 2 Visits and Procedure Schedule

Visit Number	1 (Screening[1])	2	3	4	5	Unscheduled / Event Related
Week Number	0 to - 12	0	4	8	20	
Window	0 to -90 days	-	28 days +/-3 days	56 days +/-7 days	140 days +/-7 days	
Inclusion/Exclusion	X	X				
Informed consent	X					
Medical history	X					
Baseline History[(6)]		X				
Physical Exam (Investigator discretion)	X	X	X	X	X	X
Vital signs [(2)]	X	X	X			X
Immunology [(3)] (HI and anti-HA)		X	X	X	X	
Immunology [(4)] (CMI)		X	X	X	X	
Vaccination		X	X			
Health Care Resource Utilization			X	X	X	X
Subject Diary[(5)]		X	X	X	X	X
Study Period Flu Symptoms Questionnaire						X
Nasopharyngeal Swab (PCR testing)						X
Adverse Events		X	X	X	X	X
Concomitant Medications		X	X	X	X	X
Health Utilities Index		X	X	X	X	X

[1] *Screening assessment may be performed same day as vaccination but will be completed prior to vaccination.*

[2] *Vital signs (oral temperature, heart rate, respiratory rate, blood pressure, height and weight) will be taken at screening. On vaccination days oral temperature only will be taken immediately prior to vaccination, which will be postponed at least 1 week in the event of fever. Oral temperature only will also be taken on unscheduled/event related visits.*

[3] *Anti-HA antibodies (ELISA) and HI assay (immunogenicity measures). Must be collected pre-vaccination.*

[4] *CMI: T-cell proliferation, cytokine secretion (immunogenicity measures) (Cooper/Klein sites only) Must be collected pre-vaccination*

[5] *Subjects will maintain a diary after vaccination during which they will record their vaccine reactions, oral temperature and any febrile respiratory tract symptoms as well as general changes to health and medications. The diary will be evaluated at each visit.*

[6]*Baseline history includes Employment Questionnaire, Substance Use and Previous Influenza Vaccine information.*

4.3 Randomization

During Visit 2, the research coordinator will telephone a centralized computer maintained at The Canadian HIV Trials Network. The system will generate a coded number that will be used to randomize participants. Once this coded number is obtained, the research coordinator will prepare the appropriate vaccine for injection

Randomization will be stratified by CD4 count (<200 cells/µL versus ≥200 cells/µL) and by site and blocked into variable blocks of 3.

4.4 Study Endpoints

4.4.1 Primary Endpoint

The primary outcome is the immunogenicity of the influenza vaccine. Blood samples to assess haemagglutination inhibition (HI), will be obtained at the following visits: baseline (prior to vaccination), week 4 (prior to booster vaccination / saline injection), week 8 and week 20.

The primary outcome is four-fold increase in HI titre at week 8; titre >40 at this time point will also be examined for those with titres <20 at baseline. These outcomes will in addition be assessed at week 12 and 20. Titres at week 4 will be compared between single dose (combined arms) and double dose. Titres >80 will also be examined for each of these outcome definitions.

4.4.2 Secondary Endpoints

The secondary endpoints are (1) self reported diaries of clinical signs and symptoms of respiratory and flu-like illness and (2) the PCR assays for influenza A and B taken from nasal swabs of subjects who present with a temperature > 38 ° C. These subjects will also have clinical evaluation of their respiratory and flu-like illness.

Please see Appendix B for a sample of the subject diary.

5. Expected Duration of Subject Participation

Subjects will be required to attend the clinic five (5) times between July 1 2008 and April 15 2009. The time between Visit 1 screening, and Visit 2 vaccination, will range from zero (0) to 90 days since vaccinations will begin October 1 2008. Please see the Study Visits [section 13] for the length of time between visits. Each visit will take approximately 45 minutes to 1 hour. If the subject's CD4 count is available from the clinic chart, the screening visit could be the same day as Visit 2.

6. Early Termination or Withdrawal

The Investigator will remove a subject from the study, either prior to or after vaccination, if the subject:

- experiences a serious or intolerable adverse event
- takes medications/vaccines that are contraindicated
- incurs a relevant protocol violation
- requests an early discontinuation due to a clinical event for which the Investigator did not consider removal from the study to be necessary
- other (non-specific) subject initiated cause

7. Participation of Women and Children:

7.1 Women

The safety of Fluviral vaccine is established in pregnancy and breast feeding. As such, no special measures are required in this study.

7.2 Children

Subjects younger than 18 years of age will be excluded from the study as there are insufficient data regarding dosing or adverse events available in adults to judge the potential risk in children.

8. Study Medication

8.1 Study Medication Description

The vaccine to be used is a trivalent killed split non-adjuvanted influenza vaccine. A 0.5 ml adult dose of recent versions of influenza contains 15 μg of haemagglutinin (HA) antigens for each of the three component strains: Influenza A (H1N1), one strain of Influenza A (H3N2), and one strain of Influenza B. Strains are selected yearly on recommendation of the World Health Organization. In each standard adult dose of vaccine (0.5ml, for intramuscular injection) there are 15µg of hemagglutinin from each of the 3 strains, trace amounts of thimerosol (0.01%) and egg protein [52]. Use of this

vaccine has been demonstrated to be safe and effective in a variety of healthy and immune compromised populations [53].

8.2 Potential Side Effects

Potential site effects may be found in the Product Monograph in Appendix F.

8.3 Formulation, Packaging, and Labeling

Fluviral is a marketed product, supplied in 5 mL vials holding 10 x 0.5 mL doses, and the formulation, packaging and labeling for this study will be as per the product Monograph. Please refer to Product Monograph in Appendix F.

8.4 Study Vaccine and Accountability Procedures

Fluviral should be stored in the refrigerator at +2 ° C to +8 ° C and should be shipped at the same temperature. Vaccine that has been frozen should not be used. Initial vaccine shipments will be shipped to sites from GlaxoSmithKline. Additional vaccine will be shipped to Dr. Cooper from GlaxoSmithKline and from Dr. Cooper to sites as needed. Overall accountability and dispensing logs will be kept by the site and will be verified by the monitor.

9. Concomitant Medications/Natural Remedies/Foods

The following concomitant medications are not allowed during the study:

- Receipt or anticipated requirement of any blood product, vaccine, or immunoglobulin preparation within one month of (prior to or after) study vaccine administration
- Immunosuppressive therapy including prednisone, immune modulators, subjects undergoing dialysis, autoimmune dysfunction (including rheumatoid arthritis, lupus erythematosus, multiple sclerosis)

There are no restrictions for herbal/alternative medications.

Please collect information about all concomitant medications / natural remedies / foods on the CRF's. (e.g. OTC medications, prescription medications, vitamins, herbal remedies, certain foods/juices, etc.).

10. Concomitant Alcohol (Please see exclusion criteria)

Alcohol consumption greater than or equal to 4 drinks per day is not allowed during study. For example: 1 drink is defined as a 12-ounce can of beer, or a 5-ounce glass of wine or one cocktail with 1 1/2-ounces alcohol per day is the equivalent of 50 grams per day.

11. Prohibited Medications and Procedures

The following medications/procedures are not allowed during study.

- Receipt or anticipated requirement of any blood product, vaccine, or immunoglobulin preparation within one month of (prior to or after) study vaccine administration
- Immunosuppressive therapy including prednisone, immune modulators, subjects undergoing dialysis, autoimmune dysfunction (including rheumatoid arthritis, lupus erythematosus, multiple sclerosis)

12. Study Evaluations/Procedures

12.1 Clinical Evaluations

12.1.1 General Clinical Evaluation

Research staff will determine that subjects have the diagnosis of HIV and are in otherwise good health by medical history, routine vital signs and physical examination as required.

12.1.2 Clinical Evaluation for Influenza Infection

All subjects developing febrile respiratory syndromes during the 20-week period following initial influenza vaccination, will be asked to report to clinic for assessment for influenza infection.

At enrolment subjects will receive a thermometer, ruler, transparency measuring tool and respiratory illness symptom diary and be requested to contact the research coordinator at the onset of any respiratory illness so that prompt assessment can be arranged.

Respiratory infections will be defined as a temperature >38.0° C associated with any one or more of the following clinical symptoms: feverishness/chills; cough; tachypnea/dyspnea; wheezing/stridor; rhinorrhea; sore throat; myalgias.

- Pertinent clinical data will be collected during consultation using a standardized questionnaire (Please see Appendix C)
- Information related to the duration and nature of symptoms and history of infectious contacts will be gathered.
- The patient will be asked to record the signs and symptoms of respiratory illness daily until resolution.
- For study purposes, a nasopharyngeal swab will be collected for influenza identification.

- Additional investigations such as chest radiographs, laboratory work-up and rapid influenza testing will be performed at the discretion of the treating physician but should be documented on the appropriate Study Case Report Form.
- Prescription and use of medications for management of the respiratory illness, including oral influenza medication, will be recorded.
- Hospitalizations will be recorded.

12.2 Laboratory Evaluations and Specimen Collection

12.2.1 Immunogenicity Laboratory Measurements

12.2.1.1 Baseline Visit Serum Samples (Immunology)

Blood samples will be obtained at the baseline visit (prior to vaccination) to assess haemagglutination inhibition (HI) and anti-HA levels against any of the three specific serotypes selected for the 2008 influenza seasons.

Blood samples will be centrifuged and the sera from each will be aliquoted into four vials (minimum 2.0 ml/vial) for storage at the laboratory. All specimens will be labeled and kept frozen (at -80°C) at the laboratory facility.

Three sets of aliquots of each serum sample will be transported frozen to the laboratory of Dr Guy Boivin (University of Laval) for evaluation of HI assay and anti-HA ELISA testing. The fourth set of samples will remain in a freezer at the laboratory facility in case the others are rendered unsuitable for testing.

12.2.1.2 Post-vaccination Immunology Serum Samples

Blood samples will be obtained at week 4 (+/- 3 days, prior to booster vaccination), week 8 (+/- 7 days in consideration of Christmas break), and week 20 (+/- 7 days) after vaccination to assess serological responses to the 3 vaccine strategies.

Blood samples will be centrifuged and the sera from each will be aliquoted into four vials (minimum 2.0 ml/vial) for storage at the laboratory. All specimens will be labeled and kept frozen (at -80°C) at the laboratory facility.

Three sets of aliquots of each serum sample will be transported frozen to the laboratory of Dr Guy Boivin (University of Laval) for evaluation of HI assay and anti-HA ELISA testing. The fourth set of samples will remain in a freezer at the laboratory facility in case the others are rendered unsuitable for testing.

12.2.2 Whole Blood Samples for Evaluation of Cell Mediated Immunity (CMI, Immunogenicity)

To assess cell-mediated immune responses, whole blood will be collected from subjects on the day of vaccination and 4, 8 and 20 weeks thereafter at 2 sites (Cooper-Ottawa,

Klein- Montreal). Blood samples will be drawn prior to vaccination on vaccination/booster vaccination visits.

Peripheral blood mononuclear cells (PBMC) will be isolated by Ficoll-Paque and then immediately frozen at -80 ° C for 24-48 hrs and then transferred at -150 ° C. The cells will be subsequently used for *in vitro* stimulation with HA antigens (3 serotypes) and assay of cytokine secretion and proliferative responses.

12.2.3 Nasopharyngeal Swab for Laboratory Assessment of Respiratory Illness

If a subject becomes febrile and has symptoms of a respiratory infection, a nasopharyngeal swab will be collected to determine if influenza and other respiratory tract viruses are present.

Nasal swabs will be performed by inserting a sterile calcium alginate tipped culture swab into the anterior nares and gently rubbing to remove some epithelial cells. Similarly, the posterior pharynx will be swabbed using a second sterile swab. The two swabs will then combined into a single 1.5 ml vial of viral transport medium composed of Hank's Balanced Salt Solution (Biowhittaker, Walkersville, MD) with 5% fetal calf serum (Wisent Inc., St-Bruno, QC, Canada) buffered with 2% 2.5M HEPES (Fischer Scientific, Ottawa, ON, Canada) and supplemented with amphotericin B (500 ug/ml; Bristol Myers Squibb, Montreal, QC, Canada), gentamicin (1600 ug/ml; Sandoz, Boucherville, QC, Canada), penicillin G (20,000 U/ml; Pharmaceutical Partners Canada, Manitok, ON, Canada) and 0.002% 1N HCl. The viral media and respiratory samples will be stored at –80 °C until PCR analyses. All specimens will be batch transported to the laboratory of Dr. Guy Boivin (University of Laval) at the conclusion of the study.

All respiratory samples will be tested for the presence of a panel of 4 respiratory viruses using an in-house real-time multiplex reverse-transcriptase PCR assay as described elsewhere [10, 11]. [Boivin G, De Serres G, Cote S, et al. Human metapneumovirus infections in hospitalized children. Emerg Infect Dis 2003; 9:634-40 ; Hamelin ME, Abed Y and Boivin G. Human metapneumovirus: a new player among respiratory viruses. Clin Infect Dis 2004; 38:983-90.]. This assay includes 4 sets of primers for detection of influenza A (matrix) and B (matrix), RSV (fusion), and hMPV (nucleoprotein) in a LightCycler instrument (Roche Diagnostics, Laval, QC, Canada). The different viruses (limit of detection 50-100 copies/assay) were differentiated based on their specific melting temperatures as calculated by the LightCycler instrument.

12.3 Stored Research Specimens

12.3.1 Use of Stored Specimens

Laboratory specimen collection, testing and storage is detailed in *Section 12.2 above.*

Genetic testing will not be performed on specimens collected for this study.

Laboratory specimens will not be used for any other research protocols.

12.3.2 Disposition of Stored Specimens

Only investigators will have access to the specimens and data. Subjects may decide at any point not to have their specimens stored. In this case, the principal investigator will destroy all known remaining specimens and report what was done to both the subject and to the IRB/REB. This decision will not affect the subject's participation in this protocol or any other protocols at CTN.

Stored specimens not required, will be removed from frozen storage thereby rendering them unusable. The specimens will then be destroyed as hazardous waste.

12.4 Questionnaires / Diaries

The following questionnaires will be utilized during the study:

- Health Utilities Index (HUI 2/3): Observer-administered questionnaire assessing Health-Related Quality of Life (HRQoL) at baseline and all follow-up visits. The HUI 2/3 scoring systems provide preference-weighted measures of health status, or measures of health utility, which will be used in the economic evaluation. (Please refer to Appendix D.)
- Employment Questionnaire: Observer-administered questionnaire assessing work status and wages at baseline. (Please refer to Appendix D.)
- Health Care Resource Utilization Questionnaire: Observer-administered questionnaire assessing inpatient and outpatient care at each follow-up interval, as well as days of work missed. (Please refer to Appendix D.)
- Subject Diary: The subject diary will be completed by subject after vaccination (until the end of the study) in order to collect any symptoms of respiratory illness as well as any reactions to the vaccine and general health and medication changes. (Please refer to Appendix B.)
- Study Period Flu Symptom Questionnaire: The Study Period Flu Symptom Questionnaire, observer-administered, will be administered when to subjects that present with flu-like illness for an event-related visit in order to confirm the illness. (Please refer to Appendix C.)

13. Study Visits

13.1 Screening (Visit 1)

Visit 1, the screening visit should occur no more than 90 days before Visit 2.

The screening visit will take approximately 1 hour and include the following procedures:

- Assessment of Inclusion/Exclusion Criteria
- Obtain written acceptance of informed consent
- Medical History
- Physical exam (targeted or full) as necessary
- Obtain vital signs
- *CD4 count (as drawn per standard of care) will be obtained from the subject's clinic chart and must be available before Visit 2.*
- *Viral Load (most recent as drawn per standard of care)*

13.2 Randomization and Vaccination (Visit 2, Week 0)

Visit 2 should take approximately 1 hour.

The research coordinator will follow the randomization procedures described in section *4.3* above the following procedures will be performed:

- Confirm all inclusion and exclusion criteria continue to apply
- Obtain vital signs
- Physical exam (targeted or full) as necessary
- Blood samples to assess haemagglutination inhibition (HI) and anti-HA levels.
- Blood samples to assess CMI (Cooper/Klein sites only)
- Vaccination
    - Subjects will be observed for 15 minutes post vaccination as per standard of care. Resuscitation equipment is required to be rapidly assessable in the event of an anaphylactic event.
- Subject Diary:
    - Review in detail with the subject the signs, symptoms, and other details to be recorded and when.
    - Diaries should be completed by the subject only
    - Provide the details and contact information for the researcher to be contacted with questions about the diary and when the subject has a temperature > 38 °C .
- Provide subject with thermometer and instructions for its use.
- Provide subject with ruler and transparency tool and instructions for use
- Assessment of post vaccination Adverse Events
- Review of Concomitant Medications
- Schedule next visit 28 days (+/-3 days)

13.3 Booster Vaccination Day (Visit 3, Week 4)

This visit should take approximately 1 hour.

- Confirm all inclusion and exclusion criteria continue to apply
- Review AE's
- Review Concomitant Medications

- Review subject's diary
- Obtain vital signs
- Physical exam (targeted or full) as necessary
- Blood samples to assess haemagglutination inhibition (HI) and anti-HA levels.
- Blood samples to assess CMI (Cooper/Klein sites only)
- *Vaccination*
    - Subjects will be observed for 15 minutes post vaccination as per standard of care. Resuscitation equipment is required to be rapidly assessable in the event of an anaphylactic event.
- Review Subject Diary instructions and provide with new card if necessary
- Assessment of post vaccination Adverse Events
- Schedule next visit 28 days (+/- 7 days)

13.4 Post Vaccination Visits:

All Groups (Visits 4 and 5, Weeks 8 and 20)

Each post vaccination visit should take approximately 45 minutes.

The following procedures will be performed at the post vaccination visits:

- Review AE's
- Review Concomitant Medications
- Review subject's diary
- Obtain vital signs
- Physical exam (targeted or full) as necessary
- Obtain blood samples to assess haemagglutination inhibition (HI) and anti-HA levels.
- Blood samples to assess CMI (Cooper/Klein sites only)
- Review Respiratory Symptom Diary instructions and provide with new card if necessary
- Schedule next visit (+/- 7 days) if applicable.

13.5 Unscheduled Visit Due to Respiratory Symptoms

- Participants will document fever and symptoms in the study diary.
- They will contact the research coordinator to arrange for an 'unscheduled' study visit if they have a fever (temperature > 38 °C and 1 additional symptom.
- A history of present medical illness will be entered in the CRF. Vital signs (temperature) will be recorded.
- Physical exam findings will be recorded as applicable.
- A nasopharyngeal swab will be collected and then placed in frozen storage.
- If further standard of care evaluation is required by the physician, a clinic evaluation will be arranged.

- Information from this standard of care evaluation (i.e. CXR findings, microbiology / virology results, hematology and chemistry blood work) will be collected on the CRF.

13.6 Early Termination Visit

If the subject discontinues from study but agrees to return for a termination visit, follow the procedures for Visit 5.

If the subject has received at least one dose of vaccine and is willing to continue visit assessments without additional vaccine then continue with visits as scheduled, without the additional vaccine injection.

14. Assessment of Safety

14.1 Safety Monitoring

The Safety and Efficacy Review Board (SERC) will be responsible for the Safety Monitoring in this trial. (Refer to Section 16.3 for more details).

SERC will review data in real time after dosing and will convene ad hoc when necessary via teleconference to discuss any safety issues that arise.

14.2 Adverse Events (AE's)

An AE is any untoward medical occurrence in a patient or clinical investigation participant, administered a Study Medication/Intervention, which does not necessarily have a causal relationship with this treatment. An AE can therefore be any unfavorable and unintended sign (including an abnormal laboratory finding), symptom, or disease temporally associated with the use of a medicinal (investigational) Study Medication/Intervention, whether or not related to the medicinal (investigational) Study Medication/Intervention.

Grade 1 and Grade 2 events as defined by 'CTN Toxicity Table for Grading Adverse Experiences' are not considered adverse events, but details of these events must be documented in detail in the subject's study files. (Refer to Section 14.6.1 AE/SAE Grading and Appendix G for guidelines on determining Grade of event.)

Stable chronic conditions which are present prior to clinical trial entry and do not worsen are not considered adverse events and will be accounted for in the subject's medical history.

14.3 Local/Systemic Reactogenicity

Subjects will be asked to document specific local and systemic reactions in a diary for 1 week (7 days) following each injection. A transparency showing sizes of circles and a ruler will be provided to subjects as a tool for assessing reactions visually. (Refer to Appendix B: Patient Diary for more details.)

Solicited reactogenicity AEs will be assumed to be related to the test article. Otherwise, the relationship of the study treatment to an AE will be determined by the investigator based on the relationship assessment in section 14.6.2.

14.4 Recording/Documentation of AE's

At each contact with the subject, information regarding adverse events will be elicited by appropriate questioning and examinations and will be recorded immediately on a source document. (e.g. progress notes, laboratory reports, consult notes, phone logs, survey tools and data collection tools.) All reportable adverse events that are identified will be recorded on the appropriate case report form (CRF). The start date, the stop date, the severity of each reportable event, and the PI's judgment of the AE's relationship to the study medication/intervention will also be recorded on the CRF.

Any AE that occurs between the time a subject is randomized (visit 2, week 0) and the time she/he departs the study at the end of the final follow-up visit (visit 5, week 20 or at the time of early discontinuation of the subject from the study for any reason) is to be recorded.

14.5 Serious Adverse Events *(SAE's)*

A Serious Adverse Event is defined as an AE meeting one of the following:

- Death during the period of protocol-defined surveillance
- Life Threatening Event (defined as a participant at immediate risk of death at the time of the event)
- In-patient hospitalization or prolongation of existing hospitalization during the period of protocol-defined surveillance
- Results in congenital anomaly or birth defect
- Results in a persistent or significant disability/incapacity

Any other important medical event that may not result in one of the above outcomes, may be considered a serious adverse experience when, based upon appropriate medical judgment, the event may jeopardize the participant and may require medical or surgical intervention to prevent one of the outcomes listed above. Examples of such medical events include allergic bronchospasm requiring intensive treatment in an emergency room or at home, blood dyscrasias or convulsions that do not result in in-patient hospitalization, or the development of drug dependency or drug abuse.

If an SAE is ongoing at the time a subject discontinues/completes the trial the SAE will be followed up until appropriate medical care for the SAE can be established.

14.6 AE/SAE Grading and Relationship Assignment

14.6.1 AE/SAE Grading

The Intensity (severity) for each adverse event/serious adverse event will be graded according to the toxicity table and toxicity guidelines shown in Appendix G: CTN Toxicity Table for Grading Adverse Experiences.

14.6.2 Relationship Assessment

For all collected AE's/SAE's, the clinician who examines and evaluates the subject will determine the adverse event's causality based on temporal relationship and his/her clinical judgment. The degree of certainty about causality will be graded using the categories below:

Definitely Related: There is clear evidence to suggest a causal relationship, and other possible contributing factors can be ruled out. The clinical event, including an abnormal laboratory test result, occurs in a plausible time relationship to drug administration and cannot be explained by concurrent disease or other drugs or chemicals. The response to withdrawal of the drug (de-challenge) should be clinically plausible. The event must be pharmacologically or phenomenologically definitive, with use of a satisfactory re-challenge procedure if necessary.

Probably Related: There is evidence to suggest a causal relationship, and the influence of other factors is unlikely. The clinical event, including an abnormal laboratory test result, occurs within a reasonable time sequence to administration of the drug, is unlikely to be attributed to concurrent disease or other drugs or chemicals, and follows a clinically reasonable response on withdrawal (de-challenge). Re-challenge information is not required to fulfill this definition.

Possibly Related: There is some evidence to suggest a causal relationship (e.g., the event occurred within a reasonable time after administration of the trial medication). However, the influence of other factors may have contributed to the event (e.g., the subject's clinical condition, other concomitant events). Although an adverse drug event may rate only as "possibly related" soon after discovery, it can be flagged as requiring more information and later be upgraded to "probably related" or "definitely related", as appropriate.

Unlikely: A clinical event, including an abnormal laboratory test result, whose temporal relationship to drug administration makes a causal relationship improbable (e.g., the event did not occur within a reasonable time after administration of the trial medication) and in which other drugs or chemicals or underlying disease provides plausible explanations (e.g., the subject's clinical condition, other concomitant treatments).

Not related: The AE is completely independent of study drug administration, and/or evidence exists that the event is definitely related to another etiology. There must be an alternative, definitive etiology documented by the clinician.

14.7 SAE Reporting Procedures

All SAE's which occur during the course of the study must be reported to the CTN by phone and fax within 24 hours of the site becoming aware of the event.

Serious adverse events will be reported to:

Canadian HIV Trials Network
Attention: Wendy Zubyk
Phone: 604-806-8526
Fax: 604-806-8005
E-mail: wzubyk@sm.hivnet.ubc.ca

14.8 Monitoring of AE's/SAE's

Any AE that occurs between the time a study participant is randomized (visit 2, week 0) and the time she/he departs the study at the end of the final follow-up visit (visit 5, week 20 or at the time of early discontinuation of the subject from the study for any reason) will be captured and recorded. At each contact with the subject, the investigator (or designate) must seek information on adverse events by specific questioning and, as appropriate, by examination.

Adverse events that had previously been reported by the study subject will also be reassessed for duration, intensity and possible reoccurrence.

All AE's and SAE's will be followed until appropriate medical care for the AE/SAE can be established.

15. Trial Management and Analysis

The Canadian HIV Trials Network will be responsible for project management, case report form development, site management, randomization, data collection and management, and data analysis.

Dr. Curtis Cooper will be responsible for acquisition and distribution of vaccine and supplies as well as laboratory related activities.

16. Statistical Considerations

16.1 Randomization and Blinding Procedures

A randomization list for each site and stratum will be generated by a statistician at the national data centre in Vancouver using a randomization program. These allocations will be entered onto a computer dedicated to the randomization of study patients. Site coordinators will be given authorization codes, which allow entry into the system via telephone to the site coordinators. The computer will record caller identifiers, non-nominal patient identifiers, time of call, and allocation and study identification number issued. Once this coded number is obtained, the research coordinator will prepare the appropriate vaccine for injection. Access to this information is restricted to the programmer maintaining the randomization computer.

Randomization will be stratified by CD4 count (<200 cells/μL versus ≥200 cells/μL) and by site and blocked into variable blocks of 3.

16.2 Sample Size Considerations/Justification

Sample size calculations for this study were estimated based on the formula for comparison of two independent proportions using a two-tailed α of 0.05 and a (1-β) of 0.90. The control rate of doubling of titres was estimated to be 50%, as indicated by results of a previously undertaken study [54]. We hypothesize that the modified doses of influenza will improve the proportion of those doubling titre levels by 25%, to a rate of 75%. This would be considered a clinically relevant increase in HI titres. While we believe that the earlier described influenza regimens in the two experimental groups are superior to that provided to the control group in this patient population, we cannot rule out that it may be inferior. While this is considered a remote possibility, we have conservatively assumed a two-sided α. To account for possible randomization failures, a conservative 10% non-compliance factor was incorporated using the method described by Lachin. Based on these assumptions, a total of 95 patients per arm is required. Given that the same minimal important difference is common for both experimental arms of the study, a total sample size of 3 x 95 = 285 patients is required. This sample size would also provide 80% power for comparison of the secondary outcome between groups, where lab confirmed influenza rates of 30% in both experimental groups and 50% in the control group are hypothesized.

16.3 Role of the Data and Safety Monitoring Board

The Canadian HIV Trials Network (CTN) has a standing Data and Safety Monitoring Board (DSMB), which monitors CTN trials which will be used for this study: The Safety and Efficacy Review Committee (SERC). The committee is composed of three clinicians, three methodologists, and one lay member. Face to face meetings occur every 6 months with ad hoc meetings, usually by teleconference, as required.

The DSMB reviews recruitment, safety, interim analyses of efficacy when necessary, adherence to protocol including withdrawals from assigned treatment and loss to follow-up and any new external information which may impact upon the study. The committee may recommend stopping of a trial based on any of these considerations. Recommendations to stop a trial are communicated to the trial investigator who has the right to appeal.

17. Details of Statistical Analysis

17.1 Statistical Analysis

Baseline characteristics of patients in the treatment groups will be analyzed with frequency distributions and descriptive statistics, including measures of central tendency and dispersion.

An intent-to-treat approach will be used, and therefore all analyses will be conducted using the entire cohort of patients.

17.2 Primary Endpoint

The principal analysis of our primary outcome will be done using an unadjusted Chi-Square test comparing the proportion of individuals achieving a doubling of titers in each treatment group. Further analysis will use logistic regression procedures to examine the effect of adjustment for clinically relevant covariates that are either imbalanced at baseline or strong predictors of the outcome. Additional individual variables and interactions will be considered based on clinical importance and empirical data. Additional "compliers only" and "as-treated" analyses will be conducted to examine the robustness of our primary estimates.

One injection vaccine (all doses) vs. booster dose (all doses) will be analyzed; restricted to those who received injections in appropriate dose and timing (i.e. protocol violators will be excluded.

17.3 Secondary Endpoints

Our secondary outcomes of interest, the frequencies of (1) laboratory confirmed influenza and (2) clinical/respiratory illness (as documented according to criteria detailed in section 12.1.2 in each group, will be compared using unadjusted Chi-Square tests, as well as logistic regression to adjust for important prognostic risk factors. The frequency of individual, clinically important criteria used to define clinical illness will also be assessed for both groups.

To supplement the study's primary analyses pertaining to titer changes, the humoral (HI activity) and cell-mediated (T-cell proliferation, key Th1 and Th2 cytokines) values for each of the three serotypes (both absolute values as well as changes from baseline)

obtained with each experimental or control vaccine will be summarized using means and 95% CIs for subjects receiving the influenza vaccines for each assessment time. The apparent immunogenicity will be described using a comparison of these 95% CI between the two booster strategies and control group. Assessment times are scheduled as follows:

- HI activity at baseline, 4, 8, 20 weeks;
- T-cell proliferation activity at baseline, 4, 8, 20 weeks;
- Key Th1 and Th2 cytokines at baseline, 4, 8, 20 weeks.

17.4 Other Variables of Interest

The following are variables of interest:

- CD4
- HIV viral load

Note: Additional blood work not to be performed unless it is done as per standard of care. Blood drawn on the visit only to be used in determination of the above if drawn prior to vaccine injection (i.e. drawn as 'pre-visit' blood work) does not have to be done unless part of standard of practice at the site)

17.5 Planned Subgroup Analyses

The following subgroup analyses are planned:

- sensitivity analysis based on CD4 cell count (CD4 count ><200)
- use of HAART, viral load suppression (><50 copies / mL)
- co-infection with HCV/HBV
- nadir CD4 count (to be captured in screening visit CRF)
- smoking status
- weight
- alcohol use
- influenza vaccination in previous year
- race
- gender

17.6 Other Analytical Issues

Missing HI titres (absolute, GMT and seroprotection (defined as >=40, >=80) will not be imputed.

Higher than expected loss to follow-up will be addressed using sensitivity analysis assuming none achieved seroprotection and assuming all did.

Acceptable windows around visits for inclusion of data are defined in section 13.

18. Quality Control and Quality Assurance

Each participating site will be monitored to verify that the clinical trial is conducted and data are generated, documented (recorded), and reported in compliance with the protocol, GCP, and the applicable regulatory requirements.

Quality control procedures will be implemented beginning with the data entry system and data quality control checks that will be run on the database will be generated. Any missing data or data anomalies will be communicated to the site(s) for clarification/resolution.

19. Ethics/Protection of Human Subjects

19.1 ICH Guidelines/Declaration of Helsinki

The conduct of this study will conform and with the International Conference for Harmonization Good Clinical Practice (ICH-GCP) regulations and guidelines and the current revision of the Declaration of Helsinki. (See appendices.)

19.2 Research Ethics Board/Institutional Review Board

A copy of the protocol (including protocol amendments), all versions of informed consent forms, other information to be completed by participants such as survey instruments or questionnaires, and any proposed advertising/ recruitment materials must be reviewed and approved by the REB/IRB of each participating centre prior to implementation of the trial. The investigator will be responsible for obtaining REB/IRB approval of the annual Continuing Review throughout the duration of the study. The investigator will notify the REB/IRB of violations from the protocol and serious adverse events.

19.3 Informed Consent Process

All participants will be given detailed oral and written information about the trial. Consent forms describing in detail the study medication, study procedures and risks will be given to each participant and written documentation of informed consent is required prior to starting study procedure.

Participants must sign an informed consent document that has been approved by a participating centre's REB/IRB prior to any procedures being done specifically for the trial.

Each participant should have sufficient opportunity to discuss the study and consider the information in the consent process prior to agreeing to participate. Participants may withdraw consent at any time during the course of the trial.

The informed consent form will be signed and dated by the participant and the person who conducted the informed consent discussion and obtained the consent. The original signed informed consent form will be retained in the participant's study files and a copy will be provided to the participant.

20. Participant Confidentiality

All subject related information including Case Report Forms, laboratory specimens, evaluation forms, reports, etc. will be kept strictly confidential. All records will be kept in a secure, locked location and only research staff will have access to the records. Subjects will be identified only by means of a coded number specific to each subject. All computerized databases will identify subjects by numeric codes only, and will be password protected.

Upon request, subject records will be made available to the study sponsor, monitoring groups representative of the study sponsor, representatives of a participating pharmaceutical sponsor and applicable regulatory agencies such as Health Canada Therapuetic Products Directorate or the USA Food and Drug Administration.

21. Early Termination of the Protocol

The sponsor will closely monitor and analyze study data as it becomes available and will make determinations regarding the presence and grading of adverse events. Evaluation of adverse events will be analyzed separately for study vaccine and placebo and with regard to the known complications associated with placebo administration. If/when appropriate, the study will be stopped [e.g. no new enrollments and no further administration of medication(s)/intervention(s)] by the investigators and a report will be submitted to the REB/IRB.

The REB/IRB, Health Canada, the pharmaceutical supporter(s), or other government agencies, as part of their duties to ensure that research subjects are protected, may discontinue the study at any time. Subsequent review of serious, unexpected and related adverse events by the Medical Monitor, DSMB, ethics review committee or REB/IRB, the sponsor(s), the Health Canada, and other regulatory authorities may also result in suspension of further trial interventions/administration of study medication at a site. Health Canada, other regulatory authorities, and the study sponsor(s) retain the authority to suspend additional enrollment and Study vaccine administration for the entire study as applicable.

22. Treatment Discontinuation

Complete the Visit 5 procedures if the investigator withdraws the subject, or if the subject withdraws from the study but agrees to a final visit.

The criteria for permanent discontinuation of further study product/interventions for an individual subject are as follows:

- Requirement for prohibited concomitant medications
- Completion of treatment/intervention as defined by the protocol
- Clinical reasons believed to be life-threatening by the physician, even if not addressed in the toxicity section of the protocol

The subject will continue to be followed with the subject's permission if the study treatment/intervention is discontinued. There will be no changes to the follow-up visit schedule, except no study treatment/intervention will be administered.

23. Premature Study Discontinuation for an Individual Subject

The criteria for permanent discontinuation from the study for an individual subject are as follows:

- Loss to follow-up
- Request of the subject to withdraw from the trial
- Any clinical adverse event, laboratory abnormality, intercurrent illness, other medical condition or situation occurs such that continued participation in the study would not be in the best interest of the participant.
- The subject is judged by the investigator to be at significant risk of failing to comply with the provisions of the protocol as to cause harm to self or seriously interfere with the validity of the trial results.

In the event that the subject is withdrawn from the study due to an AE, this must be recorded on the CRF. The subject should be followed and treated by the investigator until the abnormal parameter or symptom has resolved or stabilized. It is up to the clinician to determine that the AE is either resolved or that it has reached a stable state, after which no further follow-up is necessary. There should also be source documentation to support this determination.

24. Protocol Violations/Deviations

Requested deviations will be considered case by case.

Protocol violation/deviation related to vaccination dosing will be recorded. Participants will not be discontinued.

Follow-up vaccine dosing will be administered according to protocol.

Primary analysis will be by intent-to-treat.

Violation/deviation related to the timing of visit blood work will be documented. Participants will not be excluded.

25. Study Conduct and Monitoring

Each study site agrees to allow monitors from CTN and/or their representatives direct access to the study records and medical records from those patients enrolled in the clinical study as well as drug accountability records. Adequate monitoring space and time must be provided for the Clinical Research Associates. CTN and/or their representatives will perform ongoing study site monitoring to ensure quality assurance. Protocol violations will be monitored and recorded. Patient accrual and eligibility will also be monitored and recorded.

26. Data Management Responsibilities

Instructions concerning the recording of study data on case report forms will be provided by the Canadian HIV Trials Network Data Management Centre. Each study site is responsible for submitting the data in a timely fashion.

It is the responsibility of the Canadian HIV Trials Network Data Management Centre to assure the quality of computerized data for this study. This role extends from protocol development to generation of the final study databases.

27. Source Documents and Access to Source Data/Documents

Each participating site must maintain appropriate medical and research records for this trial and regulatory/institutional requirements for the protection of confidentiality of study subjects. The Principal Investigator is responsible for assuring that the data collected are complete, accurate, and recorded in a timely manner.

Study data will be collected on study specific case report forms (CRF's). Source documentation should support the data collected on the CRF's. Source documents include all recordings of observations or notations of clinical activities and all reports and records necessary for the evaluation and reconstruction of the clinical trial. Data for CRF's will be collected during patient visits, phone calls with subjects and health care providers, patient diaries, and completed questionnaires and abstracted from the subject's medical records. It is not acceptable for the CRF to be the only record of a subject's participation in the trial.

28. Disclosure and Publication Policy

Publication of the primary analysis, economic subanalysis, and screening subanalysis are planned. An additional manuscript focused on specialized influenza immunology testing will be pursued.

Dr Cooper will determine authorship on each manuscript based on contribution to the study design, execution and manuscript preparation. Dr Cooper will invite individual co-investigators and collaborators to assist in manuscript preparation. No author will be included without prior authorization.

29. REFERENCES

1. Boulos, D., et al., *Estimates of HIV prevalence and incidence in Canada, 2005.* Can Commun Dis Rep, 2006. **32**(15): p. 165-74.
2. Safrin, S., J.D. Rush, and J. Mills, *Influenza in patients with human immunodeficiency virus infection.* Chest, 1990. **98**(1): p. 33-7.
3. Radwan, H.M., et al., *Influenza in human immunodeficiency virus-infected patients during the 1997-1998 influenza season.* Clin Infect Dis, 2000. **31**(2): p. 604-6.
4. Zanetti, A.R., et al., *Safety and immunogenicity of influenza vaccination in individuals infected with HIV.* Vaccine, 2002. **20 Suppl 5**: p. B29-32.
5. Malaspina, A., et al., *Compromised B cell responses to influenza vaccination in HIV-infected individuals.* J Infect Dis, 2005. **191**(9): p. 1442-50.
6. *Health Canada Progress towards Canadian target coverage rates in Influenza and Pneumococcal Immunications.*, in *Available at: http://www.phac-aspc.gc.ca/publicat/ccdr-rmtc/01vol27/dr2710eb.htlm. Accessed 8 December 2006.* 2006.
7. *Prevention and Control of Influenza. Recommendations of the advisory committee on immunization practice*, in *Centers for Disease Control and Prevention. Morbidity and Morality Weekly Report.* 2006. p. Vol 55/RR-10.
8. Palache, A.M., *Influenza vaccines. A reappraisal of their use.* Drugs, 1997. **54**(6): p. 841-56.
9. Meiklejohn, G., et al., *Antigenic drift and efficacy of influenza virus vaccines, 1976--1977.* J Infect Dis, 1978. **138**(5): p. 618-24.
10. Ruben, F.L., *Prevention and control of influenza. Role of vaccine.* Am J Med, 1987. **82**(6A): p. 31-4.
11. Hirota, Y., et al., *Antibody efficacy as a keen index to evaluate influenza vaccine effectiveness.* Vaccine, 1997. **15**(9): p. 962-7.
12. Bridges, C.B., et al., *Prevention and control of influenza. Recommendations of the Advisory Committee on Immunization Practices (ACIP).* MMWR Recomm Rep, 2001. **50**(RR-4): p. 1-44.
13. Meiklejohn, G., *Viral respiratory disease at Lowry Air Force Base in Denver, 1952-1982.* J Infect Dis, 1983. **148**(5): p. 775-84.
14. Sugaya, N., et al., *Efficacy of inactivated vaccine in preventing antigenically drifted influenza type A and well-matched type B.* JAMA, 1994. **272**(14): p. 1122-6.

15. Barker, W.H. and J.P. Mullooly, *Influenza vaccination of elderly persons. Reduction in pneumonia and influenza hospitalizations and deaths.* JAMA, 1980. **244**(22): p. 2547-9.
16. Ruben, F.L., F. Johnston, and E.J. Streiff, *Influenza in a partially immunized aged population. Effectiveness of killed Hong Kong vaccine against infection with the England strain.* JAMA, 1974. **230**(6): p. 863-6.
17. Govaert, T.M., et al., *The efficacy of influenza vaccination in elderly individuals. A randomized double-blind placebo-controlled trial.* JAMA, 1994. **272**(21): p. 1661-5.
18. Gross, P.A., et al., *The efficacy of influenza vaccine in elderly persons. A meta-analysis and review of the literature.* Ann Intern Med, 1995. **123**(7): p. 518-27.
19. Blumberg, E.A., et al., *The immunogenicity of influenza virus vaccine in solid organ transplant recipients.* Clin Infect Dis, 1996. **22**(2): p. 295-302.
20. Dorrell, L., et al., *Clinical and serological responses to an inactivated influenza vaccine in adults with HIV infection, diabetes, obstructive airways disease, elderly adults and healthy volunteers.* Int J STD AIDS, 1997. **8**(12): p. 776-9.
21. Buxton, J.A., et al., *Influenza revaccination of elderly travelers: antibody response to single influenza vaccination and revaccination at 12 weeks.* J Infect Dis, 2001. **184**(2): p. 188-91.
22. Murphy, B.R. and M.L. Clements, *The systemic and mucosal immune response of humans to influenza A virus.* Curr Top Microbiol Immunol, 1989. **146**: p. 107-16.
23. Clements, M.L., et al., *Serum and nasal wash antibodies associated with resistance to experimental challenge with influenza A wild-type virus.* J Clin Microbiol, 1986. **24**(1): p. 157-60.
24. Clements, M.L., et al., *Resistance of adults to challenge with influenza A wild-type virus after receiving live or inactivated virus vaccine.* J Clin Microbiol, 1986. **23**(1): p. 73-6.
25. Powers, D.C., *Effect of age on serum immunoglobulin G subclass antibody responses to inactivated influenza virus vaccine.* J Med Virol, 1994. **43**(1): p. 57-61.
26. Wagner, D.K., et al., *Analysis of immunoglobulin G antibody responses after administration of live and inactivated influenza A vaccine indicates that nasal wash immunoglobulin G is a transudate from serum.* J Clin Microbiol, 1987. **25**(3): p. 559-62.

27. Garcon, N.M., et al., *Serum IgG subclass antibody responses in children vaccinated with influenza virus antigens by live attenuated or inactivated vaccines.* Antiviral Res, 1990. **14**(2): p. 109-16.
28. Burton, D.R., L. Gregory, and R. Jefferis, *Aspects of the molecular structure of IgG subclasses.* Monogr Allergy, 1986. **19**: p. 7-35.
29. Spiegelberg, H.L., *Biological activities of immunoglobulins of different classes and subclasses.* Adv Immunol, 1974. **19**(0): p. 259-94.
30. Betts, R.F., D. O'Brien, and M. Menegus, *A comparison of the protective benefit of influenza (FLU) vaccine in reducing hospitalization of patients infected with FLU A or FLU B. (A257).* Clin Infect Dis, 1993. **17**: p. 573.
31. Beck, J.M., M.J. Rosen, and H.H. Peavy, *Pulmonary complications of HIV infection. Report of the Fourth NHLBI Workshop.* Am J Respir Crit Care Med, 2001. **164**(11): p. 2120-6.
32. Conley, L.J., et al., *The association between cigarette smoking and selected HIV-related medical conditions.* Aids, 1996. **10**(10): p. 1121-6.
33. Lin, J.C. and K.L. Nichol, *Excess mortality due to pneumonia or influenza during influenza seasons among persons with acquired immunodeficiency syndrome.* Arch Intern Med, 2001. **161**(3): p. 441-6.
34. Neuzil, K.M., et al., *Influenza-associated morbidity and mortality in young and middle-aged women.* Jama, 1999. **281**(10): p. 901-7.
35. Fine, A.D., et al., *Influenza A among patients with human immunodeficiency virus: an outbreak of infection at a residential facility in New York City.* Clin Infect Dis, 2001. **32**(12): p. 1784-91.
36. Klein, M., et al., *Influenza infection is a primary cause of febrile respiratory illness in HIV-infection adults despite vaccination.* Clin Infect Dis. **in press**.
37. Ragni, M.V., et al., *Antibody responses to immunization of patients with hemophilia with and without evidence of human immunodeficiency virus (human T-lymphotropic virus type III) infection.* J Lab Clin Med, 1987. **109**(5): p. 545-9.
38. Redfield, R.R., et al., *Disseminated vaccinia in a military recruit with human immunodeficiency virus (HIV) disease.* N Engl J Med, 1987. **316**(11): p. 673-6.
39. Chadwick, E.G., et al., *Serologic response to standard inactivated influenza vaccine in human immunodeficiency virus-infected children.* Pediatr Infect Dis J, 1994. **13**(3): p. 206-11.

40. Huang, K.L., et al., *Antibody responses after influenza and pneumococcal immunization in HIV-infected homosexual men.* Jama, 1987. **257**(15): p. 2047-50.
41. Staprans, S.I., et al., *Activation of virus replication after vaccination of HIV-1-infected individuals.* J Exp Med, 1995. **182**(6): p. 1727-37.
42. Kroon, F.P., et al., *Antibody response after influenza vaccination in HIV-infected individuals: a consecutive 3-year study.* Vaccine, 2000. **18**(26): p. 3040-9.
43. Miotti, P.G., et al., *The influence of HIV infection on antibody responses to a two-dose regimen of influenza vaccine.* Jama, 1989. **262**(6): p. 779-83.
44. Atashili, J., L. Kalilani, and A.A. Adimora, *Efficacy and clinical effectiveness of influenza vaccines in HIV-infected individuals: a meta-analysis.* BMC Infect Dis, 2006. **6**: p. 138.
45. Nichol, K.L., *Booster doses of influenza vaccine.* JAMA, 1996. **276**(23): p. 1857.
46. Iorio, A.M., et al., *Immunogenicity of influenza vaccine (1993-94 winter season) in HIV-seropositive and -seronegative ex-intravenous drug users.* Vaccine, 1997. **15**(1): p. 97-102.
47. Smith, N.M., et al., *Prevention and Control of Influenza: recommendations of the Advisory Committee on Immunization Practices (ACIP).* MMWR Recomm Rep, 2006. **55**(RR-10): p. 1-42.
48. *Antibody responses and reactogenicity of graded doses of inactivated influenza A/New Jersey/76 whole-virus vaccine in humans.* J Infect Dis, 1977. **136 Suppl**: p. S475-83.
49. Roos-Van Eijndhoven, D.G., et al., *Randomized controlled trial of seroresponses to double dose and booster influenza vaccination in frail elderly subjects.* J Med Virol, 2001. **63**(4): p. 293-8.
50. Treanor, J.J., et al., *Safety and immunogenicity of an inactivated subvirion influenza A (H5N1) vaccine.* N Engl J Med, 2006. **354**(13): p. 1343-51.
51. Anema, A., et al., *Efficacy of influenza vaccination among HIV+ patients: A systematic review and meta-analysis.* Submitted, 2007.
52. Biologicals, G., *Prescribing Information, Influenza Virus Vaccine (Fluarix™,) 2005-2006 Formula.*, in *GlaxoSmithKline Biologicals.* 2005: Rixensart, Belgium.
53. Rose, G.W. and C.L. Cooper, *Fluarix, inactivated split-virus influenza vaccine.* Expert Opin Biol Ther, 2006. **6**(3): p. 301-10.

54. Cooper, C.L., et al., *CPG 7909 adjuvant improves hepatitis B virus vaccine seroprotection in antiretroviral-treated HIV-infected adults.* Aids, 2005. **19**(14): p. 1473-9.

30. Appendices

Appendix A: Declaration of Helsinki

Policy

WORLD MEDICAL ASSOCIATION DECLARATION OF HELSINKI
Ethical Principles for Medical Research Involving Human Subjects

Adopted by the 18th WMA General Assembly, Helsinki, Finland, June 1964, and amended by the
29th WMA General Assembly, Tokyo, Japan, October 1975
35th WMA General Assembly, Venice, Italy, October 1983
41st WMA General Assembly, Hong Kong, September 1989
48th WMA General Assembly, Somerset West, Republic of South Africa, October 1996
and the 52nd WMA General Assembly, Edinburgh, Scotland, October 2000
Note of Clarification on Paragraph 29 added by the WMA General Assembly, Washington 2002
Note of Clarification on Paragraph 30 added by the WMA General Assembly, Tokyo 2004

A. INTRODUCTION

1. The World Medical Association has developed the Declaration of Helsinki as a statement of ethical principles to provide guidance to physicians and other participants in medical research involving human subjects. Medical research involving human subjects includes research on identifiable human material or identifiable data.

2. It is the duty of the physician to promote and safeguard the health of the people. The physician's knowledge and conscience are dedicated to the fulfillment of this duty.

3. The Declaration of Geneva of the World Medical Association binds the physician with the words, "The health of my patient will be my first consideration," and the International Code of Medical Ethics declares that, "A physician shall act only in the patient's interest when providing medical care which might have the effect of weakening the physical and mental condition of the patient."

4. Medical progress is based on research which ultimately must rest in part on experimentation involving human subjects.

5. In medical research on human subjects, considerations related to the well-being of the human subject should take precedence over the interests of science and society.

6. The primary purpose of medical research involving human subjects is to improve prophylactic, diagnostic and therapeutic procedures and the understanding of the aetiology and pathogenesis of disease. Even the best proven prophylactic, diagnostic, and therapeutic methods must continuously be challenged through research for their effectiveness, efficiency, accessibility and quality.

7. In current medical practice and in medical research, most prophylactic, diagnostic and therapeutic procedures involve risks and burdens.

8. Medical research is subject to ethical standards that promote respect for all human beings and protect their health and rights. Some research populations are vulnerable and need special protection. The particular needs of the economically and medically disadvantaged must be recognized. Special attention is also required for those who cannot give or refuse consent for themselves, for those who may be subject to giving consent under duress, for those who will not benefit personally from the research and for those for whom the research is combined with care.

9. Research Investigators should be aware of the ethical, legal and regulatory requirements for research on human subjects in their own countries as well as applicable international requirements. No national ethical, legal or regulatory requirement should be allowed to reduce or eliminate any of the protections for human subjects set forth in this Declaration.

B. BASIC PRINCIPLES FOR ALL MEDICAL RESEARCH

10. It is the duty of the physician in medical research to protect the life, health, privacy, and dignity of the human subject.

11. Medical research involving human subjects must conform to generally accepted scientific principles, be based on a thorough knowledge of the scientific literature, other relevant sources of information, and on adequate laboratory and, where appropriate, animal experimentation.

12. Appropriate caution must be exercised in the conduct of research which may affect the environment, and the welfare of animals used for research must be respected.

13. The design and performance of each experimental procedure involving human subjects should be clearly formulated in an experimental protocol. This protocol should be submitted for consideration, comment, guidance, and where appropriate, approval to a specially appointed ethical review committee, which must be independent of the investigator, the sponsor or any other kind of undue influence. This independent committee should be in conformity with the laws and regulations of the country in which the research experiment is performed. The committee has the right to monitor ongoing trials. The researcher has the obligation to provide monitoring information to the committee, especially any serious adverse events. The researcher should also submit to the committee, for review, information regarding funding, sponsors, institutional affiliations, other potential conflicts of interest and incentives for subjects.

14. The research protocol should always contain a statement of the ethical considerations involved and should indicate that there is compliance with the principles enunciated in this Declaration.

15. Medical research involving human subjects should be conducted only by scientifically qualified persons and under the supervision of a clinically competent medical person. The responsibility for the human subject must always rest with a medically qualified person and never rest on the subject of the research, even though the subject has given consent.

16. Every medical research project involving human subjects should be preceded by careful assessment of predictable risks and burdens in comparison with foreseeable benefits to the subject or to others. This does not preclude the participation of healthy volunteers in medical research. The design of all studies should be publicly available.

17. Physicians should abstain from engaging in research projects involving human subjects unless they are confident that the risks involved have been adequately assessed and can be satisfactorily managed. Physicians should cease any investigation if the risks are found to outweigh the potential benefits or if there is conclusive proof of positive and beneficial results.

18. Medical research involving human subjects should only be conducted if the importance of the objective outweighs the inherent risks and burdens to the subject. This is especially important when the human subjects are healthy volunteers.

19. Medical research is only justified if there is a reasonable likelihood that the populations in which the research is carried out stand to benefit from the results of the research.

20. The subjects must be volunteers and informed participants in the research project.

21. The right of research subjects to safeguard their integrity must always be respected. Every precaution should be taken to respect the privacy of the subject, the confidentiality of the patient's information and to minimize the impact of the study on the subject's physical and mental integrity and on the personality of the subject.

22. In any research on human beings, each potential subject must be adequately informed of the aims, methods, sources of funding, any possible conflicts of interest, institutional affiliations of the researcher, the anticipated benefits and potential risks of the study and the discomfort it may entail. The subject should be informed of the right to abstain from participation in the study or to withdraw consent to participate at any time without reprisal. After ensuring that the subject has understood the information, the physician should then obtain the subject's freely-given informed consent, preferably in writing. If the consent cannot be obtained in writing, the non-written consent must be formally documented and witnessed.

23. When obtaining informed consent for the research project the physician should be particularly cautious if the subject is in a dependent relationship with the physician or may consent under duress. In that case the informed consent should be obtained by a well-informed physician who is not engaged in the investigation and who is completely independent of this relationship.

24. For a research subject who is legally incompetent, physically or mentally incapable of giving consent or is a legally incompetent minor, the investigator must obtain informed consent from the legally authorized representative in

accordance with applicable law. These groups should not be included in research unless the research is necessary to promote the health of the population represented and this research cannot instead be performed on legally competent persons.

25. When a subject deemed legally incompetent, such as a minor child, is able to give assent to decisions about participation in research, the investigator must obtain that assent in addition to the consent of the legally authorized representative.

26. Research on individuals from whom it is not possible to obtain consent, including proxy or advance consent, should be done only if the physical/mental condition that prevents obtaining informed consent is a necessary characteristic of the research population. The specific reasons for involving research subjects with a condition that renders them unable to give informed consent should be stated in the experimental protocol for consideration and approval of the review committee. The protocol should state that consent to remain in the research should be obtained as soon as possible from the individual or a legally authorized surrogate.

27. Both authors and publishers have ethical obligations. In publication of the results of research, the investigators are obliged to preserve the accuracy of the results. Negative as well as positive results should be published or otherwise publicly available. Sources of funding, institutional affiliations and any possible conflicts of interest should be declared in the publication. Reports of experimentation not in accordance with the principles laid down in this Declaration should not be accepted for publication.

C. ADDITIONAL PRINCIPLES FOR MEDICAL RESEARCH COMBINED WITH MEDICAL CARE

28. The physician may combine medical research with medical care, only to the extent that the research is justified by its potential prophylactic, diagnostic or therapeutic value. When medical research is combined with medical care, additional standards apply to protect the patients who are research subjects.

29. The benefits, risks, burdens and effectiveness of a new method should be tested against those of the best current prophylactic, diagnostic, and therapeutic methods. This does not exclude the use of placebo, or no treatment, in studies where no proven prophylactic, diagnostic or therapeutic method exists.[1]

30. At the conclusion of the study, every patient entered into the study should be assured of access to the best proven prophylactic, diagnostic and therapeutic methods identified by the study.[2]

31. The physician should fully inform the patient which aspects of the care are related to the research. The refusal of a patient to participate in a study must never interfere with the patient-physician relationship.

32. In the treatment of a patient, where proven prophylactic, diagnostic and therapeutic methods do not exist or have been ineffective, the physician, with informed consent from the patient, must be free to use unproven or new prophylactic, diagnostic and therapeutic measures, if in the physician's judgement it offers hope of saving life, re-establishing health or alleviating suffering. Where possible, these measures should be made the object of research, designed to evaluate their safety and efficacy. In all cases, new information should be recorded and, where appropriate, published. The other relevant guidelines of this Declaration should be followed.

---

[1] **Note of clarification on paragraph 29 of the WMA Declaration of Helsinki**

The WMA hereby reaffirms its position that extreme care must be taken in making use of a placebo-controlled trial and that in general this methodology should only be used in the absence of existing proven therapy. However, a placebo-controlled trial may be ethically acceptable, even if proven therapy is available, under the following circumstances:

- Where for compelling and scientifically sound methodological reasons its use is necessary to determine the efficacy or safety of a prophylactic, diagnostic or therapeutic method; or

- Where a prophylactic, diagnostic or therapeutic method is being investigated for a minor condition and the patients who receive placebo will not be subject to any additional risk of serious or irreversible harm.

All other provisions of the Declaration of Helsinki must be adhered to, especially the need for appropriate ethical and scientific review.

[2] **Note of clarification on paragraph 30 of the WMA Declaration of Helsinki**

The WMA hereby reaffirms its position that it is necessary during the study planning process to identify post-trial access by study participants to prophylactic, diagnostic and therapeutic procedures identified as beneficial in the study or access to other appropriate care. Post-trial access arrangements or other care must be described in the study protocol so the ethical review committee may consider such arrangements during its review.

9.10.2004

Appendix B: Subject Diary

PATIENT DIARY

CTN237

A Controlled Trial to Assess the Immunogenicity and Efficacy of Three Influenza Vaccine Dosing Strategies in HIV Infected Adults

Subject Number ____________

Subject Initials ____________

Date of issue ____/____/________
D D M M M Y Y Y Y

Visit No: 2☐ 3☐ 4☐

Contact information:

Site # / Investigator: (in block letters) ____________

Hospital / Clinic: ____________

Telephone number(s): ____________

Date of next visit: ____________

Version 1.0 20-May-2008

Medication Name	How much each time (ie. 2 pills, 20 mg each)	How often (ie. once a day)	Route (e.g., mouth, topical)	Reason you took medication	Start Date (DD/MMM/YYYY)	Stop Date (DD/MMM/YYYY)
					/ /	/ /
					/ /	/ /
					/ /	/ /
					/ /	/ /

Other Health Changes: Please record below any other health changes noted since your last study visit.

Description of Health Concern or Change in Pre-Existing Condition	Did you take any medications?	Did you see a health care provider?	Severity 1 – Mild 2 – Moderate 3 – Severe	Start Date (DD/MMM/YYYY)	Stop Date (DD/MMM/YYYY)
	☐ No ☐ Yes	☐ No ☐ Yes		/ /	/ /
	☐ No ☐ Yes	☐ No ☐ Yes		/ /	/ /
	☐ No ☐ Yes	☐ No ☐ Yes		/ /	/ /
	☐ No ☐ Yes	☐ No ☐ Yes		/ /	/ /

Instructions for Injection Reaction Diary: Please complete the table below on a daily basis for 7 days following your study injection.
Please note that the 15 minutes post injection assessment will be completed by you with your study physician or nurse.
Record the most severe assessment for each day.

Day Post Injection	0		1	2	3	4	5	6	7	If there are no symptoms after Day 7 please check, "N/A". If symptoms continue after Day 7, record date of last day of symptoms:
	15 min	PM								
Date (DD/MM/YYYY)										
Please record oral temperature measured in the evening of each of the day listed or the highest temperature recorded on that day.										
Temperature (Oral)		__.__ °C	__.__ °C	__.__ °C	__.__ °C	__.__ °C	__.__ °C	__.__ °C	__.__ °C	
Please check the area at or near the injection site for signs of redness or swelling. If any, please measure the length/diameter of these signs using the ruler or transparency provided to you. Please enter the length/diameter of redness and swelling at or near the injection site. (This will either be the reading indicated on the rule or the number printed on the transparency										
Redness at or near injection site	__ mm	__ mm	__ mm	__ mm	__ mm	__ mm	__ mm	__ mm	__ mm	__/__/20__ D D M M M Y Y Y Y ☐ N/A
Swelling at or near injection site	__ mm	__ mm	__ mm	__ mm	__ mm	__ mm	__ mm	__ mm	__ mm	__/__/20__ D D M M M Y Y Y Y ☐ N/A
Please indicate the severity of these other symptoms using the following codes:					0 = None<br>1 = Mild (no interference with activity)<br>2 = Moderate (some interference with activity)<br>3 = Severe (significant, prevents daily activity)					
Pain at or near injection site										__/__/20__ D D M M M Y Y Y Y ☐ N/A
Malaise (not feeling well)										__/__/20__ D D M M M Y Y Y Y ☐ N/A
Headache										__/__/20__ D D M M M Y Y Y Y ☐ N/A
Fatigue (feeling tired)										__/__/20__ D D M M M Y Y Y Y ☐ N/A

Version 1.0 20-May-2008

Instructions for Study Period Flu Symptoms Diary: Please complete if flu-like symptoms occur during study period. Please complete only if you have a respiratory infection defined as a temperature >38.0° C and one of the following symptom. Please also contact your research coordinator to discuss symptoms to determine if you need to attend the clinic for a respiratory infection clinical assessment and nasal swab.

Symptom		Severity<br>Indicate the severity using the following codes:<br>1 = Mild (no interference with activity)<br>2 = Moderate (some interference with activity)<br>3 = Severe (significant, prevents daily activity)	Start	End
Oral Temperature	__.__ °C		__/____/20____<br>D D M M M Y Y Y Y	__/____/20____<br>D D M M M Y Y Y Y
Feverishness/chills	Yes ☐<br>No ☐		__/____/20____<br>D D M M M Y Y Y Y	__/____/20____<br>D D M M M Y Y Y Y
Cough	Yes ☐<br>No ☐		__/____/20____<br>D D M M M Y Y Y Y	__/____/20____<br>D D M M M Y Y Y Y
Shortness of breath or rapid breathing	Yes ☐<br>No ☐		__/____/20____<br>D D M M M Y Y Y Y	__/____/20____<br>D D M M M Y Y Y Y
Running nose	Yes ☐<br>No ☐		__/____/20____<br>D D M M M Y Y Y Y	__/____/20____<br>D D M M M Y Y Y Y
Sore Throat	Yes ☐<br>No ☐		__/____/20____<br>D D M M M Y Y Y Y	__/____/20____<br>D D M M M Y Y Y Y
Aching muscles	Yes ☐<br>No ☐		__/____/20____<br>D D M M M Y Y Y Y	__/____/20____<br>D D M M M Y Y Y Y
Other, Specify<br>________________	Yes ☐<br>No ☐		__/____/20____<br>D D M M M Y Y Y Y	__/____/20____<br>D D M M M Y Y Y Y
Other, Specify<br>________________	Yes ☐<br>No ☐		__/____/20____<br>D D M M M Y Y Y Y	__/____/20____<br>D D M M M Y Y Y Y

Version 1.0 20-May-2008

A Thermometer, Transparency and Ruler to be provided as tools for measurement of symptoms for Flu and Vaccine Reactions. Below is a sample of the Transparency and Ruler.

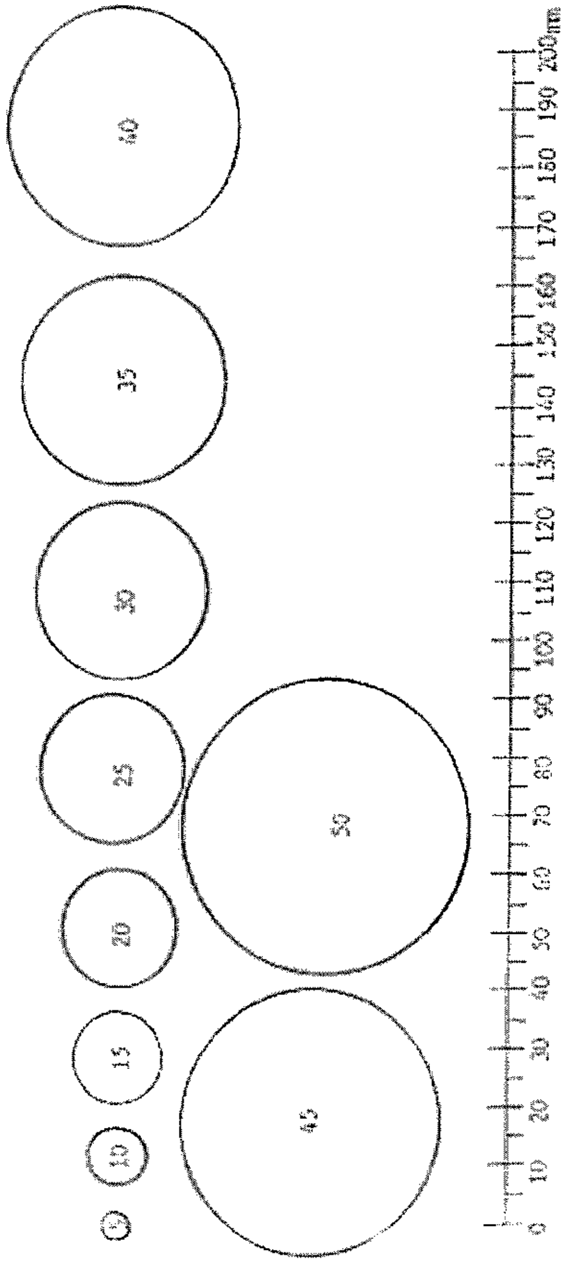

Copyright 2006, Vaccine Evaluation Center
© All rights reserved

Version 1.0 20-May-2008

Appendix C: Study Period Flu Symptom Questionnaire

CTN 237
Influenza Vaccine in HIV
Study Period Flu Symptoms
Page 1 of 2

Week ☐☐

Randomization ID # ☐☐☐☐☐☐

Subject's Initials ☐☐☐

Date of Visit ☐☐ / ☐☐☐ / ☐☐ (Day / Month / Year)

This form should only be done if the subject develops flu-like symptoms.

1. Start Date of Symptoms: ☐☐ / ☐☐☐ / ☐☐ (Day / Month / Year)

2. Symptoms

a. oral temperature ☐☐.☐ °C

	1	2				
b. feverishness/chills.........	☐ Yes	☐ No	→ If Yes,	☐ 1 Mild	☐ 2 Moderate	☐ 3 Severe
c. cough..............................	☐ Yes	☐ No	→ If Yes,	☐ 1 Mild	☐ 2 Moderate	☐ 3 Severe
d. tachypnea/dyspnea..........	☐ Yes	☐ No	→ If Yes,	☐ 1 Mild	☐ 2 Moderate	☐ 3 Severe
e. wheezing/stridor...........	☐ Yes	☐ No	→ If Yes,	☐ 1 Mild	☐ 2 Moderate	☐ 3 Severe
f. rhinorrhea......................	☐ Yes	☐ No	→ If Yes,	☐ 1 Mild	☐ 2 Moderate	☐ 3 Severe
g. sore throat.....................	☐ Yes	☐ No	→ If Yes,	☐ 1 Mild	☐ 2 Moderate	☐ 3 Severe
h. myalgia..........................	☐ Yes	☐ No	→ If Yes,	☐ 1 Mild	☐ 2 Moderate	☐ 3 Severe

3. Was Nasopharyngeal viral swab done as per protocol? ☐ 1 Yes ☐ 2 No

4. Additional non-study investigations

a. Sputum culture done? ☐ 1 Yes → Date of Test ☐☐ / ☐☐☐ / ☐☐ (Day / Month / Year) ☐ 2 No

Results: ☐ 1 Positive ☐ 2 Negative

Type of organism isolated: ☐

b. Chest x-ray done? ☐ 1 Yes → Date of Test ☐☐ / ☐☐☐ / ☐☐ (Day / Month / Year) ☐ 2 No

Pneumonic infiltrate identified? ☐ 1 Yes ☐ 2 No

CTN 237
Influenza Vaccine in HIV
Study Period Flu Symptoms
Page 2 of 2

Week ☐☐

Randomization ID # ☐☐☐☐☐☐

Subject's Initials ☐☐☐

Date of Visit ☐☐ / ☐☐☐ / ☐☐ (Day / Month / Year)

4. Additional non-study investigations (Continued...)

c. Additional nasopharyngeal viral swab done?

☐ 1 Yes → **Date of Test** ☐☐ / ☐☐☐ / ☐☐ (Day / Month / Year) **Results** ☐ 1 Positive ☐ 2 Negative

☐ 2 No

d. Other influenza findings, describe ☐

5. Does the subject have influenza?

☐ 1 Yes → ☐ 1 A ☐ 2 B

☐ 2 No

6. Did the subject receive any flu medications? ☐ 1 Yes, list below ☐ 2 No

(update Concomitant Medications form)

1	
2	
3	

7. End Date of Symptoms: ☐☐ / ☐☐☐ / ☐☐ (Day / Month / Year)

Appendix D: Employment, Health Care Resource Utilization, and Health Utilities Index Questionnaire

CTN 237
Influenza Vaccine in HIV
Baseline History
1 page

Screening ID # ☐☐☐☐☐☐

Randomization ID # ☐☐☐☐☐☐

Subject's Initials ☐☐☐

BASELINE

Date of Visit ☐☐ / ☐☐☐ / ☐☐
Day / Month / Year

1. Employment

a. Patient's current employment activities? (check only the one that best applies)

☐ 1 Working full-time
☐ 2 Unemployed
☐ 3 Homemaker
☐ 4 Working part-time
☐ 5 Full-time student
☐ 6 Other, Specify: ☐
☐ 7 Not working, on disability pay
☐ 8 Receiving employment insurance, social assiatance

b. Patient's estimate of the typical annual income for someone in his/her field of employment.

☐ 1 < $15,000
☐ 2 $15,001 - 25,000
☐ 3 $25,001 - 35,000
☐ 4 $35,001 - 50,000
☐ 5 $50,001 - 70,000
☐ 6 >$ 70,000
☐ 7 Refused (N/A)

CTN 237
Influenza Vaccine in HIV
Health Care Resource Utilization
1 page

Week ☐☐

Randomization ID # ☐☐☐☐☐☐

Day	Month	Year

Subject's Initials ☐☐☐ Date of Visit ☐☐ / ☐☐☐ / ☐☐

1. Follow-Up Assessments

	No	Yes	Unknown
a. Since the last visit, has the patient seen a health care provider for a non-study visit?	☐	☐	☐

If Yes, # of visits to a HIV clinic ☐☐ # of visits to physician ☐☐

b. Since the last visit, approximately how many days has the patient missed work due to illness? (If not in paid employment for the entire period, enter N/A) ☐☐☐

2. Hospitalization

a. Type of hospital ☐ 1 Hospital ☐ 2 Long-term care facility

b. Was hospitalization AIDS-related? ☐ 1 Yes ☐ 2 No

Admission Date			ER Admission			Total Stay	Length of Stay
Day	Month	Year	No	Yes	Event	(Days)	ICU/CCU/HDU
☐☐ /	☐☐☐ /	☐☐	☐	☐		☐☐☐	☐☐
☐☐ /	☐☐☐ /	☐☐	☐	☐		☐☐☐	☐☐
☐☐ /	☐☐☐ /	☐☐	☐	☐		☐☐☐	☐☐
☐☐ /	☐☐☐ /	☐☐	☐	☐		☐☐☐	☐☐

CTN 237
Influenza Vaccine in HIV
Health Utilities Index

Page 1 of 4

Week ☐☐

Randomization ID # ☐☐☐☐☐☐

Subject's Initials ☐☐☐

Date of Visit ☐☐ / ☐☐☐ / ☐☐
(Day / Month / Year)

Permission for use of this form has been obtained from Health Utilities Inc. Canada

Instructions: This questionnaire contains a set of questions which ask about various aspects of your health. When answering these questions please think about your health and your ability to do things on a day-to-day basis, today. Please focus your answers on your overall abilities, disabilities and how you feel today.

You may feel that some of these questions do not apply to you, but it is important that we ask the same questions of everyone. Also, a few questions are similar; please excuse the apparent overlap and answer each question independently.

Please read each question and consider your answers carefully. For each question, please select one answer that best describes your level of ability or disability today. Please indicate the selected answer by placing an X in the box beside the answer.

All information you provide is confidential. There are no right or wrong answers; what we want is your opinion about your abilities and feelings.

1. Which one of the following best describes your ability, today, to see well enough, to read ordinary newsprint?
   - ☐ a. Able to see well enough without glasses or contact lenses
   - ☐ b. Able to see well enough with glasses or contact lenses
   - ☐ c. Unable to see well enough even with glasses or contact lenses
   - ☐ d. Unable to see at all

2. Which one of the following best describes your ability, today, to see well enough to recognize a friend on the other side of the street?
   - ☐ a. Able to see well enough without glasses or contact lenses
   - ☐ b. Able to see well enough with glasses or contact lenses
   - ☐ c. Unable to see well enough even with glasses or contact lenses
   - ☐ d. Unable to see at all

CTN 237
Influenza Vaccine in HIV
Health Utilities Index

Page 2 of 4

Week ☐☐

Randomization ID # ☐☐☐☐☐☐

Day / Month / Year
☐☐ / ☐☐☐ / ☐☐

Subject's Initials ☐☐☐

3. Which one of the following best describes your ability, today, to hear what was said in a group conversation with at least three other people?
   - ☐ a. Able to hear what was said without a hearing aid
   - ☐ b. Able to hear what was said with a hearing aid
   - ☐ c. Unable to hear what was said even with a hearing aid
   - ☐ d. Unable to hear what was said, but did not wear a hearing aid
   - ☐ e. Unable to hear at all

4. Which one of the following best describes your ability, today, to hear what was said in a conversation with one other person in a quiet room?
   - ☐ a. Able to hear what was said without a hearing aid
   - ☐ b. Able to hear what was said with a hearing aid
   - ☐ c. Unable to hear what was said even with a hearing aid
   - ☐ d. Unable to hear what was said, but did not wear a hearing aid
   - ☐ e. Unable to hear at all

5. Which one of the following best describes your ability, today, to be understood when speaking your own language with people who you do not know?
   - ☐ a. Able to be understood completely
   - ☐ b. Able to be understood partially
   - ☐ c. Unable to be understood
   - ☐ d. Unable to speak at all

6. Which one of the following best describes your ability, today, to be understood when speaking with people who know you well?
   - ☐ a. Able to be understood completely
   - ☐ b. Able to be understood partially
   - ☐ c. Unable to be understood
   - ☐ d. Unable to speak at all

7. Which one of the following best describes how you have been feeling today?
   - ☐ a. Happy and interested in life
   - ☐ b. Somewhat happy
   - ☐ c. Somewhat unhappy
   - ☐ d. Very Unhappy
   - ☐ e. So unhappy that life was not worthwhile

8. Which one of the following best describes the pain and discomfort you have experienced today?
   - ☐ a. Free of pain and discomfort
   - ☐ b. Mild to moderate pain or discomfort that prevented no activities
   - ☐ c. Moderate painor discomfort that prevented a few activities
   - ☐ d. Moderate to severe pain or discomfort that prevented some activities
   - ☐ e. Severe pain or discomfort that prevented most activities

CTN 237
Influenza Vaccine in HIV
Health Utilities Index

Page 3 of 4

Week ☐☐

Randomization ID # ☐☐☐☐☐☐

Day / Month / Year
☐☐ / ☐☐☐ / ☐☐

Subject's Initials ☐☐☐

9. Which one of the following best describes your ability, today, to walk?
Note: Walking equipment refers to mechanical supports such as braces, a cane, crutches or a walker
- ☐ a. Able to walk around the neighborhood without difficulty, and without walking equipment
- ☐ b. Able to walk around the neighborhood with difficulty; but did not require walking equipment or the help of another person
- ☐ c. Able to walk around the neighborhood with walking equipment, but without the help of another person
- ☐ d. Able to walk only short distances with walking equipment, and required a wheelchair to get around the neighborhood
- ☐ e. Unable to walk alone, even with walking equipment. Able to walk short distances with the help of another person, and required a wheelchair to get around the neighborhood
- ☐ f. Unable to walk at all

10. Which one of the following best describes your ability, today, to use your hands and fingers?
Note: Special tools refers to hooks for buttoning clothes, gripping devices for opening jars or lifting small items, and other devices to compensate for limitations of hands or fingers.
- ☐ a. Full use of two hands and ten fingers
- ☐ b. Limitations in the use of hands or fingers, but did not require special tools or the help of another person
- ☐ c. Limitations in the use of hands or fingers, independent with use of special tools (did not require the help of another person).
- ☐ d. Limitations in the use of hands or fingers, required the help of another person for some tasks (not independent even with use of special tools).
- ☐ e. Limitations in the use of hands or fingers, required the help of another person for most tasks (not independent even with use of special tools).
- ☐ f. Limitations in the use of hands or fingers, required the help of another person for all tasks (not independent even with use of special tools).

11. Which one of the following best describes your ability, today, to remember things?
- ☐ a. Able to remember most things.
- ☐ b. Somewhat forgetful.
- ☐ c. Very forgetful.
- ☐ d. Unable to remember anything at all.

12. Which one of the following best describes your ability, today, to think and solve day to day problems?
- ☐ a. Able to think clearly and solve day to day problems.
- ☐ b. Had a little difficulty when trying to think and solve day to day problems.
- ☐ c. Had some difficulty when trying to think and solve day to day problems.
- ☐ d. Had great difficulty when trying to think and solve day to day problems.
- ☐ e. Unable to think or solve day to day problems.

CTN 237
Influenza Vaccine in HIV
Health Utilities Index

Page 4 of 4

Week ☐☐

Randomization ID # ☐☐☐☐☐☐

Day ☐☐ / **Month** ☐☐☐ / **Year** ☐☐

Subject's Initials ☐☐☐

13. Which one of the following best describes your ability, today, to perform basic activities?
- ☐ a. Eat, bathe, dress and use the toilet normally.
- ☐ b. Eat, bathe, dress or use the toilet independently with difficulty.
- ☐ c. Required mechanical equipment to eat, bathe, dress or use the toilet independently.
- ☐ d. Required the help of another person to eat, bathe, dress or use the toilet.

14. Which one of the following best describes how you have been feeling today?
- ☐ a. Generally happy and free from worry.
- ☐ b. Occasionally fretful, angry, irritable, anxious or depressed.
- ☐ c. Often fretful, angry, irritable, anxious or depressed.
- ☐ d. Almost always fretful, angry, irritable, anxious or depressed.
- ☐ e. Extremely fretful, angry, irritable, anxious or depressed; to the point of needing professional help.

15. Which one of the following best describes the pain or discomfort you have experienced today?
- ☐ a. Free of pain and discomfort.
- ☐ b. Occasional pain or discomfort. Discomfort relieved by non-prescription drugs or self-control activity without disruption of normal activities.
- ☐ c. Frequent pain or discomfort. Discomfort relieved by oral medicines with occasional disruption of normal activities.
- ☐ d. Frequent pain or discomfort; frequent disruption of normal activities. Discomfort required prescription narcotics for relief.
- ☐ e. Severe pain or discomfort. Pain not relieved by drugs and constantly disrupted normal activities.

16. Overall, how would you rate your health today?
☐ a. Excellent. ☐ b. Very good. ☐ c. Good. ☐ d. Fair. ☐ e. Poor.

17. How did you complete the questionnaire? Please select the one answer that best describes your situation.
- ☐ a. By myself, without any help from anyone else.
- ☐ b. By myself, except someone else placed an X in the answers on the questionnaire form for me.
- ☐ c. With the help of someone else.
- ☐ d. This questionnaire was completed by a family member, without help from the subject or patient.
- ☐ e. This questionnaire was completed by a nurse or other health professional, without help from the subject or patient. Please specify type of health professional: ________
- ☐ f. This questionnaire was completed by another person, without help from the subject or patient. Please specify relationship to subject or patient: ________

Staff Initials ☐☐☐

Appendix E: Model Informed Consent

PARTICIPANT INFORMED CONSENT FORM

STUDY TITLE: A Controlled Trial to Assess the Immunogenicity and Efficacy of Three Influenza Vaccine Dosing Strategies in HIV Infected Adults

STUDY DOCTOR: <Insert Dr. Name>

STUDY SITE: <Insert Institution Name>

TELEPHONE: <Insert Telephone Number>

Emergency 24 hour Contact: <Insert Contact Information>

You are being asked to participate in a research study of a licensed vaccine for influenza, Fluviral®. The word licensed means the vaccine is approved for sale. You are being asked to take part in this study because the study doctor feels that you meet the qualifications of the study. You will have a copy of this form to review at your leisure or to ask advice from others. To decide whether or not you want to be part of this research, you should understand the study risks and benefits in order to make an informed decision. This process is known as informed consent. This consent form describes the purpose, procedures, possible benefits and risks of the study. This form will also explain how your medical information will be used and who may see it. Once you understand the study, you will be asked to sign this form if you wish to participate.

The study doctor or staff will answer any questions you may have about this form or about the study. Please read this document carefully and do not hesitate to ask anything about this information. This form may contain words that you do not understand. Please ask the study doctor or staff to explain the words or information that you do not understand.

BACKGROUND TO THE STUDY:

Influenza (commonly called "the flu") is a highly infectious illness caused by a virus. It is passed on by breathing in air coughed by infected people. Symptoms include fever, chills, headache, sore throat, dry cough, tiredness, and muscle and joint aches. Influenza can be prevented by vaccination against the particular influenza viruses circulating during any particular flu season. A vaccine works by causing your body to make antibodies against a germ, such as influenza virus, so that if you are later exposed to the germ, you will not become infected by it. The effectiveness of a vaccine is not as good in those infected with HIV. Therefore, we are studying whether the influenza vaccine effectiveness is improved by providing an extra vaccine dose and/or increasing the dose of influenza vaccine.

Fluviral® is one of a number of commercially available vaccines which are designed to protect against influenza infection. These vaccines provide fairly effective protection in young adults, but are not as effective in people with HIV. A more effective vaccine could be very useful in preventing or diminishing the length of illness, hospitalization, and perhaps even the number of deaths in those with HIV which are attributed to influenza infection.

PURPOSE OF THIS STUDY

The purposes of this research study are: 1) to see if there is a difference in the quantity of protective influenza antibodies produced by different doses of the Fluviral vaccine and 2) to see if these different vaccine dosing schedules reduce flu-like illness and/or reduce laboratory documented influenza.

You may qualify to participate in this research study if you have HIV infection and are eligible to receive an influenza vaccine. You will not qualify for the study if you are having treatments (for example, steroids or other immune suppressing medications) or have conditions (other than HIV, for example, rheumatoid arthritis, lupus erythematosus, multiple sclerosis or hereditary immune deficiency conditions such as immune globulin deficiencies) that affect the immune system or have allergies to the vaccine (for example, chicken eggs or egg products).

You are being asked to participate in this study because you are between 18 and 60 years of age and have HIV infection. There will be approximately 285 participants enrolled into this study. The study will be conducted at approximately10 research sites in Canada.

This study at <Insert Institution Name> is under the direction of <Insert Dr. Name>.

STUDY VACCINES

In this research study, you will be randomized to one of three possible groups; Group A, one dose of Fluviral followed 28 days later by a second booster dose of Fluviral; Group B, a double dose of Fluviral followed 28 days later by a second double booster dose of Fluviral, or Group C, a single injection of Fluviral followed 28 days later by a saline injection. Groups A and B are the experimental arms. You will not be informed which group you have been randomized to. Injections will be given into the upper arm muscle (the deltoid).

The Group you will be randomized to is determined by chance (like flipping a coin). You have a 33% chance of receiving any one of the 3 vaccine schedules being studied.

STUDY PLAN

Participating in this study will require you to visit this clinic 5 times over a period of approximately 20 to 34 weeks. You will also be asked to phone the research coordinator to report any flu-like illnesses and determine if you need to attend the clinic for an additional clinical assessment and nasal swab. The total time commitment for this study will be approximately 5 to 6 hours. At each visit, study staff will briefly review the main points of the study with you to confirm if you wish to continue participation.

Screening Visit

At the first study visit (screening visit), the study doctor/coordinator will review the study with you and answer any questions you may have. The informed consent process will be explained and you will be given the informed consent form to read. If, after all your questions have been answered to your satisfaction and you have decided to participate, you will be asked to sign this informed consent document. You will be given a copy of the signed informed consent to keep. If you want, the study staff will inform your family doctor of your participation if he/she is not already aware. The first visit will require more time than other visits in order to allow time to ask questions and to review study requirements.

Prior to receiving any vaccine, you will need to have a brief evaluation and will be asked a series of questions regarding your reasons for participating in the study (screening questions). The research coordinator will also ask you questions about your medical history, use of prescription and over-the-counter medications or herbal preparations. Your next visit (Visit 2) will be scheduled at this time.

Study Vaccine Visits

At Visit 2, blood samples (approximately 20 mL or 4 teaspoons) will be collected for baseline laboratory tests and baseline information regarding your employment status and substance use will also be collected. You will be randomized (like flipping a coin) to one of the three different influenza vaccine schedules. You will then receive the first injection. The injection will be administered to your upper arm muscle.

You will return to the clinic approximately 4 weeks later (Visit 3) to receive the second injection. Additional blood samples (approximately 20 mL or 4 teaspoons) and information about any hospitalizations or missed work will be collected.
At both visit 2 and 3, prior to receiving each injection, you will be asked about medications you have taken, you will be asked if there have been changes in your health or in the medications you have been taking since the previous visit. You will be monitored by your study nurse and/or doctor following the injections and asked questions regarding any symptoms you are feeling. You will also be asked a series of questions about your ability to do things on a day-to-day basis.

You will be asked to document any respiratory infection, reaction to the vaccine or general health/medication changes occurring during the study. To assist you in this documentation you will be given a worksheet /diary to write down the details if you feel any symptoms or have any health/medication changes. Respiratory infections will be defined as a temperature more than 38.0° C (100.4° F) along with one or more of the following clinical symptoms: feverishness/chills; cough; shortness of breath or rapid breathing; wheezing; running nose; sore throat; aching muscles. You will also be given a thermometer to record your temperature if you feel like you have a fever and a ruler to measure any reactions to vaccine.

Study Follow Up Visits

During Visits 4 and 5, we will also draw a sample of blood from a vein in your arm. The amount of blood drawn at each visit will be approximately 20 mLs (4 teaspoons) Information about any hospitalizations or missed work will be collected. You will also be asked a series of questions about your ability to do things on a day-to-day basis

The blood samples from visits 2, 3, 4 and 5 will be used to determine your level of antibodies to influenza and measure other markers of your body's immunity to influenza. Subjects from only 2 sites (Dr. Klein and Dr. Cooper) will have additional blood samples drawn to examine another immune marker. You will be informed if you are part of this group. Your blood samples will be examined for this study only. All unused blood will be discarded.

Unscheduled Visits

If you develop a flu-like illnesses and your research coordinator determines you need to attend the clinic for an additional clinical assessment a visit will be scheduled as soon as possible. At this visit a nasal swab and information about any hospitalizations or missed work will be collected. You will also be asked a series of questions about your ability to do things on a day-to day basis. In addition, you will be asked about medications you have taken, you will be asked if there have been changes in your health or in the medications you have been taking since the previous visit.

If you choose to withdraw, or are withdrawn from study participation, you may be asked to return to the study clinic for a final visit assessment as detailed above as part of the study's ongoing safety evaluations.

<u>Schedule of Study Visits</u>

VISIT	1	2	3	4	5	Unscheduled
**When**	**Screening Visit**	**Study Day 0**<br>**Week 0**<br>**<u>Injection #1</u>**	Study Day 28<br>Week 4<br>**<u>Booster Injection</u>**	**Study Day 56**<br>**Week 8**	Study Day 140<br>Week20	As needed due to Flu-like Illness
**Details**	➢ inclusion/ exclusion<br>➢ written informed consent<br>➢ health questions<br>➢ screening questions<br>➢ physical exam if needed<br>➢ vital signs<br>➢ book visit 2	➢ health questions<br>➢ medication history<br>➢ Employment and substance use questions<br>➢ day-to-day activities questions<br>➢ physical exam if needed<br>➢ vital signs<br>➢ blood test 20 ml; influenza antibody.<br>➢ **injection in upper arm muscle**<br>➢ issue diary card<br>➢ book visit 3	➢ health questions<br>➢ medication review<br>➢ hospitalization and work questions<br>➢ day-to-day activities questions<br>➢ physical exam if needed<br>➢ exam of injection site<br>➢ vital signs<br>➢ blood test 20mls; influenza antibodies<br>➢ **injection in upper arm muscle**<br>➢ diary review<br>➢ **issue new diary card**<br>➢ book visit 4	➢ health questions<br>➢ medication review<br>➢ hospitalization and work questions<br>➢ day-to-day activities questions<br>➢ physical exam if needed<br>➢ blood test 20 ml; influenza antibodies<br>➢ diary review<br>➢ issue new diary card<br>➢ book visit 5	➢ health questions<br>➢ medication review<br>➢ hospitalization and work questions<br>➢ day-to-day activities questions<br>➢ physical exam if needed<br>➢ blood test 20 ml; influenza antibodies<br>➢ diary review	➢ health questions<br>➢ medication review<br>➢ hospitalization and work questions<br>➢ day-to-day activities questions<br>➢ physical exam if needed<br>➢ blood test 20 ml; influenza antibodies<br>➢ Nasal Swab<br>➢ diary review
Time	1 hour	1 hour	1 hour	45 minutes	45 minutes	45 minutes

RISKS/DISCOMFORTS

The most common reactions to any vaccine include redness and swelling at the injection site. It is possible that you may have pain, minor bruising, and hardness at the injection site. Another, but less common reaction to the influenza vaccine is to feel generally unwell. Thus, it is also possible that you have muscle aches, fatigue, headache, and/or mild fever, shivering, and sweating for a day or two after getting the vaccine. You may experience other side effects that are not anticipated at this time. As with any drug, there is a very small possibility that you could experience an allergic reaction to the vaccine.

You may also experience some discomfort from the tests that are involved. It is possible to have bruising and tenderness at the site where blood is taken. Fainting

while blood is being taken or local infection at the puncture site may also occur, although these are rare.

The safety of Fluviral vaccine is established in pregnancy and breast feeding. As such, no special measures are required in this study.

The side effects of the double dose of vaccine is anticipated to be very similar to the single dose, however, in any research study there is always the possibility that harmful side effects which are not known at this time may occur. In the event that an injury occurs as a direct result of the administration of the study vaccine, necessary medical care will be made available to you.

OTHER MEDICATIONS/VACCINES

At no time during the study should you receive an investigational drug or other vaccine except as prescribed by the study doctor (study directions) or as indicated by medical need. Please phone the study doctor/research coordinator before you take any new medications or vaccines.

BENEFITS

The potential benefit from participating in this study is to have immunity that would protect you from becoming infected by one or more of the viruses contained in the 2008 Fluviral vaccine. In addition, your participation may lead to the development of a safer and more effective vaccine against this disease.

ALTERNATIVES TO THE STUDY

Should you wish to be vaccinated against influenza, you may choose to receive commercially available Fluviral vaccine through your physician. You may also choose to not receive any vaccine for influenza.

COSTS/COMPENSATION

Participation in this study will not result in any expenses to you. The study procedures will be provided at no charge and there will be no charge to you for study-related clinical examinations, laboratory tests or materials needed.

In recognition of your time commitment to the study, transportation costs and any inconvenience that you may experience, you will receive $ 15.00 for parking at each completed visit, for up to $75.00 if you complete all 5 visits.

COMPENSATION FOR INJURY

In the event of a research-related injury, you will be provided with appropriate medical treatment. You are not waiving your legal rights by agreeing to participate in this study. The study doctor, the study sponsor and the hospital still have their legal and professional responsibilities.

REIMBURSEMENT FOR MEDICAL TREATMENT

In the event of a study related injury, you should contact the Study Doctor and Study Site listed on the cover page of this informed consent. If you are injured as a result of your participation in this study, <Insert Dr. Name> and the <Insert Institution Name> will ensure that adequate medical care is provided to you. No form of compensation is routinely available from this research facility. You will not give up any legal rights by signing this form.

CONFIDENTIALITY

The research staff will endeavour to keep all information that is learned about you private. Study information sent to our data manager will not include information that directly identifies you. Instead, a code number is assigned to the study information. Study staff will have access to your study and medical records, which contain information that directly identifies you. In addition, your records may be reviewed by representatives from The <Insert REB Name>, the regulatory authorities in Canada, or the sponsor for audit purposes. These representatives will not copy information that identifies you. By signing this written informed consent form, you consent to this access. If the results of the study are published in the medical literature, the publications will not contain any information which would identify you. No documents bearing your name will leave <Insert Institution Name>. Study records will be stored in a locked area and will be kept for a minimum of 25 years, which meets or exceeds the requirements of regulatory agencies in Canada.

RESEARCH RIGHTS

Participation in the study is entirely voluntary (your choice). You may decide not to enroll or you may withdraw from the study at any time with no penalty or loss of benefits.

You should tell your study doctor if you decide to stop the study early. If you choose to withdraw, or are withdrawn from study participation, you will be asked to return to the study clinic for a final visit assessment as part of the study's ongoing safety evaluations. If the study is changed in any way that could affect your decision to continue to

participate, you will be told about the changes and you may be asked to sign a new informed consent.

The study doctor may take you out of the study without your consent if the research is not helping you, if you do not follow the study plan/procedures, or if you have a side effect to the study vaccine.

You will be informed of the results as soon as they become available.

QUESTIONS

If you have any questions or concerns following your enrollment, you should contact the study staff. You may call <Insert Contact>. In an emergency you should contact <Insert Contact>. If you cannot reach your study doctor or study personnel for any reason, you should go immediately to the Emergency Department of your local hospital.

If you have questions about your rights as a research participant, you may contact the Chairman of The <Insert REB Name> at <Insert Contact>. This study has been approved by the <Insert REB Name> which is composed of a group of people who perform independent review of all research involving humans at <Insert Institution Name>.

PARTICIPANT'S CONSENT FORM

I have read or had read to me this 9-page (including this page) information and consent form and have had the chance to ask questions which have been answered to my satisfaction before signing my name. The nature of the study and the potential risks of reactions have been explained to me. I have the right to withdraw from the study at any time without affecting my care in any way. A copy of the signed Information and Consent Form will be given to me. I freely agree to participate in this research study.

Participant Consent:

____
Printed Name of Participant

____________________________________ ____________________
Signature of Participant Date/Time

Statement of Person Obtaining Informed Consent:

I have explained the nature and demands of the research study to the participant named above, answered their questions and have witnessed completion of the consent form.

____________________________________ ____________________
Printed Name of Person Obtaining Informed Consent Position

____________________________________ ____________________
Signature of Person Obtaining Informed Consent Date/Time

Appendix F: Product Monograph

PRODUCT MONOGRAPH

FLUVIRAL® (2007-2008)

INFLUENZA VIRUS VACCINE
TRIVALENT, INACTIVATED
SPLIT-VIRION
PREPARED IN EGGS

For active immunization against influenza
Strains: A/Solomon Islands/3/2006
A/Wisconsin/67/2005
B/Malaysia/2506/2004

ID Biomedical Corporation
GlaxoSmithKline Biologicals North America
Quebec City, QC
Canada G1P 4R8

Date of Approval: May 2, 2007

Control No.: 113239

Table of Contents

PART I: HEALTH PROFESSIONAL INFORMATION **3**
SUMMARY PRODUCT INFORMATION 3
DESCRIPTION 3
INDICATIONS AND CLINICAL USE 3
CONTRAINDICATIONS 6
WARNINGS AND PRECAUTIONS 6
ADVERSE REACTIONS 8
DRUG INTERACTIONS 10
DOSAGE AND ADMINISTRATION 11
ACTION AND CLINICAL PHARMACOLOGY 12
STORAGE AND STABILITY 13
SPECIAL HANDLING INSTRUCTIONS 13
DOSAGE FORMS, COMPOSITION AND PACKAGING 13
PART II: SCIENTIFIC INFORMATION **14**
PHARMACEUTICAL INFORMATION 14
REFERENCES 15
PART III: CONSUMER INFORMATION **17**

FLUVIRAL® (2007-2008)

Influenza Virus Vaccine
Trivalent, Inactivated Split-Virion
Prepared in Eggs

PART I: HEALTH PROFESSIONAL INFORMATION

SUMMARY PRODUCT INFORMATION

Route of Administration	Dosage Form / Strength	Clinically Relevant Nonmedicinal Ingredients
IM	Parenteral/ 15 µg influenza virus Hemagglutinin/strain/ 0.5 mL dose	Thimerosal, trace amounts of egg proteins and sodium deoxycholate. *For a complete listing see Dosage Forms, Composition and Packaging section.*

DESCRIPTION

FLUVIRAL® is a trivalent, split-virion influenza vaccine prepared from virus grown in the allantoic cavity of embryonated hens' eggs. The virus is inactivated with ultraviolet light treatment followed by formaldehyde, purified by centrifugation and disrupted with sodium deoxycholate. FLUVIRAL® is used for active immunization against influenza strains A/Solomon Islands/3/2006, A/Wisconsin/67/2005 and B/Malaysia/2506/2004.

INDICATIONS AND CLINICAL USE

FLUVIRAL®, split-virion influenza vaccine, is indicated for the active immunization against influenza strains A/Solomon Islands/3/2006, A/Wisconsin/67/2005 and B/Malaysia/2506/2004.

The National Advisory Committee on Immunization (CCDR, June 15, 2006) recommends administration of influenza vaccines to the following three groups:

1. People at high risk of influenza-related complications

- Adults and children with selected chronic health conditions if significant enough to require regular medical follow-up or hospital care. These high-risk conditions include the following:
  - cardiac or pulmonary disorders (including bronchopulmonary dysplasia, cystic fibrosis, and asthma)
  - diabetes mellitus and other metabolic diseases
  - cancer, immunodeficiency, immunosuppression (due to underlying disease and/or therapy)
  - renal disease
  - anemia or hemoglobinopathy
  - conditions that compromise the management of respiratory secretions and are associated with an increase risk of aspiration
  - children and adolescents with conditions treated for long periods with acetylsalicylic acid
- People of any age who are residents of nursing homes and other chronic care facilities
- People ≥ 65 years of age
- Healthy Children aged 6 to 23 months

2. People capable of transmitting influenza to those at high risk of influenza-related complications

- Health care and other care providers in facilities and community settings who, through their activities, are potentially capable of transmitting influenza to those at high risk of influenza complications.
- Household contacts (adults and children) of people at high risk of influenza complications, whether or not they have been immunized. These persons include household contacts of children <6 months of age (who are at high risk of complications from influenza but for whom there is no available effective vaccine) and of children aged 6 to 23 months. Pregnant women should be immunized in their third trimester if they are expected to deliver during influenza season, as they will become household contacts of their newborn.
- Those providing regular child care to children aged 0 to 23 months, whether in or out of the home
- Those who provide services within closed or relatively closed settings to persons at high risk (e.g. crew on ships).

3. Others

- People who provide essential community services
- People in direct contact with avian-influenza-infected poultry during culling operations
- Healthy persons aged 2 to 64 years, who should be encouraged to receive the vaccine, even if they are not in one of the aforementioned priority groups.

Pediatrics: Healthy children aged 6 to 23 months are at increased risk of influenza-associated hospitalization compared with healthy older children and young adults. Children and adolescents (aged 6 months to 18 years) treated for long periods with ASA may be at increased risk of Reye Syndrome after influenza infection.

Geriatrics (≥ 65 years of age): The risk of severe morbidity and mortality related to influenza is moderately increased in healthy persons over 65 years of age but is not nearly as great as in persons with chronic underlying disease.

HIV-Infected Persons: Limited information exists regarding the frequency and severity of influenza illness among HIV-infected persons, but reports suggest that symptoms may be prolonged and the risk for complications increased for some HIV-infected persons. Because influenza can result in serious illness and complications, vaccination is a prudent precaution and will result in protective antibody levels in many recipients. However, the antibody response to vaccine may be low in persons with advanced HIV-related illnesses; giving a second dose of vaccine 4 or more weeks after the first does not improve the immune response for these persons. Further studies are also required to determine whether influenza immunization can adversely affect patients infected with HIV. To date, some studies indicate that influenza immunization can be associated with transient increases in plasma HIV concentration, but no study has demonstrated an adverse effect of this temporary change on HIV disease progression.

Pregnant women: Vaccination is recommended for pregnant women in high-risk groups (see above section). Vaccine is considered safe for pregnant women - regardless of their stage of pregnancy. Although excess morbidity and mortality were observed among pregnant women during the pandemic outbreaks in 1918-19 and 1957-58, further studies are needed to determine whether pregnancy per se is a risk factor that warrants routine influenza immunization. Pregnant women should be immunized in their third trimester if they are expected to deliver during influenza season, as they will become household contacts of their newborn (children < 6 months of age are at increased risk of complications from influenza).

Breast-feeding mothers: Influenza immunization does not adversely affect the health of breast-feeding mothers or their infants. Breast-feeding is not a contraindication for influenza immunization.

People at high risk of influenza complications embarking on foreign travel to destinations where influenza is likely to be circulating should be vaccinated with the most current available vaccine. In the tropics, influenza can occur throughout the year. In the southern hemisphere, peak activity occurs from April through September. In the northern hemisphere, peak activity occurs from November through March.

Employers and their employees should consider yearly influenza immunization for healthy working adults as this has been shown to decrease work absenteeism because of respiratory and other illnesses.

Concern has been raised regarding the possibility that a pandemic influenza strain may emerge through human-avian gene reassortment within workers directly involved in poultry culling operations, who may become simultaneously infected with a human influenza virus strain and an avian influenza virus strain. This is a theoretical concern, given that this gene reassortment has not been documented to date. FLUVIRAL® protects against human but not avian influenza strains. Immunization is recommended for those directly involved in the destruction (culling) of avian influenza-infected poultry before the culling operation. Direct involvement may be defined as sufficient contact with infected poultry to allow transmission of avian virus to the exposed person. The relevant individuals include those performing the cull as well as others (such as supervising veterinarians and inspectors) who may be directly exposed to the avian virus. Those persons who would be expected by reason of their employment to come into direct contact with infected poultry during culling operations in the event of potential avian influenza outbreaks should be immunized with trivalent influenza vaccine on a yearly basis prior to the human influenza season (CCDR, June 15, 2006).

CONTRAINDICATIONS

- Known or suspected hypersensitivity to FLUVIRAL®, to thimerosal, or to any other ingredient in the formulation or component of the container. For a complete listing, see the **DOSAGE FORMS, COMPOSITION AND PACKAGING** section.

- Vaccination is not recommended for subjects who develop anaphylactic type reactions when they eat eggs (urticaria (hives), oedema of the mouth and throat, difficulty in breathing, hypotension and shock). Allergic reactions are extremely rare and usually attributable to extreme sensitivity to certain components of the vaccine, probably to trace amounts of residual egg protein. Subjects whose allergy to eggs is not of the anaphylactic type, as well as those who are allergic to chicken and to feathers may be vaccinated.

- Subjects with an acute respiratory infection or with any other active infection or serious febrile illness. On the other hand, a minor indisposition such as a mild infection of the upper respiratory tract is not necessarily a contraindication to vaccination.

- Immunization should be delayed in a patient with an active neurologic disorder, but should be considered when the disease process has been stabilized.

WARNINGS AND PRECAUTIONS

Serious Warnings and Precautions
• Sterile epinephrine hydrochloride solution 1:1000 should always be readily available in case an acute anaphylactic reaction should occur.

General

Increase of serum theophylline to toxic levels following the administration of influenza vaccine has been recorded in individuals who take oral theophylline as a maintenance therapy. Some doctors recommended a cessation of theophylline or a reduction in dose for 24 hours following

vaccination.

The administration of influenza vaccine may also delay the hepatic metabolism of other medications such as oral anticoagulants.

False-positive HIV antibody tests were reported after immunization with the 1991/92 influenza vaccines. However, the incidence of false-positive tests declined with the development of different tests so that such false-positive HIV antibody tests are not likely to be a problem now.

Immune
It is possible that the protective immune response following influenza vaccination may not develop in subjects undergoing immunosuppressive therapy.

Corticosteroid therapy can result in immunosupression although the exact dose and duration of therapy required to suppress the immune system is not well defined. Persons treated with high doses of systemic steroids, e.g., ≥ 2 mg/kg/day of prednisone orally for more than 2 weeks, or ≥ 60 mg prednisone/day in an adult, should be considered to have a compromised immune system.

Local Skin Reactions at Vaccination Sites
Soreness and redness at the injection site may occur and may last for up to two days. Prophylactic acetaminophen may decrease the frequency of pain at the injection site.

Respiratory
Revaccination of individuals who have previously experienced oculo-respiratory symptoms is safe. Previously affected individuals should be encouraged to be revaccinated. The risk of recurrence of oculo-respiratory symptoms after revaccination is minimal compared to the serious threat posed by influenza. Please refer to most current NACI recommendations regarding revaccination of subjects who experienced more severe oculo-respiratory syndrome (see references 10 and 11).

Special Populations

Pregnant Women: The National Advisory Council on Immunization considers influenza vaccine safe in pregnancy.

Pediatrics: In infants < 6 months of age, influenza vaccine is less immunogenic than in infants and children aged 6 to 18 months. Therefore, immunization with currently available influenza vaccine is not recommended for infants < 6 months.

ADVERSE REACTIONS

Adverse Drug Reaction Overview

Subvirion, or split-virion, vaccines contain purified portions of the virus rather than the entire virus. Generally, these have been shown to be associated with fewer adverse effects in children and young adults, while maintaining immunogenicity similar to that of whole virus preparations. Because of their lower rates of side effects, only split virus preparations are recommended for children under 13 years of age.

Immediate, allergic-type responses, such as hives, angioedema, allergic asthma, or systemic anaphylaxis occur extremely rarely. These reactions probably result from sensitivity to some vaccine component - most likely residual egg proteins (see **CONTRAINDICATIONS**).

The most common FLUVIRAL® adverse drug reactions are soreness at the injection site, headache and muscle aches. Reactions are generally mild and of limited duration. Prophylactic acetaminophen may decrease the frequency of some side effects in adults.

Clinical Trial Adverse Drug Reactions

Because clinical trials are conducted under very specific conditions the adverse reaction rates observed in the clinical trials may not reflect the rates observed in practice and should not be compared to the rates in the clinical trials of another drug. Adverse drug reaction information from clinical trials is useful for identifying drug-related adverse events and for approximating rates.

The data in the table below have been derived from three studies with three lots of ID Biomedical split-virion vaccine (A, B, C) compared to another subvirion vaccine (D) and to a whole virion vaccine (E) from ID Biomedical.

Percentage of subjects in each group reporting symptoms

	**Young Adults (19-45 years)**					**Children (3-12 years)**		**Elderly (over 65 years)**		
Lots	A	B	C	D	E	A	D	B	D	E
**n (subjects)**	54	56	54	56	56	65	65	58	57	57
**Local Reactions (%)**										
Soreness	72	71	68	75	95	57	58	24	21	25
Redness	22	27	26	18	27	12	14	3	3	5
Swelling	15	4	13	10	23	15	22	7	5	5
Limitation of movement	22	16	13	21	30	12	14	3	3	4
**Systemic Reactions (%)**										
Headache	37	20	24	20	34	15	17	29	14	9
Loss of appetite	11	5	5	2	11	12	8	7	5	5
Muscle aches	26	20	22	19	30	14	11	19	9	12
Chills	15	14	7	0	12	3	6	21	16	5
Nausea	13	3	2	7	14	3	3	9	9	5
Vomiting	2	0	0	0	2	1	0	3	0	0
Diarrhea	13	3	5	2	11	6	6	3	5	2
Redness/Rash	15	0	0	3	5	3	3	2	2	2

Local and systemic reactions are reported after vaccination with a split-virion influenza vaccine.

There were very few reports of fever as defined by temperature over 38°C.

Soreness at the injection site was the most frequently reported symptom, and was generally rated as mild and resolved the day after vaccination.

For systemic symptoms, headache and muscle aches were the most common. As with local symptoms, these were generally reported as mild and of limited duration.

Prophylactic acetaminophen may decrease the frequency of some side effects in adults.

Post-Market Adverse Drug Reactions

Oculorespiratory Syndrome (ORS) has been reported in Canada, US and Europe following administration of influenza vaccines. The symptoms associated with the ORS are red eyes, respiratory symptoms and facial oedema. Most cases are mild in severity and resolve spontaneously regardless of the influenza vaccine administered.

Revaccination of subjects with history of ocular or respiratory symptoms is considered to be safe regardless of the influenza vaccine used for the initial vaccination or the revaccination. Since the 2000-2001 influenza season when the symptom was first identified, the incidence of ORS has slowly declined and reporting rates are returning to background levels reported prior to 2000.

There have been reports of other neurological illnesses, including facial paralysis, encephalitis, encephalopathy, demyelinating disease and labyrinthitis, associated with other influenza vaccines. Any relationship, other than temporal, to the vaccine has not been established.

Unlike the 1976-77 swine influenza vaccine, subsequent vaccines prepared from other virus strains have not been clearly associated with an increased frequency of Guillain-Barré syndrome. Influenza vaccine is not known to predispose to Reye's syndrome.

Notification of reactions

It is desirable that all unusual reactions, arising from any vaccination whatsoever, or following shortly thereafter, be reported to the manufacturer of the product and to the provincial epidemiologist.

DRUG INTERACTIONS

Drug-Drug Interactions

The metabolism of oral theophylline or oral anticoagulants may be affected by vaccination with FLUVIRAL® (see **WARNINGS AND PRECAUTIONS**).

The target groups for influenza and pneumococcal vaccination overlap considerably. Health care

providers should take the opportunity to vaccinate eligible persons against pneumococcal disease during the same visit at which influenza vaccine is given. The concurrent administration of the two vaccines at different sites does not increase the risk of side effects. Pneumococcal vaccine, however, is not administered annually, as in the case of influenza vaccine.

Children at high risk may receive influenza vaccine at the same time but at a different site from that used for routine pediatric vaccines.

DOSAGE AND ADMINISTRATION

Recommended Dose and Dosage Adjustment

Influenza vaccine dosage, by age group

Age Group	Dosage	Route*
6 - 35 months	1 x 0.25 mL or 2 x 0.25 mL•	IM
3 - 8 years	1 x 0.50 mL or 2 x 0.50 mL•	IM
9 years and older	1 x 0.50 mL	IM

*The recommended site of vaccination is the deltoid muscle for adults and older children. The preferred site for infants and young children is the anterolateral aspect of the thigh.
•Two doses administered at least one month apart are recommended for children younger than 9 years of age receiving influenza vaccine for the first time.

Since the likelihood of febrile convulsions is greater in children aged 6 to 35 months, special care should be taken in weighing relative risks and benefits in this group.

Check the expiry date of the vaccine carefully. Any vaccine beyond its expiry date should not be used.

Administration

FLUVIRAL® vaccine must not be administered intravenously.

Shake the multidose vial vigorously each time before withdrawing a dose of vaccine.

Proper aseptic technique should be used for withdrawal of each dose from the multidose vial. Once entered, return the multidose vial to the recommended storage conditions, between 2°C and 8°C. Once entered, the multidose vial should be discarded after 28 days.

A separate sterile 1-cc syringe and needle or a sterile disposable 1-cc unit should be used for each injection to prevent transmission of hepatitis B, HIV, or other infectious agents from one person to another.

Disinfect the skin at the site of injection with a suitable antiseptic and wipe dry with sterile cotton wool. The injection of FLUVIRAL® **should be given intramuscularly**, usually into the deltoid muscle. **Do not inject influenza vaccine intravenously.**

All vaccinees should be observed for about 15 minutes after vaccination. If an anaphylactic reaction develops, sterile epinephrine hydrochloride (1:1000) should be administered.

OVERDOSAGE

In a study by Matzkin and Nili (1984), following administration of a dose of flu vaccine 10 times greater than the recommended dose of 0.5 mL, adverse events were not significantly different between study and control subjects.

There have been reports of patients who received higher than recommended doses of FLUVIRAL®. The adverse events noted in these patients were similar to those reported from patients who had received the recommended dose.

ACTION AND CLINICAL PHARMACOLOGY

Mechanism of Action

FLUVIRAL®, split-virion inactivated influenza vaccine, promotes an active immunization against influenza strains A (H1N1 and H3N2) and B. Within seven days after injection of the vaccine there is an increase in circulating antibody to the viral hemagglutinin and peripheral blood lymphocytes are primed to respond to in vitro stimulation by vaccine antigens. As with other inactivated influenza vaccines, immunization is based on the humoral component of the specific immunological defense system, namely immunoglobulin G (IgG) antibodies against viral hemagglutinin (HA) and neuraminidase antigens. The effectiveness of inactivated influenza vaccines correlates with the age and immunocompetence of the vaccine recipient and the degree of similarity between the virus strains used in the preparation of the vaccines and those prevailing in the population.

Cytotoxic T lymphocyte response occurs after administrations of either killed or live virus vaccines and is detectable in the absence of demonstrable antibody response.

Pharmacodynamics/Pharmacokinetics

No pharmacodynamics studies and no pharmacokinetics studies have been conducted with FLUVIRAL® in accordance with its status as a vaccine.

Duration of Effect

Both humoral and cell-mediated responses are thought to play a role in immunity to influenza. Immunity declines over the year following vaccination. The production and persistence of antibody after vaccination depends on numerous factors, including age, prior and subsequent exposure to antigens, presence of immunodeficiency states, and polymorphisms in HLA class II molecules. Humoral antibody levels, which correlate with vaccine protection, are generally achieved by 2 weeks after immunization. It is postulated that immunity after administration of the inactivated vaccine lasts < 1 year. However, in the elderly, antibody levels may fall below protective levels within 4 months. Data are not available to support a recommendation for the

administration of a second dose of influenza vaccine in elderly individuals in order to boost immunity. (CCDR, June 15, 2006)

STORAGE AND STABILITY

FLUVIRAL® must be stored between 2°C and 8°C.

Do not freeze. Freezing destroys activity. Do not use vaccine that has been frozen.

Do not use vaccine after expiration date.

Once entered, the multidose vial should be discarded after 28 days.

SPECIAL HANDLING INSTRUCTIONS

The vaccine should be well shaken prior to use (see **DOSAGE AND ADMINISTRATION** section).

FLUVIRAL® and materials used during vaccination should be disposed of in the same way as other drugs administered by injection. Since split-virion influenza vaccine is an inactivated vaccine, it presents no risk of contaminating the work area during manipulation.

DOSAGE FORMS, COMPOSITION AND PACKAGING

The composition of FLUVIRAL® is established in agreement with the recommendations of the Canadian National Advisory Committee on Immunization (NACI) and the World Health Organization (WHO).

For the 2007-2008 season, each dose of 0.5 mL of the split-virion influenza vaccine contains:

15 µg hemagglutinin of Strain A/Solomon Islands/3/2006 (H1N1),
15 µg hemagglutinin of Strain A/Wisconsin/67/2005 (H3N2),
15 µg hemagglutinin of Strain B/Malaysia/2506/2004.

The vaccine also contains 0.01% thimerosal as a preservative, and trace residual amounts of egg proteins and sodium deoxycholate. Antibiotics are not used in the manufacture of this vaccine.

FLUVIRAL® is supplied in 5 mL vials holding 10 x 0.5 mL doses.

PART II: SCIENTIFIC INFORMATION

PHARMACEUTICAL INFORMATION

Drug Substance

FLUVIRAL® vaccine is a whitish, slightly opalescent, liquid.

Product Characteristics

FLUVIRAL® is a trivalent, split-virion inactivated influenza vaccine prepared from virus grown in the allantoic cavity of embryonated hens' eggs. The virus is inactivated with UV and formaldehyde, purified by centrifugation and disrupted with sodium deoxycholate. FLUVIRAL® is for intramuscular use only.

REFERENCES

1. Al-Mazrou, A., Scheifele, D.W., Soong, T. et al.: Comparison of Adverse Reactions to Whole-Virion and Split-Virion Influenza Vaccine in Hospital Personnel. Can. Med. Assoc. J. 1991; 145: 213-218.

2. Data filed at ID Biomedical. Source of information: Division of Immunization and Respiratory Diseases, Population and Public Health Branch, Health Canada. November 20, 2003.

3. De Serres, G., Boulianne, N., Duval, B. et al.: Oculo-respiratory syndrome following influenza vaccination: evidence for occurrence with more than one influenza vaccine. Vaccine 2003; 21: 2346-2353.

4. Fischer, R.G. et al.: Influence of Trivalent Influenza Vaccine on Serum Theophylline Levels. Can. Med. Assoc. J. 126(11): 1312-13, 1982.

5. Grenier, J.L., Toth, E., De Serres, G. et al: Safety of Revaccination of Patients Affected by the Oculo-Respiratory Syndrome (ORS) Following Influenza Vaccination. Canada Communicable Diseases Report 2004; 30: 9-16.

6. Gross, P.A., Ennis, F.A., Gaeslan, F.F., et al.: A Controlled Double-Blind Comparison of Reactogenicity, Immunogenicity and Protective Efficacy of Whole and Split-Product Influenza Vaccine in Children. J. Infect. Dis. 1977; 136: 623-632.

7. Jenning, R., Clark, A., Oxford, J.S. et al.: Reactogenicity and Immunogenicity of Whole and Ether-Tween Split Influenza A Virus Vaccines in Volunteers. J. Infect. Dis. 1978; 138: 577-586.

8. Kramer, P., McClain, C.J.: Depression of Aminopyrine Metabolism by Influenza Vaccination. New Engl. J. Med., 305(21): 1262-64, 1981.

9. Matzkin, H. and Nili, E.: Accidental Tenfold Overdose of Influenza Vaccine: A Clinical and Serological Study. Israel J. Med. Sci. 20: 411-415, 1984

10. National Advisory Committee on Immunization (NACI). ORS Following Influenza Vaccination: Review of Post-Marketing Surveillance through Four Influenza Seasons in Canada, 31 (21), 1 November 2005.

11. National Advisory Committee on Immunization (NACI): Statement on Influenza Vaccination for the 2007-2008 Season. Canada Communicable Disease Report, http://www.phac-aspc.gc.ca/publicat/ccdr-rmtc. To be published in 2007.

12. Plotkin, S.A., Mortimer, E.A. Jr. et al.: Inactivated Influenza Vaccine, Vaccines, W.B. Saunders Co., Philadelphia, 1988; 426-427.

13. Renton, K.W. et al.: Decreased Elimination of Theophylline After Influenza Vaccination. Can. Med. Assoc. J. 123(4): 288-90, 1980.

14. Report of the Committee on Infectious Diseases, 22 ed., American Academy of Pediatrics, Elk Grove Village, IL, 1991: 274-281.

15. Scheifele, D.W., Duval, B., Russell, M.L. et al.: Ocular and Respiratory Symptoms Attributable to Inactivated Split Influenza Vaccine: Evidence from a Controlled Trial Involving Adults. Clinical Infectious Diseases 2003; 36: 850-857.

16. Skowronski, D.M., Strauss, B., Kendall P. et al.: Low risk of recurrence of oculorespiratory syndrome following influenza revaccination. Canadian Medical Association Journal 2002; 167: 853-858.

17. Walker, S. et al.: Serum Theophylline Levels After Influenza Vaccination (letter). Can. Med. Assoc. J. 125(3): 243-44, 1981.

18. WHO Global Advisory Committee on Vaccine Safety (Dec. 16-17, 2002). WHO Weekly Epidemiological Record 2003; 78: 17-20.

ID Biomedical Corporation
GlaxoSmithKline Biologicals North America
Quebec City, QC
Canada G1P 4R8

IMPORTANT: PLEASE READ

PART III: CONSUMER INFORMATION

FLUVIRAL® (2007-2008)

Influenza Virus Vaccine
Trivalent, Inactivated Split-Virion
Prepared in Eggs

This leaflet is part III of a three-part "Product Monograph" and is designed specifically for Consumers. This leaflet is a summary and will not tell you everything about FLUVIRAL®. If you have any questions, or if you are not sure about anything, ask your doctor, nurse or pharmacist.

Please read this leaflet carefully before receiving FLUVIRAL® as it contains information about the vaccine. It may be useful to keep this leaflet in case you need to read it again after vaccination.

ABOUT THIS MEDICATION

What is Influenza?

- Influenza is a contagious disease of the upper respiratory tract
- It is caused by a virus
- Influenza is spread by nasal droplets
- Symptoms can include sudden fever, headache, chills, muscle aches and cough
- Occurs in Canada every year during late fall and winter months
- Occurs worldwide (globally)

Vaccination is the principal means of influenza prevention and associated complications.

What is FLUVIRAL® and what does it do?
FLUVIRAL® is a vaccine against influenza. It is an inactivated (killed) influenza (flu) virus vaccine in suspension for injection, which has been prepared in hens' eggs. The vaccine is made from the strains of flu virus which are expected in the coming winter. It is normally given in the autumn to protect you in the winter.

Flu immunization gives good protection against flu, and lasts for about one year. In order to be protected against the flu, you need to be given yearly injections of the vaccine.

Flu immunization does not *cause* illness. It is a coincidence if you develop a cough or cold shortly after having a flu immunization.

Flu immunization does not prevent other virus infections that can cause coughs and colds. It protects only against the influenza virus that is expected in the coming winter.

Who should receive the vaccine?
FLUVIRAL® should be given annually in the fall to the following people:

- People over 65 years of age
- Healthy children aged 6 to 23 months
- Healthy persons aged 2 to 64 years
- Adults and children with chronic heart or lung disorders
- Adults and children with chronic conditions such as: diabetes and other metabolic diseases, cancer, immunodeficiency, immunosuppression, renal disease, anemia
- People of any age who are residents of nursing homes and/or chronic care facilities
- People with HIV
- Pregnant women with the above conditions
- Adolescents and children (ages 6 months to 18 years) treated for long periods with ASA
- Health care providers who work in facilities and community settings, such as physicians, nurses, and emergency response workers
- Health care and other service providers who have contact with residents of continuing care facilities or residences
- People who provide home care for persons in high-risk groups
- People who provide services within closed or relatively closed settings to persons at high risk
- Household contacts (adults and children) of people at high risk of flu complications (including contacts of children <6 months and children 6 to 23 months) as well as pregnant women expected to deliver during flu season.
- People providing regular childcare to children aged 0 to 23 months, whether in or out of the home
- People at risk of flu complications who will be travelling to an area where the flu is likely to occur
- People directly involved in the destruction of poultry infected with avian influenza ("bird flu").

What Is In Your Medication?

- Each 0.5 mL dose of the vaccine contains 15 micrograms of highly purified sub-units (hemagglutinin) of strain A/Solomon Islands/3/2006 (H1N1), A/Wisconsin/67/2005 and B/Malaysia/2506/2004. These are the flu viruses that are likely to cause flu this winter.
- FLUVIRAL® vaccine also contains very small amounts of egg protein, thimerosal, and sodium deoxycholate.

What Dosage Forms Does Your Medication Come In?
FLUVIRAL® is supplied in 5 mL vials holding 10x 0.5 mL doses.
FLUVIRAL® vaccine packaging does not contain latex.

IMPORTANT: PLEASE READ

WARNINGS AND PRECAUTIONS

Who should not receive the vaccine?

- People who have had a life-threatening allergic reaction to the vaccine or any of its components (e.g. egg, thimerosal)
- People with moderate to severe illness may have to delay immunization
- People who take oral theophylline or oral anticoagulants should consult with their physician before immunization
- Children under the age of 6 months

Make sure your prescriber knows if you have any of the following:

- any kind of infection or a high temperature at the moment
- a weakened immune system due to illness
- you are taking medicines which weaken the immune system (e.g. steroids such as prednisone)

If you have previously experienced oculo-respiratory symptoms, such as red eyes, respiratory symptoms and facial swelling, after having received a flu vaccine, you can receive the vaccine again this year. The risk of having oculo-respiratory symptoms again after revaccination is minimal compared to the serious threat posed by the flu.

INTERACTIONS WITH THIS MEDICATION

The metabolism of oral theophylline or oral anticoagulants may be affected by vaccination with FLUVIRAL® (see ***Who should not receive the vaccine***).

FLUVIRAL® may be given at the same time as other vaccines, such as the vaccine against pneumococcal disease and routine pediatric vaccines. The vaccines should be injected at different sites to reduce the risk of side effects.

PROPER USE OF THIS MEDICATION

Usual Dose

Children 6-35 months: one dose of 0.25 mL

Children (>3 years) and adults: one dose of 0.5 mL

Note: Children under 9 years of age require two doses, one month apart, the first time they have the vaccine

SIDE EFFECTS AND WHAT TO DO ABOUT THEM

As with all medicines, FLUVIRAL® may cause side effects in some persons. If any side effects worry you, or you have any unusual symptoms, please contact your doctor, nurse or pharmacist.

As with all injectable vaccines, appropriate medical treatment and supervision should always be readily available in cases of very rare but serious allergic reactions. This would normally happen immediately after the injection had been given - please tell the nurse if you get a rash, have tightness in the throat or shortness of breath.

If you notice any other side effects not mentioned below, please inform your doctor, nurse or pharmacist.

You may notice some pain, reddening or swelling at the site of the injection. In some cases you may feel unwell and experience fever or swelling of the lymph glands. More rarely headache, shivering, sweating, tiredness and aches in your muscles and joints may occur. In addition, red eyes, respiratory problems and facial swelling may occur. These reactions are usually mild and should only last a day or two.

You should tell your doctor if you get any of the following unwanted effects: nerve pain (neuralgia), numbness / pins and needles (possibly with fever), convulsions, unexplained or easy bruising, skin rash, urinary symptoms

HOW TO STORE IT

FLUVIRAL® should be stored in the refrigerator at +2° C to +8° C (Do Not Freeze). Do not use vaccine that has been frozen. Do not use vaccine after expiration date.

REPORTING SUSPECTED SIDE EFFECTS

To monitor drug safety, Health Canada collects information on all side effects experienced with vaccines. If you suspect you have had an adverse reaction to this vaccine you may notify Health Canada by:

Toll-free telephone: 1- 866-234-2345
Toll-free fax: 1-866-678-6789
By email: cadrmp@hc-sc.gc.ca

By regular mail:
Vaccine Safety Section
Division of Immunology
Center for Infectious Disease and Prevention Control (CIDPC)
3rd floor, CIDPC Building A/L 0603E1
Tunney's Pasture
Ottawa, Ontario
K1A 0L2

NOTE: Before contacting Health Canada, you should contact your physician or pharmacist.

MORE INFORMATION

This document plus the full product monograph, prepared for health professionals can be obtained by contacting the sponsor, GlaxoSmithKline Customer Service. Tel: 1-800-387-7374.

This leaflet was prepared by:
ID Biomedical Corporation
GlaxoSmithKline Biologicals North America
Quebec City, QC
Canada G1P 4R8

Last revised: 26 April 2007

Appendix G: CTN Toxicity Table for Grading Adverse Experiences

PARAMETER	GRADE 1<br>MILD	GRADE 2<br>MODERATE	GRADE 3<br>SEVERE	GRADE 4<br>POTENTIALLY LIFE THREATENING
POTASSIUM				
Hyperkalemia	5.6 - 6.0 meq/L OR<br>5.6 - 6.0 mmol/L	6.1 - 6.5 meq/L OR<br>6.1 - 6.5 mmol/L	6.6 - 7.0 meq/L OR<br>6.6 - 7.0 mmol/L	>7.0 meq/L OR<br>>7.0 mmol/L
Hypokalemia	3.0 - 3.4 meq/L OR<br>3.0 - 3.4 mmol/L	2.5 - 2.9 meq/L OR<br>2.5 - 2.9 mmol/L	2.0 - 2.4 meq/L OR<br>2.0 - 2.4 mmol/L	<2.0 meq/L OR<br><2.0 mmol/L
PHOSPHATE				
Hypophosphatemia	2.0 - 2.4 mg/dL OR<br>0.63 - 0.77 mmol/L	1.5 - 1.9 mg/dL OR<br>0.46 - 0.62 mmol/L	1.0 - 1.4 mg/dL OR<br>0.32 - 0.45 mmol/L	<1.0 mg/dL OR<br><0.32 mmol/L
CALCIUM - (corrected for albumin)				
Hypocalcemia	7.8 - 8.4 mg/dL OR<br>1.95 - 2.10 mmol/L	7.0 - 7.7 mg/dL OR<br>1.74 - 1.94 mmol/L	6.1 - 6.9 mg/dL OR<br>1.53 - 1.73 mmol/L	<6.1 mg/dL OR<br><1.53 mmol/L
Hypercalcemia	10.6 - 11.5 mg/dL OR<br>2.65 - 2.87 mmol/L	11.6 - 12.5 mg/dL OR<br>2.88 - 3.14 mmol/L	12.6 - 13.5 mg/dL OR<br>3.15 - 3.37 mmol/L	>13.5 mg/dL OR<br>>3.37 mmol/L
MAGNESIUM				
Hypomagnesemia	1.2 - 1.4 meq/L OR<br>0.60 - 0.70 mmol/L	0.9 - 1.1 meq/L OR<br>0.45 - 0.59 mmol/L	0.6 - 0.8 meq/L OR<br>0.30 - 0.44 mmol/L	<0.6 meq/L OR<br><0.30 mmol/L
BILIRUBIN				
Hyperbilirubinemia	>1.0 - 1.5 X ULN	>1.5 - 2.5 X ULN	>2.5 - 5 X ULN	>5 X ULN

adapted from DAIDS/CPCRA January 1997 version

PARAMETER	GRADE 1<br>MILD	GRADE 2<br>MODERATE	GRADE 3<br>SEVERE	GRADE 4<br>POTENTIALLY LIFE THREATENING
GLUCOSE				
Hypoglycemia	55 - 64 mg/dL OR<br>3.01 - 3.55 mmol/L	40 - 54 mg/dL OR<br>2.19 - 3.00 mmol/L	30 - 39 mg/dL OR<br>1.67 - 2.18 mmol/L	<30 mg/dL OR<br><1.67 mmol/L
Hyperglycemia (nonfasting and no prior diabetes)	116 - 160 mg/dL OR<br>6.44 - 8.90 mmol/L	161 - 250 mg/dL OR<br>8.91 - 13.88 mmol/L	251 - 500 mg/dL OR<br>13.89 - 27.76 mmol/L	>500 mg/dL OR<br>>27.76 mmol/L
TRIGLYCERIDES	----------------	400-750 mg/dL OR<br>4.52 - 8.47 mmol/L	751 - 1200 mg/dL OR<br>8.48 - 13.55 mmol/L	>1200 mg/dL OR<br>>13.55 mmol/L
CREATININE	>1.0 - 1.5 X ULN	>1.5 - 3.0 X ULN	>3.0 - 6.0 X ULN	>6.0 X ULN
URIC ACID				
Hyperuricemia	7.5 - 10.0 mg/dL OR<br>446 - 595 umol/l	10.1 - 12.0 mg/dL OR<br>596 - 716 umol/l	12.1 - 15.0 mg/dL OR<br>717 - 892 umol/l	>15.0 mg/dL OR<br>>892 umol/l
LIVER TRANSAMINASE (LFTs)				
AST (SGOT)	1.25 - 2.5 X ULN	>2.5 - 5.0 X ULN	>5.0 - 10.0 X ULN	>10.0 X ULN
ALT (SGPT)	1.25 - 2.5 X ULN	>2.5 - 5.0 X ULN	>5.0 - 10.0 X ULN	>10.0 X ULN
GGT	1.25 - 2.5 X ULN	>2.5 - 5.0 X ULN	>5.0 - 10.0 X ULN	>10.0 X ULN
Alk Phos	1.25 - 2.5 X ULN	>2.5 - 5.0 X ULN	>5.0 - 10.0 X ULN	>10.0 X ULN
PANCREATIC ENZYMES				
Amylase	>1.0 - 1.5 X ULN	>1.5 - 2.0 X ULN	>2.0 - 5.0 X ULN	>5.0 X ULN
Pancreatic amylase	>1.0 - 1.5 X ULN	>1.5 - 2.0 X ULN	>2.0 - 5.0 X ULN	>5.0 X ULN
Lipase	>1.0 - 1.5 X ULN	>1.5 - 2.0 X ULN	>2.0 - 5.0 X ULN	>5.0 X ULN

adapted from DAIDS/CPCRA January 1997 version

PARAMETER	GRADE 1<br>MILD	GRADE 2<br>MODERATE	GRADE 3<br>SEVERE	GRADE 4<br>POTENTIALLY LIFE THREATENING
**CARDIOVASCULAR**				
CARDIAC ARRHYTHMIA	————	Asymptomatic; transient dysrhythmia, no Rx req	Recurrent/persistent dysrhythmia; symptomatic Rx req	Unstable dysrhythmia, hospitalization and Rx req
HYPERTENSION	Transient, increase >20 mmHg;<br>no Rx	Recurrent; chronic increase >20mmHg, Rx req	Acute Rx req; outpatient hospitalization possible	Hospitalization req
HYPOTENSION	Transient orthostatic hypotension, no Rx	Symptoms correctable with oral fluid Rx	IV fluid req, no hospitalization req	Hospitalization req
PERICARDITIS	Minimal effusion	Mild/mod asymptomatic effusion, no Rx	Symptomatic effusion, pain, EKG changes	Tamponade OR pericardiocentesis OR surgery req
HEMORRHAGE, BLOOD LOSS	————	Mildly symptomatic, no Rx required	Gross blood loss **OR** 1-2 units transfused	Massive blood loss OR >2 units transfused

adapted from DAIDS/CPCRA January 1997 version

PARAMETER	GRADE 1<br>MILD	GRADE 2<br>MODERATE	GRADE 3<br>SEVERE	GRADE 4<br>POTENTIALLY LIFE THREATENING
**GASTROINTESTINAL**				
NAUSEA	Mild OR transient; reasonable intake maintained	Mod discomfort OR intake decreased for <3 days	Severe discomfort OR minimal intake for ≥3 days	Hospitalization req
VOMITING	Mild **OR** transient; 2-3 episodes per day **OR** mild vomiting lasting <1 week	Mod **OR** persistent; 4-5 episodes per day **OR** vomiting lasting ≥1 week	Severe vomiting of all food/fluids in 24 hrs **OR** orthostatic hypotension **OR** IV Rx req	Hypotensive shock **OR** hospitalization req for IV Rx req
DIARRHEA	Mild **OR** transient; 3-4 loose stools per day **OR** mild diarrhea lasting <1 week	Mod **OR** persistent; 5-7 loose stools per day **OR** diarrhea lasting ≥1 week	Bloody diarrhea **OR** orthostatic hypotension **OR** >7 loose stools/day **OR** IV Rx req	Hypotensive shock **OR** hospitalization req
ORAL DISCOMFORT/ DYSPHAGIA	Mild discomfort, no difficulty swallowing	Difficulty swallowing but able to eat and drink	Unable to swallow solids	Unable to drink fluids; IV fluids req
CONSTIPATION	Mild	Moderate	Severe	Distention with vomiting

adapted from DAIDS/CPCRA January 1997 version

PARAMETER	GRADE 1<br>MILD	GRADE 2<br>MODERATE	GRADE 3<br>SEVERE	GRADE 4<br>POTENTIALLY LIFE THREATENING
RESPIRATORY				
COUGH (for aerosol studies)	Transient; no Rx	Treatment associated cough; inhaled bronchodilator	Uncontrolled cough; systemic Rx req	--------------
BRONCHOSPASM ACUTE	Transient; no Rx; FEV1<80% -70% (or peak flow)	Rx req; normalizes with bronchodilator; FEV1 50% - <70% (or peak flow)	No normalization with bronchodilator; FEV1 25% - <50% (or peak flow), retractions	Cyanosis; FEV1<25% (or peak flow) OR intubated
DYSPNEA	Dyspnea on exertion	Dyspnea with normal activity	Dyspnea at rest	Dyspnea requiring O_2 therapy
NEUROLOGIC				
NEURO-CEREBELLAR	Slight incoordination OR dysdiadochokinesia	Intention tremor OR dysmetria OR slurred speech OR nystagmus	Locomotor ataxia	Incapacitated
NEURO-PSYCH/MOD	----------------	----------------	Severe mood changes requiring medical intervention	Acute psychosis req hospitalization
PERIPHERAL NEUROPATHY	Peripheral neuropathy is a syndrome characterized by signs or symptoms of one or several specific neurologic manifestations:<br>neuromuscular paresis, paresthesia, neuromotor, and neurosensory abnormalities.<br><br>Peripheral neuropathy should be graded as the maximum grade, according to the DAIDS toxicity table, of any of these manifestations which, in your clinical judgment, are due to peripheral neuropathy rather than another (i.e., central) cause.			

adapted from DAIDS/CPCRA January 1997 version

PARAMETER	GRADE 1<br>MILD	GRADE 2<br>MODERATE	GRADE 3<br>SEVERE	GRADE 4<br>POTENTIALLY LIFE THREATENING
NEUROMUSCULAR PARESIS	Subjective weakness; no objective symptoms/signs	Mild objective signs, symptoms, no decrease in function	Objective weakness; function limited	Paralysis
PARESTHESIA (burning, tingling, etc.)	Mild discomfort; no Rx req	Moderate discomfort persisting for >72 hours requiring non-narcotic analgesia	Severe discomfort **OR** narcotic analgesia required with symptomatic improvement	Incapacitating **OR** intolerable discomfort, not responsive to narcotic analgesia
NEURO-MOTOR	Decrease in reflexes **OR** patients with chronic stable abnormality of reflex or use of muscle	Absence of a previously present reflex	Absence of 2-3 previously present reflexes	Absence of >3 previously present reflexes
NEURO-SENSORY	Decrease in sensation (pinprick, vibratory or hot/cold) **OR** patients with chronic **STABLE** abnormality of sensation	Absence of a previously present sensory finding (one dermatome)	Absence of 2-3 previously present sensory dermatomes	Absence of >3 previously present sensory dermatomes
**URINALYSIS**				
PROTEINURIA				
Spot urine	1+	2 - 3+	4+	Nephrotic syndrome
24 hour urine	200 mg-1 g loss/day **OR** <0.3% **OR** <3 g/l	1 - 2 g loss/day **OR** 0.3 - 1.0% **OR** 3 - 10 g/l	2 - 3.5 g loss/day **OR** >1.0% **OR** >10 g/l	Nephrotic syndrome **OR** >3.5 g loss/day
GROSS HEMATURIA	Microscopic only	Gross, no clots	Gross plus clots	Obstructive **OR** transfusion req

adapted from DAIDS/CPCRA January 1997 version

PARAMETER	GRADE 1<br>MILD	GRADE 2<br>MODERATE	GRADE 3<br>SEVERE	GRADE 4<br>POTENTIALLY LIFE THREATENING
**MISCELLANEOUS**				
FEVER (oral,>12 hours)	37.7 - 38.5°C OR 100.0 - 101.5°F	38.6 - 39.5°C OR 101.6 - 102.9°F	39.6 - 40.5°C OR 103 - 105°F	>40.5°C OR >105°F
HEADACHE	Mild; no Rx req	Moderate **OR** non-narcotic analgesia Rx	Severe **OR** responds to initial narcotic Rx	Intractable **OR** requiring repeated narcotic Rx
ALLERGIC REACTION	Pruritus without rash	Localized urticaria	Generalized urticaria angioedema	Anaphylaxis
CUTANEOUS/RASH/ DERMATITIS	Erythema, pruritus	Diffuse maculopapular rash **OR** dry desquamation	Vesiculation **OR** moist desquamation **OR** ulceration	**ANY ONE:** mucous membrane involvement, suspected Stevens-Johnson (TEN), erythema multiforme, necrosis req surgery, exfoliative dermatitis
LOCAL REACTION (2° parenteral Rx - not vaccination or skin test)	Erythema	Induration<10 mm OR inflammation OR phlebitis	Induration>10mm **OR** ulceration	Necrosis of skin
FATIGUE	Normal activity reduced <25%	Normal activity reduced 25-50%	Normal activity reduced >50%; cannot work	Unable to care for self

adapted from DAIDS/CPCRA January 1997 version

CTN Toxicity Table for Grading Adverse Experiences

May 1, 2001

PARAMETER	GRADE 1<br>MILD	GRADE 2<br>MODERATE	GRADE 3<br>SEVERE	GRADE 4<br>POTENTIALLY LIFE THREATENING
**HEMATOLOGY**				
Hemoglobin	8.0 - 9.4 g/dL OR<br>80 - 94 g/L OR<br>4.93 - 5.83 mmol/L	7.0 - 7.9 g/dL OR<br>70-79 g/L OR<br>4.31 - 4.92 mmol/L	6.5 - 6.9 g/dL OR<br>65 - 69 g/L OR<br>4.03 - 4.30 mmol/L	<6.5 g/dL OR<br><65 g/L OR<br><4.03 mmol/L
Absolute Neutrophil Count	1000 - 1500/mm^3 OR<br>1.0 - 1.5/G/L*	750 - 999/mm^3 OR<br>0.75 - 0.99/G/L*	500 - 749/mm^3 OR<br>0.5 - 0.749/G/L*	<500/mm^3 OR<br><0.5/G/L*
Platelets	75,000 - 99,000/mm^3 OR<br>75 - 99/G/L*	50,000 - 74,999/mm^3 OR<br>50 - 74.9/G/L*	20,000 - 49,000/mm^3 OR<br>20 - 49.9/G/L*	<20,000/mm^3 OR<br><20/G/L*
Prothrombin Time (PT)	>1.0 - 1.25 X ULN	>1.25 - 1.5 X ULN	>1.5 - 3.0 X ULN	>3.0 X ULN
PTT	>1.0 - 1.66 X ULN	>1.66 - 2.33 X ULN	>2.33 - 3.0 X ULN	>3.0 X ULN
Methemoglobin	5.0 - 10.0%	10.1 - 15.0%	15.1 - 20.0%	>20%
*G = $X 10^9$				
**CHEMISTRIES**				
Hyponatremia	130 - 135 meq/L OR<br>130 -135 mmol/L	123 - 129 meq/L OR<br>123 - 129 mmol/L	116 - 122 meq/L OR<br>116 - 122 mmol/L	<116 meq/L OR<br><116 mmol/L
Hypernatremia	146 - 150 meq/L OR<br>146 - 150 mmol/L	151 - 157 meq/L OR<br>151 - 157 mmol/L	158 - 165 meq/L OR<br>158 - 165 mmol/L	>165 meq/L OR<br>>165 mmol/L

adapted from DAIDS/CPCRA January 1997 version
